# Post-Ugi gold-catalyzed diastereoselective domino cyclization for the synthesis of diversely substituted spiroindolines

**DOI:** 10.3762/bjoc.9.246

**Published:** 2013-10-14

**Authors:** Amit Kumar, Dipak D Vachhani, Sachin G Modha, Sunil K Sharma, Virinder S Parmar, Erik V Van der Eycken

**Affiliations:** 1 Laboratory for Organic & Microwave-Assisted Chemistry (LOMAC), Department of Chemistry, University of Leuven (KU Leuven), Celestijnenlaan 200F, 3001, Leuven, Belgium; 2 Bioorganic Laboratory, Department of Chemistry, University of Delhi, Delhi-110 007, India; 3 Chemistry Building-4.20b, School of Chemistry, The University of Manchester, Manchester M13 9PL, UK

**Keywords:** alkynes, gold, indoles, multicomponent, spiroindolines, Ugi

## Abstract

An Ugi four-component reaction of propargylamine with 3-formylindole and various acids and isonitriles produces adducts which are subjected to a cationic gold-catalyzed diastereoselective domino cyclization to furnish diversely substituted spiroindolines. All the reactions run via an *exo*-*dig* attack in the hydroarylation step followed by an intramolecular diastereoselective trapping of the imminium ion. The whole sequence is atom economic and the application of a multicomponent reaction assures diversity.

## Introduction

The importance of nitrogen containing heterocyclic molecules in chemical biology is undisputed. The synthesis of such biologically interesting heterocycles is generally target-oriented, inspired by nature or randomly directed. In all these cases the design of a synthetic sequence to produce a library of diversely substituted molecules is the first and most important step. The basic concept of diversity-oriented synthesis (DOS) involves short reaction sequences, a strong focus on bond construction, and functional group compatibility [1–3]. Reactions that involve multiple bond formation, such as multicomponent reactions [4–9] and tandem reactions [10–16], are very useful in this context.

**Scheme 1:**
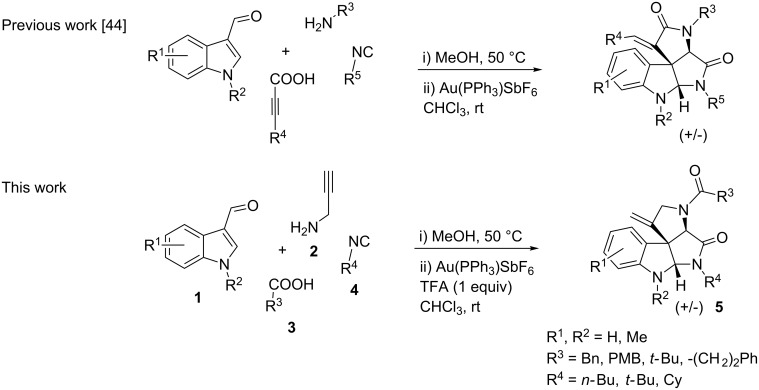
Gold-catalyzed approaches towards spiroindolines.

As an efficient activator of alkynes, gold has recently attracted a lot of attention [17–36]. Many tandem approaches have been reported which utilize this coinage metal for the construction of variously substituted complex molecules [37–43]. We have recently reported a post-Ugi gold-catalyzed intramolecular domino cyclization sequence which produces spiroindolines (Scheme 1) [44]. The first step in this sequence is an Ugi four-component reaction (Ugi-4CR) [4–5] with 2-alkynoic acid as an alkyne source. The second step is a cationic gold-catalyzed intramolecular hydroarylation tandem cyclization to produce spiroindolines with complete diastereoselectivity. This synthetic sequence is atom economic and mild conditions are applied to generate a very complex molecular structure from readily available starting materials. Based on this work and our continuous interest in transition metal catalysis [45–54], multicomponent reactions [55–57] and the chemistry of the indole core [58–60], we herein report a post-Ugi gold-catalyzed intramolecular domino cyclization sequence for the synthesis of spiroindolines with propargylamines as an alkyne source (Scheme 1).

## Results and Discussion

The use of benzoic acid as an acid component in the Ugi-4CR did not produce the Ugi-adduct in good yield even after a prolonged reaction time. Therefore, we switched to phenylacetic acid. The Ugi-4CR of indole-3-carboxaldehyde (**1a**), propargylamine (**2a**), phenylacetic acid (**3a**) and *tert*-butylisonitrile (**4a**) in methanol at 50 °C gave the Ugi-adduct **5a** with an excellent yield of 94%. With compound **5a** in hand we were keen to apply the previously developed conditions for intramolecular hydroarylation [44]. Reaction of **5a** with 5 mol % of Au(PPh_3_)SbF_6_ in chloroform at room temperature produced the desired spiroindoline **6a** in a moderate yield of 55% along with some unidentified byproducts (Table 1, entry 1). The use of a protic acid with a gold catalyst is known in the literature [61–64]. To our delight, when the above reaction was carried out with 1 equivalent of trifluoroacetic acid (TFA) the yield was improved to 81% (Table 1, entry 2). Apart from being a good proton source TFA might be working as a coligand.

**Table 1:** Optimization for the intramolecular hydroarylation.^a^

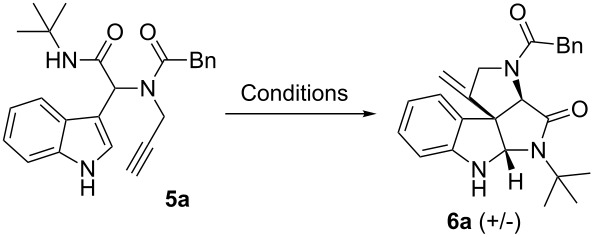				

Entry	Catalyst (mol %)	Acid (1 equiv)	Time h	% Yield^b^

1	Au(PPh_3_)SbF_6_ (5)	—	2	55^c^
**2**	**Au(PPh_3_)SbF_6_ (5)**	**TFA**	**2**	**81**
3	PtCl_2_ (5)	—	10	—^d^
4	PtCl_2_ (5)	TFA	10	—^d^
5	—	TFA	10	—^d^
6	Au(PPh_3_)SbF_6_ (5)	PTSA	2	70

^a^All the reactions were run on 0.1 mmol scale of **5a** with chloroform (2 mL) as a solvent at rt. ^b^Isolated yields. ^c^Unidentified byproducts were formed. ^d^No conversion.

Experiments with PtCl_2_ as a catalyst did not show any conversion and the starting material was recovered quantitatively (Table 1, entries 3 and 4). In absence of the gold catalyst no product could be observed (Table 1, entry 5). The application of *p*-toluenesulfonic acid (PTSA) instead of TFA did not improve the outcome (Table 1, entry 6).

Having the optimized conditions in hand (Table 1, entry 2), various Ugi-adducts **5b–q** were synthesized and subjected to this hydroarylation domino cyclization sequence (Table 2). Different substituents are well-tolerated and the sprioindolines were obtained in good to excellent yields. A methyl substituent on the indole nitrogen did not hamper the domino cyclization (Table 2, entries 4, 6, 11, 12, 14, 15). Substituents like *tert-*butyl, cyclohexyl and *n-*butyl on the isonitrile are well-tolerated for the domino cyclization on the second position of the indole (Table 2, entries 1–16). Regarding the substituents coming from the acid part, *tert-*butyl gave a decreased yield probably due to steric hindrance (Table 2, entry 5). It is noteworthy that the gold-catalyzed intramolecular hydroarylation exclusively gives the *exo*-*dig* product in all cases and with complete diastereoselectivity.

**Table 2:** Scope and limitations of intramolecular domino cyclization.^a^

Entry	Ugi adduct **5**	Spiroindolines **6** (+/−)	Entry	Ugi adduct **5**	Spiroindolines **6** (+/−)

1	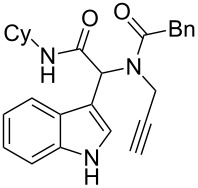 **5b**, 87%	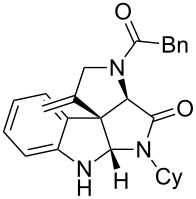 **6b**, 70%	9	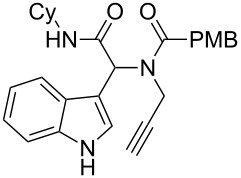 **5j**, 89%	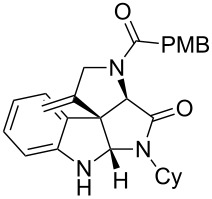 **6j**, 75%
2	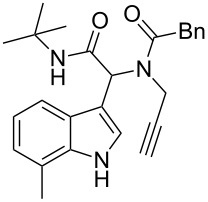 **5c**, 86%	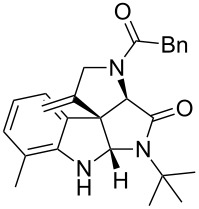 **6c**, 60%	10	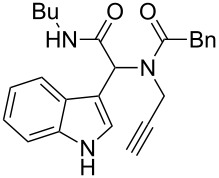 **5k**, 63%	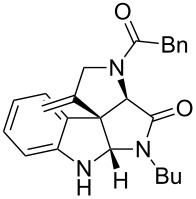 **6k**, 80%
3	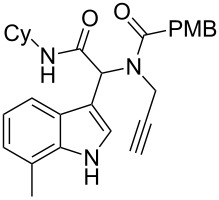 **5d**, 69%	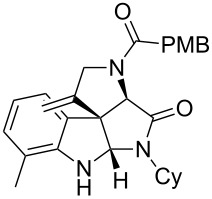 **6d**, 76%	11	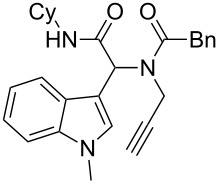 **5l**, 77%	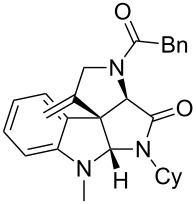 **6l**, 74%
4	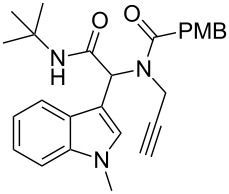 **5e**, 72%	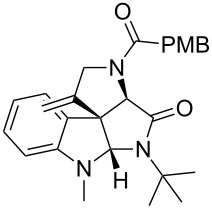 **6e**, 66%	12	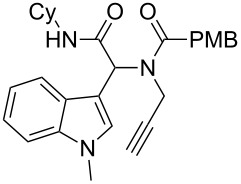 **5m**, 74%	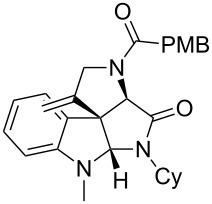 **6m**, 68%
5	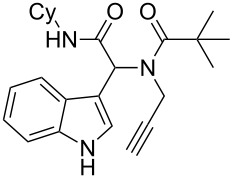 **5f**, 68%	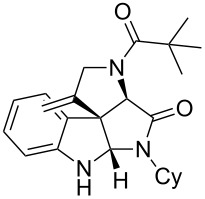 **6f**, 40%	13	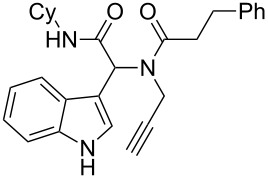 **5n**, 65%	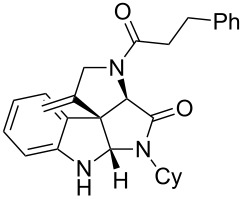 **6n**, 72%
6	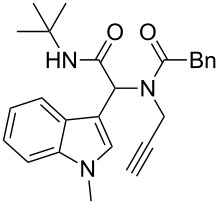 **5g**, 77%	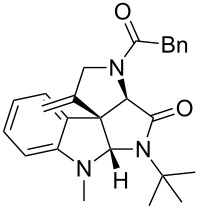 **6g**, 71%	14	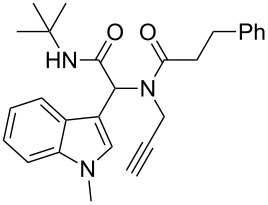 **5o**, 68%	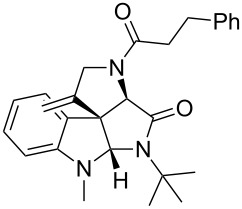 **6o**, 60%
7	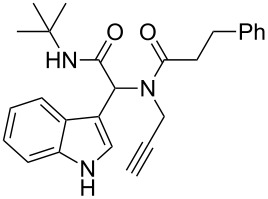 **5h**, 79%	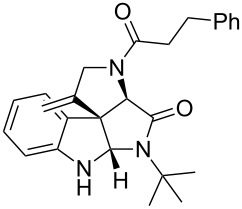 **6h**, 83%	15	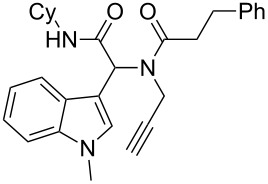 **5p**, 58%	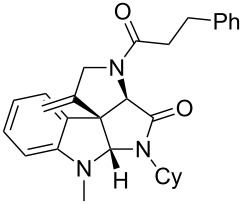 **6p**, 84%
8	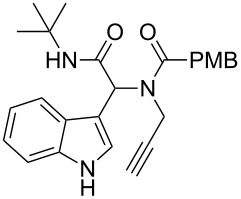 **5i**, 67%	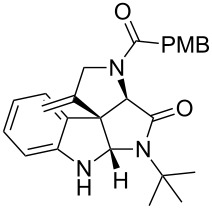 **6i**, 69%	16	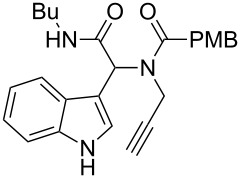 **5q**, 59%	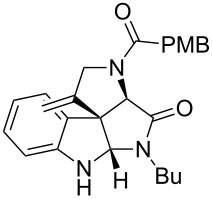 **6q**, 69%

^a^All the reaction were run on a 0.2 mmol scale of **5** in a screw capped vial employing the optimal conditions of Table 1. Cy = cyclohexyl, Bn = benzyl, PMB = *p*-methoxybenzyl, Bu = *n*-butyl.

**Scheme 2:**
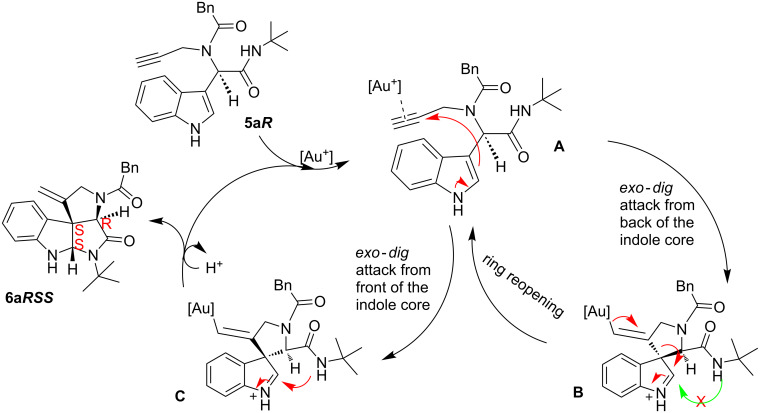
Plausible mechanism for the domino sequence.

A plausible mechanism [30,44] is shown in Scheme 2 with only the *R*-isomer of the Ugi-adduct **5a** to simplify the discussion. The cationic gold coordinates with the terminal alkyne which becomes activated for a nucleophilic attack. This can occur from both sides of the indole core. When the attack occurs from the back side of the indole core, spiro intermediate **B** will be formed. However, in this spiro intermediate the intramolecular trapping of the imminium ion by the amidic NH is sterically impossible and thus the intermediate reopens to intermediate **A**. If the attack takes place from the front side of the indole core, intermediate **C** is formed and trapping is possible. After deprotonation and protodeauration the desired spiroindoline **6a** is formed with the stereochemistry of two new stereocenters *S*.

## Conclusion

In conclusion we have developed a diversity-oriented post-Ugi gold-catalyzed intramolecular hydroarylation domino cyclization sequence for the diastereoselective synthesis of spiroindolines. The mild reaction conditions and short synthetic sequence are the merits of this method. The flexibility given by the multicomponent reaction assures the generation of diversity.

## Experimental

### General procedure for the synthesis of spiroindolines **6a**–**q**

To a screw capped vial Au(PPh_3_)Cl (5 mol %) and AgSbF_6_ (5 mol %) were loaded along with chloroform (2 mL). Ugi product **5** (0.2 mmol) was added followed by TFA (1 equiv), and the reaction mixture was stirred at rt. After completion, the reaction mixture was partitioned between EtOAc (100 mL) and 2 N K_2_CO_3_ solution (2 × 50 mL). The organic layer was washed with brine (50 mL), dried over magnesium sulfate, and evaporated under reduced pressure. The obtained residue was purified by silica gel column chromatography (10% diethyl ether in dichloromethane) to afford compound **6a**–**q**.

## Supporting Information

File 1 Experimental section.
