# Supplementary material for: Post-Ugi gold-catalyzed diastereoselective domino cyclization for the synthesis of diversely substituted spiroindolines
Source: Beilstein J Org Chem. 2013 Oct 14;9:2097–102. doi: 10.3762/bjoc.9.246 (PMC3817510; doi:10.3762/bjoc.9.246)

Supporting Information

for

Post-Ugi gold-catalyzed diastereoselective domino cyclization for the synthesis of diversely substituted spiroindolines

Amit Kumar[1,2], Dipak D. Vachhani[1], Sachin G. Modha*[1,3], Sunil K. Sharma[2], Virinder S. Parmar[2] and Erik V. Van der Eycken*[1]

Address: [1]Laboratory for Organic & Microwave-Assisted Chemistry (LOMAC), Department of Chemistry, University of Leuven (KU Leuven), Celestijnenlaan 200F, 3001, Leuven, Belgium, [2]Bioorganic Laboratory, Department of Chemistry, University of Delhi, Delhi-110 007, India and [3]Current address: Chemistry Building-4.20b, School of Chemistry, The University of Manchester, Manchester M13 9PL, UK

Email: Sachin G. Modha - sachinmodha@gmail.com, Erik V. Van der Eycken - erik.vandereycken@chem.kuleuven.be

* Corresponding author

Experimental section

Table of contents

General experimental procedures and data...S2–S19

Copies of ^{1}H and ^{13}C NMR spectra...S20–S53

Materials:

All the starting materials, reagents and catalysts were purchased from Aldrich or Acros and used as such. For thin layer chromatography, analytical TLC plates (Alugram SIL G/UV254 and 70–230 mesh silica gel (E. M. Merck) were used). Column chromatography was performed using silica gel (Merck, 60–120 mesh size). Anhydrous solvents were purchased from Acros Organics and stored over molecular sieves. The chromatographic solvents used for isolation/purification of compounds were distilled prior to use. The chromatographic solvents are mentioned as volume:volume ratios. Reactions were typically run in oven-dried screw-cap vial under inert atmosphere.

Apparatus:

^{1}H (300 MHz) and ^{13}C (75.5 MHz) NMR spectra were recorded on a 400 MHz & 300 MHz instrument using $CDCl_3$ and DMSO-d_6 as a solvent. The ^{1}H and ^{13}C chemical shifts are reported in parts per million relative to tetramethylsilane using the residual solvent signal as the internal reference. The following abbreviations were used to designate chemical shift multiplicities: s = singlet, bs = broad singlet, d = doublet, dd = double doublet, t = triplet, m = multiplet. The ^{13}C NMR spectra are proton decoupled. The melting points were determined on a digital Barnsted Electrothermal 9200 apparatus and are uncorrected. Mass spectra were recorded by using a Kratos MS50TC and a Kratos Mach III system. The ion source temperature was 150–250 °C, as required. High resolution EI-mass spectra were performed with a resolution of 10,000. The low-resolution spectra were obtained with a HP5989A MS instrument. The low resolution ESI–MS were obtained with a Thermo Scientific instrument.

General procedure for synthesis of Ugi products 5a–q.

To a solution of substituted indole-3-carbaldehyde **1** (1.5 mmol) in methanol (5 mL) were added successively Na_2SO_4 (0.3 g), propargylamine **2** (1.1 equiv), acid **3** (1.1 equiv) and isonitrile **4** (1.1 equiv) in a 25 mL RBF equipped with a magnetic stir bar. The reaction mixture was stirred at 50 °C for 24 h. After completion of the reaction, the mixture was diluted with EtOAc (100 mL) and was extracted with water (50 mL). The organic layer was washed with brine (50 mL), dried over magnesium sulfate and evaporated under reduced pressure to obtain residue which was subjected to silica gel column chromatography (80% EtOAc in heptane) to afford the desired product **5a–q** as solid.

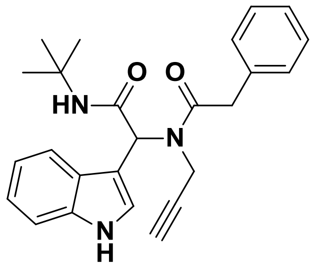

N-(*tert*-butyl)-2-(1*H*-indol-3-yl)-2-(2-phenyl-*N*-(prop-2-yn-1-yl)acetamido)acetamide (**5a**)

White solid, Yield 94%, Melting point: 62–64 °C.

^{1}H NMR (300 MHz, CDCl$_3$) δ 8.25 (bs, 1H), 7.62 (d, *J* = 2.41 Hz, 1H), 7.44–7.31 (m, 7H), 7.20 (t, *J* = 8.05 Hz, 1H), 7.07 (t, *J* = 7.50 Hz, 1H), 6.45 (s, 1H), 5.98 (bs, 1H), 4.05 (bs, 2H), 3.96 (s, 2H), 1.91 (s, 1H), 1.35 (s, 9H).

^{13}C NMR (75 MHz, CDCl$_3$) δ 172.1, 168.8, 135.8, 134.5, 129.0, 128.6, 127.0, 126.9, 125.9(2), 122.6, 120.1, 119.0, 111.1, 79.5, 71.7, 53.9, 51.5, 41.1, 34.2, 28.6.

HRMS calculated for $C_{25}H_{27}N_3O_2$ 401.2103, found 401.2100.

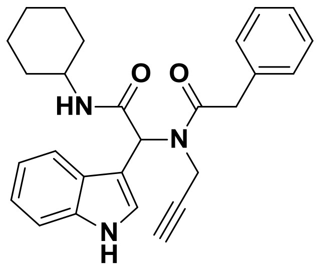

N-cyclohexyl-2-(1*H*-indol-3-yl)-2-(2-phenyl-*N*-(prop-2-yn-1-yl)acetamido)acetamide (**5b**)

Orange solid, Yield 87%, Melting point: 69–71 °C.

^{1}H NMR (300 MHz, CDCl$_3$) δ 8.41 (bs, 1H), 7.59 (s, 1H), 7.43–7.31 (m, 7H), 7.19 (t, *J* = 7.52 Hz, 1H), 7.06 (t, *J* = 7.33 Hz, 1H), 6.53 (s, 1H), 6.05 (d, *J* = 8.16 Hz, 1H), 4.03 (s, 2H), 3.95 (s, 2H), 3.82–3.79 (m, 1H), 1.95–1.87 (m, 3H), 1.66–1.56 (m, 2H), 1.40–1.25 (m, 3H), 1.15–1.09 (m, 3H).

^{13}C NMR (75 MHz, CDCl$_3$) δ 172.2, 168.6, 135.9, 134.5, 129.0, 128.7, 127.0, 126.9, 126.1, 122.6, 120.1, 118.9, 111.3, 109.0, 79.4, 71.9, 53.7, 48.4, 41.1, 34.3, 32.8, 32.7, 25.4, 24.7.

HRMS calculated for $C_{27}H_{29}N_3O_2$ 427.2260, found 427.2251.

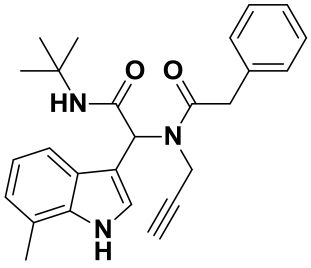

N-(*tert*-butyl)-2-(7-methyl-1*H*-indol-3-yl)-2-(2-phenyl-*N*-(prop-2-yn-1-yl)acetamido)acetamide (**5c**)

Offwhite solid, Yield 86%, Melting point: 68–70 °C.

^{1}H NMR (300 MHz, CDCl$_3$) δ 8.32 (bs, 1H), 7.60 (d, *J* = 2.29 Hz, 1H), 7.36–7.27 (m, 6H), 6.99–6.98 (m, 2H), 6.42 (s, 1H), 6.00 (bs, 1H), 4.04 (d, *J* = 2.17 Hz, 2H), 3.96 (s, 2H), 2.47 (s, 3H), 1.96 (t, *J* = 2.37 Hz, 1H), 1.35 (s, 9H).

^{13}C NMR (75 MHz, CDCl$_3$) δ 172.1, 168.9, 135.5, 134.5, 129.0, 128.6, 126.9, 126.5, 125.8, 123.0, 120.5, 120.2, 116.5, 109.4, 79.6, 71.9, 54.4, 51.4, 41.1, 34.3, 28.5, 16.5.

HRMS calculated for $C_{26}H_{29}N_3O_2$ 415.2260, found 415.2250.

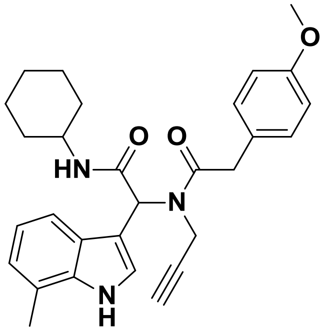

N-cyclohexyl-2-(2-(4-methoxyphenyl)-*N*-(prop-2-yn-1-yl)acetamido)-2-(7-methyl-1*H*-indol-3-yl)acetamide (**5d**)

White solid, Yield 69%, Melting point: 78–80 °C.

^{1}H NMR (300 MHz, CDCl$_3$) δ 8.28 (bs, 1H), 7.62 (d, *J* = 2.15 Hz, 1H), 7.24–7.21 (m, 3H), 7.00–6.98 (m, 2H), 6.85 (d, *J* = 8.50 Hz, 2H), 6.49 (s, 1H), 6.04 (d, *J* = 8.35 Hz, 1H), 4.02 (d, *J* = 1.98 Hz, 2H), 3.89 (s, 2H), 3.79 (m, 4H), 2.48 (s, 3H), 1.99 (bs, 1H), 1.91–1.87 (m, 2H), 1.69–1.64 (m, 3H), 1.36–1.28 (m, 2H), 1.15–1.12 (m, 3H).

^{13}C NMR (75 MHz, CDCl$_3$) δ 172.5, 168.7, 158.6, 135.6, 130.1, 130.0, 126.5, 125.9, 123.0, 120.5, 120.2, 116.5, 114.1, 109.3, 79.6, 71.9, 55.2, 54.1, 48.4, 40.3, 34.3, 32.8, 32.6, 25.5, 24.8, 16.6.

HRMS calculated for $C_{29}H_{33}N_3O_3$ 471.2522, found 471.2533.

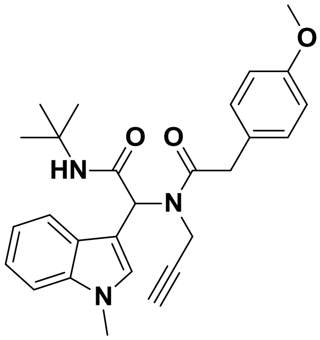

N-(*tert*-butyl)-2-(2-(4-methoxyphenyl)-*N*-(prop-2-yn-1-yl)acetamido)-2-(1-methyl-1*H*-indol-3-yl)acetamide (**5e**)

Offwhite solid, Yield 72%, Melting point: 178–180 °C.

^{1}H NMR (300 MHz, CDCl$_3$) δ 7.51 (s, 1H), 7.38 (d, *J* = 7.91 Hz, 1H), 7.29 (d, *J* = 8.11 Hz, 1H), 7.24–7.19 (m, 3H), 7.05 (t, *J* = 7.37 Hz, 1H), 6.85 (d, *J* = 8.45 Hz, 2H), 6.42 (s, 1H), 6.04 (bs, 1H), 4.01 (d, *J* = 1.94 Hz, 2H), 3.89 (s, 2H), 3.79–3.78 (m, 6H), 1.96 (s, 1H), 1.35 (s, 9H).

^{13}C NMR (75 MHz, CDCl$_3$) δ 172.4, 168.9, 158.6, 136.7, 130.6, 130.0, 127.5, 126.5, 122.1, 119.5, 119.1, 114.1, 109.2, 107.4, 79.6, 71.7, 55.2, 53.9, 51.4, 40.3, 34.0, 32.9, 28.6.

HRMS calculated for $C_{27}H_{31}N_3O_3$ 445.2365, found 445.2361.

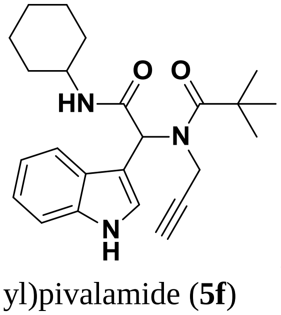

N-(2-(cyclohexylamino)-1-(1*H*-indol-3-yl)-2-oxoethyl)-*N*-(prop-2-yn-1-yl)pivalamide (**5f**)

Orange solid, Yield 68%, Melting point: 73–75 °C.

^{1}H NMR (300 MHz, CDCl$_3$) δ 8.46 (bs, 1H), 7.61 (s, 1H), 7.45–7.37 (m, 2H), 7.20 (t, *J* = 7.68 Hz, 1H), 7.11 (t, *J* = 7.50 Hz, 1H), 6.34–6.29 (m, 2H), 4.27 (d, *J* = 18.38 Hz, 1H), 4.02 (d, *J* = 18.38 Hz, 1H), 3.89–3.79 (m, 1H), 1.96–1.88 (m, 3H), 1.69–1.63 (m, 3H), 1.38–1.32 (m, 11H), 1.21–1.13 (m, 3H).

^{13}C NMR (75 MHz, CDCl$_3$) δ 178.5, 169.3, 136.0, 126.3, 123.9, 122.3, 120.0, 118.4, 111.5, 108.7, 80.2, 72.1, 48.4, 39.6, 35.7, 32.8, 32.7, 30.9, 28.6, 25.4, 24.7, 24.6.

HRMS calculated for $C_{24}H_{31}N_3O_2$ 393.2416, found 393.2402.

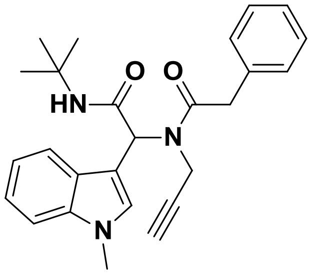

N-(*tert*-butyl)-2-(1-methyl-1*H*-indol-3-yl)-2-(2-phenyl-*N*-(prop-2-yn-1-yl)acetamido)acetamide (**5g**)

White solid, Yield 77%, Melting point: 134–136 °C.

^{1}H NMR (300 MHz, CDCl$_3$) δ 7.51 (s, 1H), 7.38 (d, *J* = 7.92 Hz, 1H), 7.32–7.24 (m, 6H), 7.20 (d, *J* = 7.68 Hz, 1H), 7.05 (t, *J* = 7.53 Hz, 1H), 6.43 (s, 1H), 6.04 (bs, 1H), 4.02 (d, *J* = 2.17 Hz, 2H), 3.95 (s, 2H), 3.78 (s, 3H), 1.95 (bs, 1H), 1.35 (s, 9H).

^{13}C NMR (75 MHz, CDCl$_3$) δ 172.1, 168.8, 136.7, 134.5, 130.6, 129.0, 128.6, 127.5, 126.9, 122.1, 119.6, 119.1, 109.2, 107.4, 79.5, 71.8, 53.9, 51.4, 41.2, 34.1, 32.9, 28.6.

HRMS calculated for $C_{26}H_{29}N_3O_2$ 415.2260, found 415.2263.

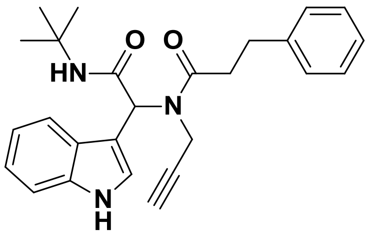

N-(2-(*tert*-butylamino)-1-(1*H*-indol-3-yl)-2-oxoethyl)-3-phenyl-*N*-(prop-2-yn-1-yl)propanamide (**5h**)

White solid, Yield 79%, Melting point: 172–174 °C.

^{1}H NMR (300 MHz, CDCl$_3$) δ 8.33 (bs, 1H), 7.56 (d, *J* = 2.09 Hz, 1H), 7.41–7.35(m, 2H), 7.28–7.21 (m, 6H), 7.08 (t, *J* = 7.41 Hz, 1H), 6.46 (s, 1H), 5.91 (bs, 1H), 4.03 (d, *J* = 2.19 Hz, 2H), 3.09 (t, *J* = 7.94, 2H), 2.87 (t, *J* = 7.94 Hz, 2H), 1.83 (bs, 1H), 1.35 (s, 9H).

^{13}C NMR (75 MHz, CDCl$_3$) δ 173.2, 169.2, 141.0, 136.0, 128.4, 126.9, 126.0, 125.9, 122.5, 120.0, 118.8, 111.4, 109.1, 79.5, 71.7, 54.1, 51.5, 35.3, 34.2, 31.0, 28.5.

HRMS calculated for $C_{26}H_{29}N_3O_2$ 415.2260, found 415.2250.

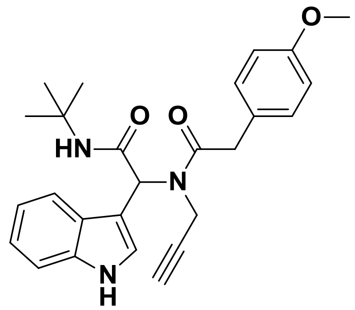

N-(*tert*-butyl)-2-(1*H*-indol-3-yl)-2-(2-(4-methoxyphenyl)-*N*-(prop-2-yn-1-yl)acetamido)acetamide (**5i**)

Offwhite solid, Yield 67%, Melting point: 148–150 °C.

^{1}H NMR (300 MHz, CDCl$_3$) δ 8.40 (bs, 1H), 7.59 (s, 1H), 7.41 (d, *J* = 7.93 Hz, 1H), 7.35 (d, *J* = 8.16 Hz, 1H), 7.24–7.16 (m, 3H), 7.06 (t, *J* = 7.48 Hz, 1H), 6.85 (d, *J* = 8.57 Hz, 2H), 6.44 (s, 1H), 6.00 (bs, 1H), 4.05 (bs, 2H), 3.89 (s, 2H), 3.79 (s, 3H), 1.92 (bs, 1H), 1.35 (s, 9H).

^{13}C NMR (75 MHz, CDCl$_3$) δ 172.4, 168.9, 158.6, 135.9, 130.0, 127.0, 126.5, 125.9, 122.6, 120.0, 119.0, 114.1, 111.2, 109.3, 79.6, 71.7, 55.2, 54.0, 51.5, 40.2, 34.2, 28.6.

HRMS calculated for $C_{26}H_{29}N_3O_3$ 431.2209, found 431.2209.

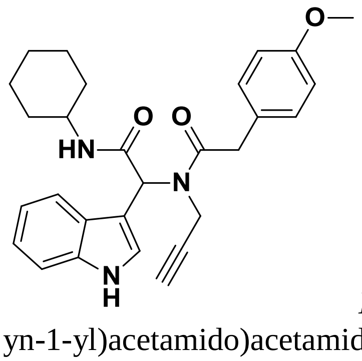

N-cyclohexyl-2-(1*H*-indol-3-yl)-2-(2-(4-methoxyphenyl)-*N*-(prop-2-yn-1-yl)acetamido)acetamide (**5j**)

Orange solid, Yield 89%, Melting point: 153–155 °C.

^{1}H NMR (300 MHz, CDCl$_3$) δ 8.42 (bs, 1H), 7.59 (s, 1H), 7.41 (d, *J* = 7.96 Hz, 1H), 7.35 (d, *J* = 8.12 Hz, 1H), 7.24–7.16 (m, 3H), 7.06 (t, *J* = 7.47 Hz, 1H), 6.85 (d, *J* = 8.55 Hz, 2H), 6.52 (s, 1H), 6.06 (d, *J* = 7.89 Hz, 1H), 4.03 (bs, 2H), 3.88 (s, 2H), 3.79 (m, 4H), 1.95–1.87 (m, 3H), 1.66–1.56 (m, 2H), 1.35–1.26 (m, 3H), 1.15–1.12 (m, 3H).

^{13}C NMR (75 MHz, CDCl$_3$) δ 172.5, 168.8, 158.6, 136.0, 130.1, 126.9, 126.5, 126.1, 122.5, 119.9, 118.8, 114.1, 111.4, 108.8, 79.5, 71.8, 55.2, 53.9, 48.4, 40.2, 34.3, 32.7, 32.6, 25.4, 24.7.

HRMS calculated for $C_{28}H_{31}N_3O_3$ 457.2365, found 457.2394.

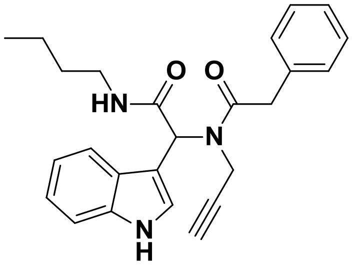

N-butyl-2-(1*H*-indol-3-yl)-2-(2-phenyl-*N*-(prop-2-yn-1-yl)acetamido)acetamide **(5k)**

Offwhite solid, Yield 63%, Melting point: 90–92 °C.

^{1}H NMR (300 MHz, CDCl$_3$) δ 8.37 (bs, 1H), 7.62 (s, 1H), 7.42–7.19 (m, 8H), 7.06 (t, *J* = 7.34 Hz, 1H), 6.53 (s, 1H), 6.18 (bs, 1H), 4.05 (s, 2H), 3.95 (s, 2H), 3.28–3.26 (m, 2H), 1.95 (s, 1H), 1.47–1.44 (m, 2H), 1.32–1.29 (m, 2H), 0.89 (t, *J* = 6.93 Hz, 3H).

^{13}C NMR (75 MHz, CDCl$_3$) δ 172.2, 169.5, 135.9, 134.4, 129.1, 128.6, 127.0, 126.9, 126.1, 122.6, 120.1, 118.9, 111.2, 108.9, 79.3, 71.8, 53.5, 41.1, 39.3, 34.3, 31.4, 20.0, 13.7.

HRMS calculated for $C_{25}H_{27}N_3O_2$ 401.2103, found 401.2101.

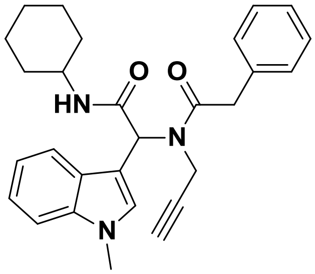

N-cyclohexyl-2-(1-methyl-1*H*-indol-3-yl)-2-(2-phenyl-*N*-(prop-2-yn-1-yl)acetamido)acetamide (**5l**)

White solid, Yield 77%, Melting point: 183–185 °C.

^{1}H NMR (300 MHz, CDCl$_3$) δ 7.50 (s, 1H), 7.40–7.19 (m, 8H), 7.05 (t, *J* = 7.46 Hz, 1H), 6.50 (s, 1H), 6.08 (d, *J* = 8.11 Hz, 1H), 4.02 (s, 2H), 3.95 (s, 2H), 3.78 (s, 3H), 1.98–1.88 (m, 3H), 1.64–1.57 (m, 2H), 1.41–1.25 (m, 3H), 1.16–1.14 (m, 3H).

^{13}C NMR (75 MHz, CDCl$_3$) δ 172.1, 168.6, 136.7, 134.5, 130.6, 129.0, 128.6, 127.5, 126.9, 122.1, 119.6, 119.0, 109.2, 107.3, 79.5, 71.8, 53.5, 48.3, 41.2, 34.2, 32.9, 32.8, 32.7, 25.5, 24.7.

HRMS calculated for $C_{28}H_{31}N_3O_2$ 441.2416, found 441.2411.

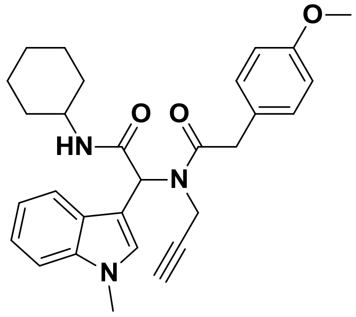

N-cyclohexyl-2-(2-(4-methoxyphenyl)-*N*-(prop-2-yn-1-yl)acetamido)-2-(1-methyl-1*H*-indol-3-yl)acetamide (**5m**)

White solid, Yield 74%, Melting point: 119–121 °C.

^{1}H NMR (300 MHz, CDCl$_3$) δ 7.50 (s, 1H), 7.38 (d, *J* = 7.96 Hz, 1H), 7.29 (d, *J* = 8.20 Hz, 1H), 7.24–7.21 (m, 3H), 7.05 (t, *J* = 7.33 Hz, 1H), 6.86 (d, *J* = 8.51 Hz, 2H), 6.49 (s, 1H), 6.08 (d, *J* = 7.98 Hz, 1H), 4.01 (s, 2H), 3.88 (s, 2H), 3.79–3.78 (m, 7H), 1.98 (bs, 1H), 1.92–1.88 (m, 2H), 1.70–1.66 (m, 3H), 1.37–1.29 (m, 3H), 1.16–1.14 (m, 3H).

^{13}C NMR (75 MHz, CDCl$_3$) δ 172.4, 168.6, 158.6, 136.7, 130.6, 130.0, 127.5, 126.5, 122.1, 119.6, 119.1, 114.1, 109.2, 107.3, 79.5, 71.8, 55.2, 53.5, 48.3, 40.3, 34.1, 32.9, 32.8, 32.7, 25.5, 24.7.

HRMS calculated for $C_{29}H_{33}N_3O_3$ 471.2522, found 471.2517.

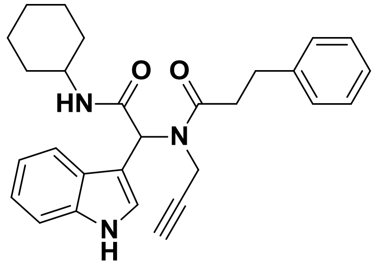

N-(2-(cyclohexylamino)-1-(1*H*-indol-3-yl)-2-oxoethyl)-3-phenyl-*N*-(prop-2-yn-1-yl)propanamide (**5n**)

Offwhite solid, Yield 65%, Melting point: 62–64 °C.

^{1}H NMR (300 MHz, CDCl$_3$) δ 8.32 (bs, 1H), 7.59 (d, *J* = 1.90 Hz, 1H), 7.40–7.36 (m, 2H), 7.28–7.18 (m, 6H), 7.08 (t, *J* = 7.44 Hz, 1H), 6.53 (s, 1H), 6.00 (d, *J* = 7.93 Hz, 1H), 4.02 (d, *J* = 1.98 Hz, 2H), 3.82–3.79 (m, 1H), 3.09 (t, *J* = 7.60 Hz, 2H), 2.86 (t, *J* = 7.46 Hz, 2H), 1.92–1.88 (m, 3H), 1.70–1.65 (m, 3H), 1.37–1.33 (m, 3H), 1.16–1.12 (m, 2H).

^{13}C NMR (75 MHz, CDCl$_3$) δ 173.3, 168.8, 141.1, 135.9, 128.5, 128.4, 126.9, 126.1, 125.9, 122.6, 120.1, 118.9, 111.2, 109.2, 79.4, 77.2, 71.6, 53.4, 48.4, 35.4, 34.1, 32.8, 32.6, 31.1, 25.4, 24.7.

HRMS calculated for $C_{28}H_{31}N_3O_2$ 441.2416, found 441.2415.

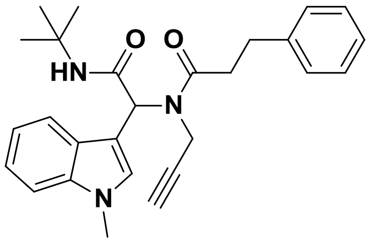

N-(2-(*tert*-butylamino)-1-(1-methyl-1*H*-indol-3-yl)-2-oxoethyl)-3-phenyl-*N*-(prop-2-yn-1-yl)propanamide (**5o**)

White solid, Yield 68%, Melting point: 112–114 °C.

^{1}H NMR (300 MHz, CDCl$_3$) δ 7.47 (s, 1H), 7.35 (d, *J* = 7.88 Hz, 1H), 7.31–7.20 (m, 7H), 7.07 (t, *J* = 7.40 Hz, 1H), 6.43 (s, 1H), 5.98 (bs 1H), 4.01 (bs, 2H), 3.79 (s, 3H), 3.12–3.07 (m, 2H), 2.89–2.86 (m, 2H), 1.88 (s, 1H), 1.35 (s, 9H).

^{13}C NMR (75 MHz, CDCl$_3$) δ 173.1, 169.0, 141.1, 136.7, 130.4, 128.4(2), 127.5, 126.0, 122.1, 119.7, 119.0, 109.2, 107.6, 79.5, 71.5, 53.6, 51.4, 35.3, 34.0, 32.9, 31.1, 28.6.

HRMS calculated for $C_{27}H_{31}N_3O_2$ 429.2416, found 429.2426.

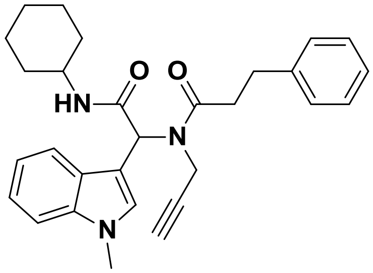

N-(2-(cyclohexylamino)-1-(1-methyl-1*H*-indol-3-yl)-2-oxoethyl)-3-phenyl-*N*-(prop-2-yn-1-yl)propanamide (**5p**)

Offwhite solid, Yield 58%, Melting point: 157–159 °C.

^{1}H NMR (300 MHz, CDCl$_3$) δ 7.47 (s, 1H), 7.36 (d, *J* = 8.01 Hz, 1H), 7.31–7.21 (m, 7H), 7.07 (t, *J* = 7.26 Hz, 1H), 6.49 (s, 1H), 6.03 (d, *J* = 7.79 Hz, 1H), 4.00 (bs, 2H), 3.78 (m, 4H), 3.11–3.06 (m, 2H), 2.89–2.87 (m, 2H), 1.92–1.89 (m, 3H), 1.70–1.66 (m, 3H), 1.37–1.29 (m, 2H), 1.16–1.10 (m, 3H).

^{13}C NMR (75 MHz, CDCl$_3$) δ 173.2, 168.7, 141.1, 136.8, 130.5, 128.4, 127.5, 126.1, 122.1, 119.7, 119.0, 109.3, 107.5, 79.5, 71.6, 53.3, 48.4, 35.4, 34.1, 32.9, 32.8, 32.7, 31.1, 25.5, 24.7.

HRMS calculated for $C_{29}H_{33}N_3O_2$ 455.2573, found 455.2568.

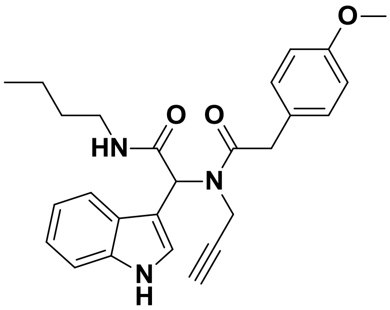

N-butyl-2-(1*H*-indol-3-yl)-2-(2-(4-methoxyphenyl)-*N*-(prop-2-yn-1-yl)acetamido)acetamide (**5q**)

Yellow solid, Yield 59%, Melting point: 105–107 °C.

^{1}H NMR (300 MHz, $CDCl_3$) δ 8.46 (bs, 1H), 7.60 (s, 1H), 7.41–7.34 (m, 2H), 7.25–7.16 (m, 3H), 7.06 (t, *J* = 7.40 Hz, 1H), 6.85 (d, *J* = 8.18 Hz, 2H), 6.52 (s, 1H), 6.20 (bs, 1H), 4.04 (s, 2H), 3.88 (s, 2H), 3.79 (s, 3H), 3.28–3.26 (m, 2H), 1.96 (s, 1H), 1.46–1.44 (m, 2H), 1.31–1.26 (m, 2H), 0.89 (t, *J* = 7.06 Hz, 3H).

^{13}C NMR (75 MHz, $CDCl_3$) δ 172.5, 169.6, 158.6, 135.9, 130.1, 126.9, 126.4, 126.2, 122.5, 120.0, 118.8, 114.1, 111.3, 108.8, 79.4, 71.8, 55.2, 53.7, 40.2, 39.3, 34.3, 31.3, 20.0, 13.7.

HRMS calculated for $C_{26}H_{29}N_3O_3$ 431.2209, found 431.2202.

General procedure for synthesis of spiroindolines 6a–q.

To a screw capped vial $Au(PPh_3)Cl$ (5 mol %) and $AgSbF_6$ (5 mol %) were loaded along with chloroform (2 mL). Ugi product **5** (0.2 mmol) was added followed by TFA (1 equiv) and reaction mixture was stirred at rt until completion. After completion, reaction mixture was partitioned between EtOAc (100 mL) and and 2 N K_2CO_3 solution (2 × 50 mL). Organic layer was washed with brine (50 mL), dried over magnesium sulfate and evaporated under reduced pressure. The residue obtained was purified by silica gel column chromatography (10% diethyl ether in dichloromethane) to afford compound **6a**–**q**.

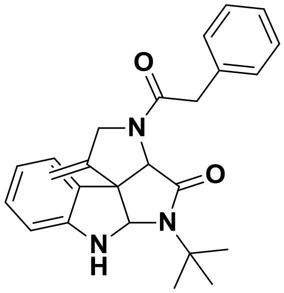

5-(*tert*-butyl)-1-methylene-3-(2-phenylacetyl)-1,2,3,3a,5a,6-hexahydropyrrolo[3',2':3,4]pyrrolo[2,3-*b*]indol-4(5*H*)-one (**6a**)

White Solid, Yield 81%, Melting point: 72–74 °C.

^{1}H NMR (300 MHz, CDCl$_3$) δ 7.26–7.16 (m, 5H), 7.10 (t, 1H), 6.75–6.63 (m, 3H), 5.48 (d, *J* = 4.5 Hz, 1H), 5.33 (s, 1H), 4.94 (s, 1H), 4.80 (d, *J* = 16.56 Hz, 1H), 4.55 (s, 1H), 4.38 (d, 1H, 4.35), 4.06–4.00 (m, 3H), 1.50 (s, 9H).

^{13}C NMR (75 MHz, CDCl$_3$) δ 171.0, 170.8, 149.3, 148.9, 135.0, 130.4, 129.2, 129.0, 128.5, 126.6, 123.8, 120.6, 111.6, 110.1, 83.6, 69.5, 60.1, 55.3, 50.5, 40.8, 28.0

HRMS Calculated for $C_{25}H_{27}N_3O_2$ 401.2103, found 401.2111.

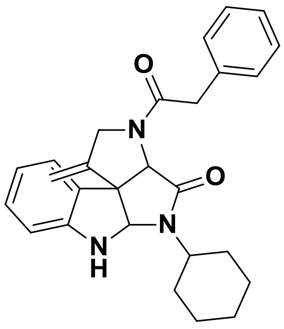

5-cyclohexyl-1-methylene-3-(2-phenylacetyl)-1,2,3,3a,5a,6-hexahydropyrrolo[3',2':3,4]pyrrolo[2,3-*b*]indol-4(5*H*)-one (**6b**)

White Solid, Yield 70%, Melting point: 91–93 °C.

^{1}H NMR (300 MHz, CDCl$_3$) δ 7.32–7.16 (m, 4H), 7.11 (t, *J* = 7.43 Hz, 1H), 6.76–6.69 (m, 2H), 6.64 (d, *J* = 7.92 Hz, 1H), 5.36 (d, *J* = 4.14 Hz, 1H), 5.28 (s, 1H), 4.86–4.76 (m, 2H), 4.61 (s, 1H), 4.45 (d, *J* = 3.39 Hz, 1H), 4.05–3.99 (m, 3H), 3.93–3.79 (m, 1H), 2.02–1.68 (m, 4H), 1.57–1.46 (m, 2H), 1.38–1.25 (m, 2H), 1.21–1.08 (m, 2H).

^{13}C NMR (75 MHz, CDCl$_3$) δ 170.8, 170.1, 148.9, 148.7, 135.0, 130.0, 129.4, 129.1, 128.5, 126.6, 123.9, 120.7, 111.5, 110.3, 81.8, 68.7, 61.3, 52.8, 50.7, 40.8, 32.0, 30.3, 25.7, 25.5, 25.4.

HRMS Calculated for $C_{27}H_{29}N_3O_2$ 427.2260, found 427.2264.

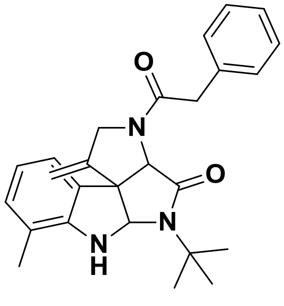

5-(*tert*-butyl)-7-methyl-1-methylene-3-(2-phenylacetyl)-1,2,3,3a,5a,6-hexahydropyrrolo[3',2':3,4]pyrrolo[2,3-*b*]indol-4(5*H*)-one (**6c**)

yellow Solid, Yield 60%, Melting point: 79–81 °C.

¹H NMR (300 MHz, $CDCl_3$) δ 7.35–7.17 (m, 5H), 6.94 (d, *J* = 7.35 Hz, 1H), 6.68 (t, *J* = 7.43 Hz, 1H), 6.55 (d, *J* = 7.44 Hz, 1H), 5.49(d, *J* = 4.53 Hz, 1H), 5.31(s, 1H), 4.92–4.77(m, 2H), 4.54 (s, 1H), 4.14–4.06 (m, 2H), 4.00–3.99 (m, 2H), 2.15 (s, 3H), 1.51 (s, 9H)

¹³C NMR (75 MHz, $CDCl_3$) δ 171.1, 170.8, 149.4, 147.4, 135.0, 130.0, 129.9, 129.1, 128.4, 126.6, 121.2, 120.9, 119.8, 111.5, 83.5, 69.6, 60.5, 55.3, 50.5, 40.8, 28.1, 16.7.

HRMS Calculated for $C_{26}H_{29}N_3O_2$ 415.2260, found 415.2251.

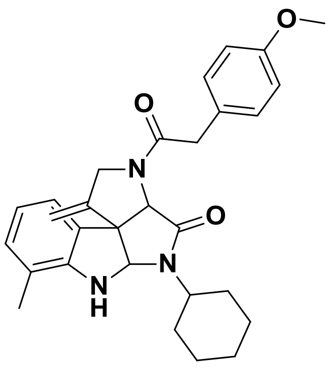

5-cyclohexyl-3-(2-(4-methoxyphenyl)acetyl)-7-methyl-1-methylene-1,2,3,3a,5a,6-hexahydropyrrolo[3',2':3,4]pyrrolo[2,3-*b*]indol-4(5*H*)-one (**6d**)

Yellow solid, Yield 76%, Melting point: 77–79 °C.

¹H NMR (300 MHz, $CDCl_3$) δ 7.18 (d, *J* = 8.45 Hz, 2H), 6.95(d, *J* = 7.51 Hz, 1H), 6.80 (d, *J* = 8.62 Hz, 2H), 6.70 (t, *J* = 7.40 Hz, 1H), 6.57 (d, *J* = 7.46 Hz, 1H), 5.36 (s, 1H), 5.27(s, 1H), 4.85–4.75(m, 2H), 4.61 (s, 1H), 4.16 (bs, 1H), 4.04–3.90 (m, 4H), 3.76 (s, 3H), 2.15 (s, 3H), 1.75–1.34 (m, 10H).

¹³C NMR (75 MHz, $CDCl_3$) δ 171.2, 170.1, 158.4, 148.8, 147.5, 130.2, 130.1, 129.6, 127.1, 121.3, 120.9, 120.0, 114.0, 111.3, 81.7, 68.8, 61.7, 55.2, 52.9, 50.7, 39.9, 32.0, 30.3, 25.7, 25.5, 25.4, 16.7.

HRMS calculated for $C_{29}H_{33}N_3O_3$ 471.2522, found 471.2525.

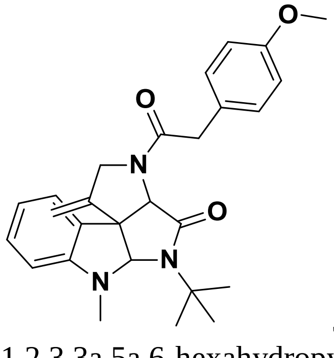

5-(*tert*-butyl)-3-(2-(4-methoxyphenyl)acetyl)-6-methyl-1-methylene-1,2,3,3a,5a,6-hexahydropyrrolo[3',2':3,4]pyrrolo[2,3-*b*]indol-4(5*H*)-one (**6e**)

Brown solid, Yield 66%, Melting point: 50–52 °C.

^{1}H NMR (400 MHz, $CDCl_3$) δ 7.16–7.11 (m, H), 6.76–6.72 (m, 4H), 6.55 (d, *J* = 7.85 Hz, 1H), 5.37 (s, 1H), 5.06–5.05 (m, 2H), 4.76(d, *J* = 17.00 Hz, 1H), 4.50 (s, 1H), 4.04 (d, *J* = 16.94 Hz, 1H), 3.94–3.83 (m, 2H), 3.74 (s, 3H), 2.96 (s, 3H), 1.52 (s, 9H).

^{13}C NMR (100 MHz, $CDCl_3$) δ 172.4, 158.3, 151.2, 147.3, 130.3, 130.1, 129.2, 126.9, 123.5, 122.9, 120.0, 113.9, 111.7, 108.8, 90.3, 69.7, 60.8, 55.5, 55.2, 51.1, 39.9, 36.2, 28.4.

HRMS calculated for $C_{27}H_{31}N_3O_3$ 445.2365, found 445.2379.

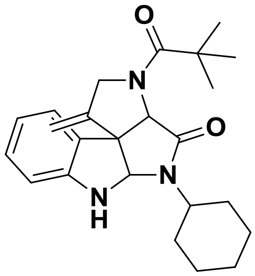

5-cyclohexyl-1-methylene-3-pivaloyl-1,2,3,3a,5a,6-hexahydropyrrolo[3',2':3,4]pyrrolo[2,3-*b*]indol-4(5*H*)-one (**6f**)

Orange solid, Yield 40%, Melting point: 105–107 °C.

^{1}H NMR (300 MHz, $CDCl_3$) δ 7.12 (t, *J* = 7.43 Hz, 1H), 6.89(d, *J* = 7.29 Hz, 1H), 6.77 (t, *J* = 7.51 Hz, 1H), 6.66 (d, *J* = 7.85 Hz, 1H), 5.39–5.34 (m, 2H), 5.16 (s, 1H), 5.02 (s, 1H), 4.86 (d, *J* = 16.23 Hz, 1H), 3.96–3.91 (m, 2H), 2.70 (bs, 1H), 1.83–1.68 (m, 4H), 1.40–1.28 (m, 15H).

^{13}C NMR (75 MHz, $CDCl_3$) δ 181.6, 170.4, 154.6, 149.4, 130.4, 129.2, 123.8, 120.4, 111.3, 110.0, 81.9, 69.1, 52.3, 51.8, 39.4, 32.1, 30.4, 28.5, 25.9, 25.7, 25.4(2).

HRMS calculated for $C_{24}H_{31}N_3O_2$ 393.2416, found 393.2417.

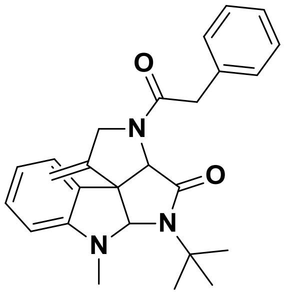

5-(*tert*-butyl)-6-methyl-1-methylene-3-(2-phenylacetyl)-1,2,3,3a,5a,6-hexahydropyrrolo[3',2':3,4]pyrrolo[2,3-*b*]indol-4(5*H*)-one (**6g**)

White solid, Yield 71%, Melting point: 181–183 °C.

^{1}H NMR (300 MHz, CDCl$_3$) δ 7.20–7.14 (m, 6H), 6.74–6.73 (m, 2H), 6.55 (d, *J* = 7.95 Hz, 1H), 5.37 (s, 1H), 5.07–5.04 (m, 2H), 4.77 (d, *J* = 16.85 Hz, 1H), 4.50 (s, 1H), 4.08–3.95 (m, 3H), 2.97 (s, 3H), 1.52 (s, 9H).

^{13}C NMR (75 MHz, CDCl$_3$) δ 172.4, 171.0, 151.2, 147.4, 135.0, 130.3, 129.2, 129.1, 128.4, 126.5, 123.0, 120.0, 111.6, 108.9, 90.3, 69.6, 60.8, 55.5, 51.0, 40.7, 36.3, 28.4.

HRMS calculated for $C_{26}H_{29}N_3O_2$ 415.2260, found 415.2228.

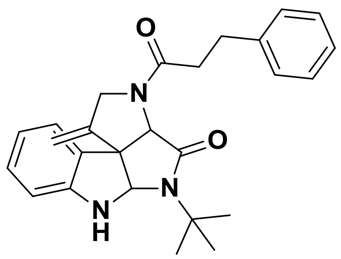

5-(*tert*-butyl)-1-methylene-3-(3-phenylpropanoyl)-1,2,3,3a,5a,6-hexahydropyrrolo[3',2':3,4]pyrrolo[2,3-*b*]indol-4(5*H*)-one (**6h**)

White solid, Yield 83%, Melting point: 179–181 °C.

^{1}H NMR (300 MHz, CDCl$_3$) δ 7.20–7.10 (m, 6H), 6.83–6.75 (m, 2H), 6.65 (d, *J* = 7.81 Hz, 1H), 5.48 (d, *J* = 4.28 Hz, 1H), 5.34 (s, 1H), 4.98 (s, 1H), 4.78 (d, *J* = 16.71 Hz, 1H), 4.44 (s, 1H), 4.38 (d, *J* = 3.86 Hz, 1H), 4.03 (d, *J* = 16.71 Hz, 1H), 3.13–2.97 (m, 4H), 1.47 (s, 9H).

^{13}C NMR (75 MHz, CDCl$_3$) δ 172.1, 171.0, 149.2, 149.0, 141.3, 130.6, 129.3, 128.4, 128.3, 125.9, 123.8, 120.6, 111.6, 110.1, 83.5, 69.6, 60.2, 55.2, 50.5, 35.6, 31.1, 28.0.

HRMS calculated for $C_{26}H_{29}N_3O_2$ 415.2260, found 415.2246.

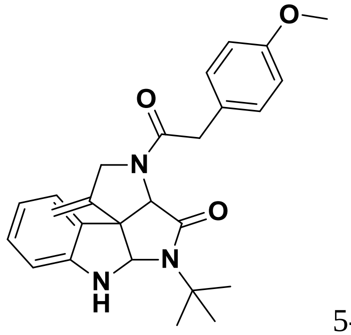

5-(*tert*-butyl)-3-(2-(4-methoxyphenyl)acetyl)-1-methylene-1,2,3,3a,5a,6-hexahydropyrrolo[3',2':3,4]pyrrolo[2,3-*b*]indol-4(5*H*)-one (**6i**)

Offwhite solid, Yield 69%, Melting point: 168–170 °C.

^{1}H NMR (300 MHz, CDCl$_3$) δ 7.17–7.08 (m, 3H), 6.79–6.63 (m, 5H), 5.48 (d, *J* = 4.08 Hz, 1H), 5.32 (s, 1H), 4.94 (s, 1H), 4.79 (d, *J* = 16.61 Hz, 1H), 4.56 (s, 1H), 4.40 (d, *J* = 4.14 Hz, 1H), 4.01 (d, *J* = 16.61 Hz, 1H), 1.49 (s, 9H).

^{13}C NMR (75 MHz, CDCl$_3$) δ 171.1(2), 158.3, 149.3, 148.9, 130.4, 130.1, 129.2, 127.0, 123.8, 120.6, 113.9, 111.6, 110.1, 83.6, 69.5, 60.1, 55.3, 55.2, 50.5, 40.0, 28.0.

HRMS calculated for $C_{26}H_{29}N_3O_3$ 431.2209, found 431.2211.

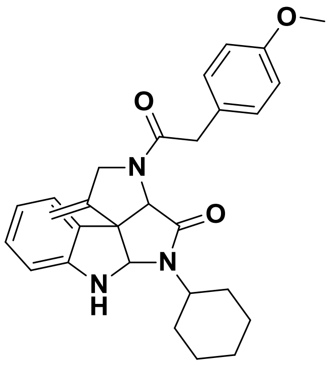

5-cyclohexyl-3-(2-(4-methoxyphenyl)acetyl)-1-methylene-1,2,3,3a,5a,6-hexahydropyrrolo[3',2':3,4]pyrrolo[2,3-*b*]indol-4(5*H*)-one (**6j**)

White solid, Yield 75%, Melting point: 153–155 °C.

^{1}H NMR (300 MHz, CDCl$_3$) δ 7.20–7.09 (m, 3H), 6.81–6.63 (m, 5H), 5.36 (d, *J* = 3.95 Hz, 1H), 5.28 (s, 1H), 4.86–4.76 (m, 2H), 4.62 (s, 1H), 4.42 (d, *J* = 3.59 Hz, 1H), 4.03–3.97 (m, 3H), 3.92–3.85 (m, 1H), 3.76 (m, 3H), 2.03–1.99 (m, 1H), 1.86–1.69 (m, 4H), 1.52–1.25 (m, 5H).

^{13}C NMR (75 MHz, CDCl$_3$) δ 170.1, 170.1, 158.4, 148.9, 148.8, 130.1, 130.0, 129.3, 127.0, 123.9, 120.6, 114.0, 111.4, 110.3, 81.8, 68.7, 61.3, 55.2, 52.8, 50.7, 39.9, 32.0, 30.3, 25.7, 25.5, 25.4.

HRMS calculated for $C_{28}H_{31}N_3O_3$ 457.2365, found 457.2359.

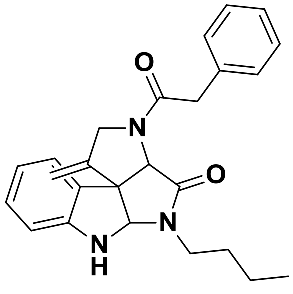

5-butyl-1-methylene-3-(2-phenylacetyl)-1,2,3,3a,5a,6-hexahydropyrrolo[3',2':3,4]pyrrolo[2,3-*b*]indol-4(5*H*)-one (**6k**)

Yellow solid, Yield 80%, Melting point: 42–44 °C.

^{1}H NMR (300 MHz, CDCl$_3$) δ 7.29–7.28 (m, 5H), 7.13 (t, *J* = 7.59 Hz, 1H), 6.79–6.65 (m, 3H), 5.24–5.20 (m, 2H), 4.83 (d, *J* = 16.34 Hz, 1H), 4.72 (s, 1H), 4.58–4.47 (m, 2H), 4.21 (d, *J* = 15.52 Hz, 1H), 4.08–3.99 (m, 2H), 3.61–3.53 (m, 1H), 3.23–3.14 (m, 1H), 1.58–1.56 (m, 2H), 1.37–1.30 (m, 2H), 0.95 (t, *J* = 7.44 Hz, 3H).

^{13}C NMR (75 MHz, CDCl$_3$) δ 170.9, 169.9, 148.9, 148.2, 135.0, 129.7, 129.5, 129.1, 128.5, 126.7, 124.4, 121.1, 111.2, 111.0, 82.3, 68.1, 61.6, 50.7, 41.0, 40.9, 29.1, 20.2, 13.7.

HRMS calculated for $C_{25}H_{27}N_3O_2$ 401.2103, found 401.2101.

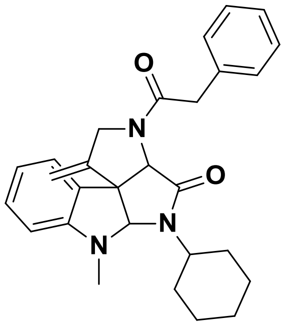

5-cyclohexyl-6-methyl-1-methylene-3-(2-phenylacetyl)-1,2,3,3a,5a,6-hexahydropyrrolo[3',2':3,4]pyrrolo[2,3-*b*]indol-4(5*H*)-one (**6l**)

White solid, Yield 74%, Melting point: 70–72 °C.

^{1}H NMR (300 MHz, CDCl$_3$) δ 7.25–7.18 (m, 6H), 6.72–6.55 (m, 3H), 5.28 (s, 1H), 4.81–4.76 (m, 3H), 4.54 (s, 1H), 4.06–4.03 (m, 3H), 3.57 (m, 1H), 3.03 (s, 3H), 2.00–1.71 (m, 6H), 1.25–1.18 (m, 4H).

^{13}C NMR (75 MHz, CDCl$_3$) δ 170.9, 170.7, 151.6, 148.6, 135.0, 130.1, 129.5, 129.1, 128.5, 126.6, 123.5, 120.1, 111.5, 109.2, 91.1, 68.8, 60.8, 55.6, 50.8, 40.8, 37.8, 30.3, 29.5, 26.0, 25.9, 25.3.

HRMS calculated for $C_{28}H_{31}N_3O_2$ 441.2416, found 441.2400.

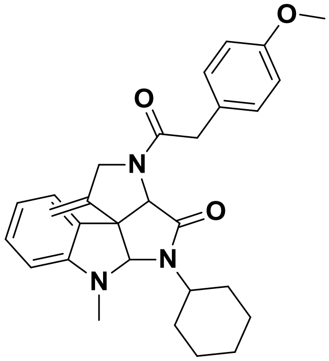

5-cyclohexyl-3-(2-(4-methoxyphenyl)acetyl)-6-methyl-1-methylene-1,2,3,3a,5a,6-hexahydropyrrolo[3',2':3,4]pyrrolo[2,3-*b*]indol-4(5*H*)-one (**6m**)

White solid, Yield 68%, Melting point: 146–148 °C.

^{1}H NMR (300 MHz, CDCl$_3$) δ 7.20–7.16 (m, 3H), 6.80 (d, *J* = 8.61 Hz, 2H), 6.75–6.65 (m, 2H), 6.56 (d, *J* = 8.07 Hz, 1H), 5.28 (s, 1H), 4.83–4.75 (m, 3H), 4.55 (s, 1H), 4.05–3.96 (m, 3H), 3.76 (s, 3H), 3.61–3.53 (m, 1H), 3.03 (s, 3H), 1.89–1.81 (m, 5H), 1.31–1.22 (m, 5H).

^{13}C NMR (75 MHz, CDCl$_3$) δ 171.2, 170.7, 158.4, 151.6, 148.7, 130.1, 129.4, 127.0, 123.5, 120.0, 113.9, 111.5, 109.2, 91.1, 68.8, 60.8, 55.6, 55.2, 50.8, 39.9, 37.8, 30.3, 29.5, 26.0, 25.9, 25.3.

HRMS calculated for $C_{29}H_{33}N_3O_3$ 471.2522, found 471.2541.

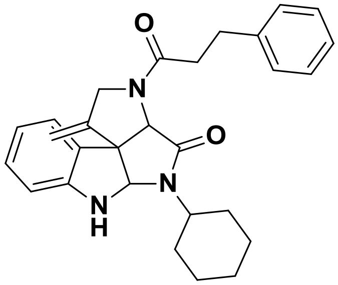

5-cyclohexyl-1-methylene-3-(3-phenylpropanoyl)-1,2,3,3a,5a,6-hexahydropyrrolo[3',2':3,4]pyrrolo[2,3-*b*]indol-4(5*H*)-one (**6n**)

White solid, Yield 72%, Melting point: 73–75 °C.

^{1}H NMR (300 MHz, CDCl$_3$) δ 7.22–7.14 (m, 6H), 6.83–6.65 (m, 3H), 5.37–5.30 (m, 2H), 4.90 (s, 1H), 4.77 (d, *J* = 16.88 Hz, 1H), 4.50 (s, 1H), 4.40 (s, 1H), 4.02 (d, *J* = 16.94 Hz, 1H), 3.86 (m, 1H), 3.18 (m, 1H), 3.03 (m, 2H), 2.73 (m,1H), 1.83–1.73 (m, 4H), 1.57–1.16 (m, 6H).

^{13}C NMR (75 MHz, CDCl$_3$) δ 172.2, 170.1, 149.0, 148.7, 141.3, 130.1, 129.4, 128.4, 128.3, 125.9, 124.0, 120.6, 111.4, 110.3, 81.7, 68.9, 61.4, 52.7, 50.7, 35.6, 32.0, 31.2, 30.3, 25.7, 25.5, 25.4.

HRMS calculated for $C_{28}H_{31}N_3O_2$ 441.2416, found 441.2414.

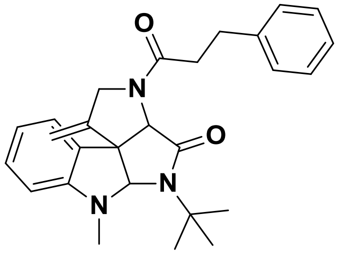

5-(*tert*-butyl)-6-methyl-1-methylene-3-(3-phenylpropanoyl)-1,2,3,3a,5a,6-hexahydropyrrolo[3',2':3,4]pyrrolo[2,3-*b*]indol-4(5*H*)-one (**6o**)

White solid, Yield 60%, Melting point: 54–56 °C.

^{1}H NMR (300 MHz, CDCl$_3$) δ 7.25–7.19 (m, 6H), 6.82–6.78 (m, 2H), 6.55 (bs, 1H), 5.39 (s, 1H), 5.07 (s, 2H), 4.75 (d, *J* = 16.63 Hz, 1H), 4.38 (s, 1H), 4.04 (d, *J* = 16.69 Hz, 1H), 2.95(m, 6H), 2.65 (m, 1H), 1.49 (s, 9H).

^{13}C NMR (75 MHz, CDCl$_3$) δ 172.5, 172.3, 151.1, 147.2, 141.3, 130.4, 128.4, 128.3, 125.9, 123.0, 119.9, 111.6, 108.6, 90.1, 69.7, 60.8, 55.4, 50.9, 35.8, 35.6, 31.1, 28.4.

HRMS calculated for $C_{27}H_{31}N_3O_2$ 429.2416, found 429.2395.

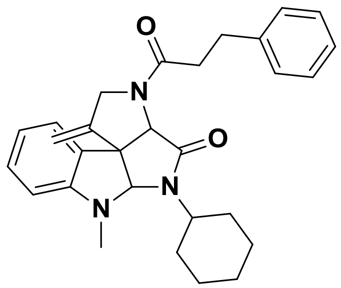

5-cyclohexyl-6-methyl-1-methylene-3-(3-phenylpropanoyl)-1,2,3,3a,5a,6-hexahydropyrrolo[3',2':3,4]pyrrolo[2,3-*b*]indol-4(5*H*)-one (**6p**)

White solid, Yield 84%, Melting point: 125–127 °C.

^{1}H NMR (300 MHz, CDCl$_3$) δ 7.22–7.17 (m, 6H), 6.81–6.75 (m, 2H), 6.56 (d, *J* = 8.01 Hz, 1H), 5.30 (s, 1H), 4.86–4.73 (m, 3H), 4.43 (s, 1H), 4.04 (d, *J* = 16.67 Hz, 1H), 3.57–3.49 (m, 1H), 3.18–3.12 (m, 1H), 3.10–2.98 (m, 5H), 2.77–2.67 (m, 1H), 1.90–1.70 (m, 6H), 1.31–1.20 (m, 4H).

^{13}C NMR (75 MHz, CDCl$_3$) δ 172.2, 170.7, 151.7, 148.5, 141.3, 130.3, 129.5, 128.4, 128.3, 125.9, 123.5, 120.0, 111.5, 109.1, 91.1, 69.0, 60.9, 55.6, 50.7, 37.6, 35.6, 31.2, 30.3, 29.4, 26.0, 25.9, 25.2.

HRMS calculated for $C_{29}H_{33}N_3O_2$ 455.2573, found 455.2599.

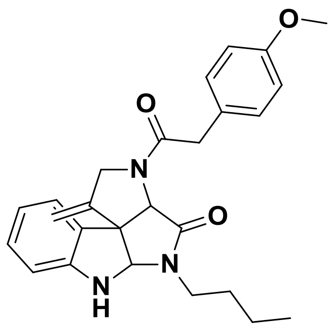

5-butyl-3-(2-(4-methoxyphenyl)acetyl)-1-methylene-1,2,3,3a,5a,6-hexahydropyrrolo[3',2':3,4]pyrrolo[2,3-*b*]indol-4(5*H*)-one (**6q**)

Offwhite solid, Yield 69%, Melting point: 49–51 °C.

^{1}H NMR (300 MHz, CDCl$_3$) δ 7.21 (d, *J* = 8.53 Hz, 2H), 7.21 (t, *J* = 7.52 Hz, 1H), 6.84–6.75 (m, 3H), 6.70–6.67 (m, 2H), 5.24–5.20 (m, 2H), 4.81 (d, *J* = 16.19 Hz, 1H), 4.72 (s, 1H), 4.59 (s, 1H), 4.50 (d, *J* = 4.40 Hz, 1H), 4.13–3.97 (3H), 3.78 (s, 3H), 3.63–3.53 (m, 1H), 3.23–3.14 (m, 1H), 1.37–1.25 (m, 4H), 0.95 (t, *J* = 7.35 Hz, 1H).

^{13}C NMR (75 MHz, CDCl$_3$) δ 171.2, 169.9, 158.4, 148.9, 148.2, 130.1, 129.8, 129.5, 127.0, 124.4, 121.0, 114.0, 111.2, 111.0, 82.3, 68.1, 61.6, 55.2, 50.7, 41.0, 40.0, 29.1, 20.1, 13.7.

HRMS calculated for $C_{26}H_{29}N_3O_3$ 431.2209, found 431.2203.

^{1}H and ^{13}C NMR spectra of compound **5a** (300 MHz, $CDCl_3$).

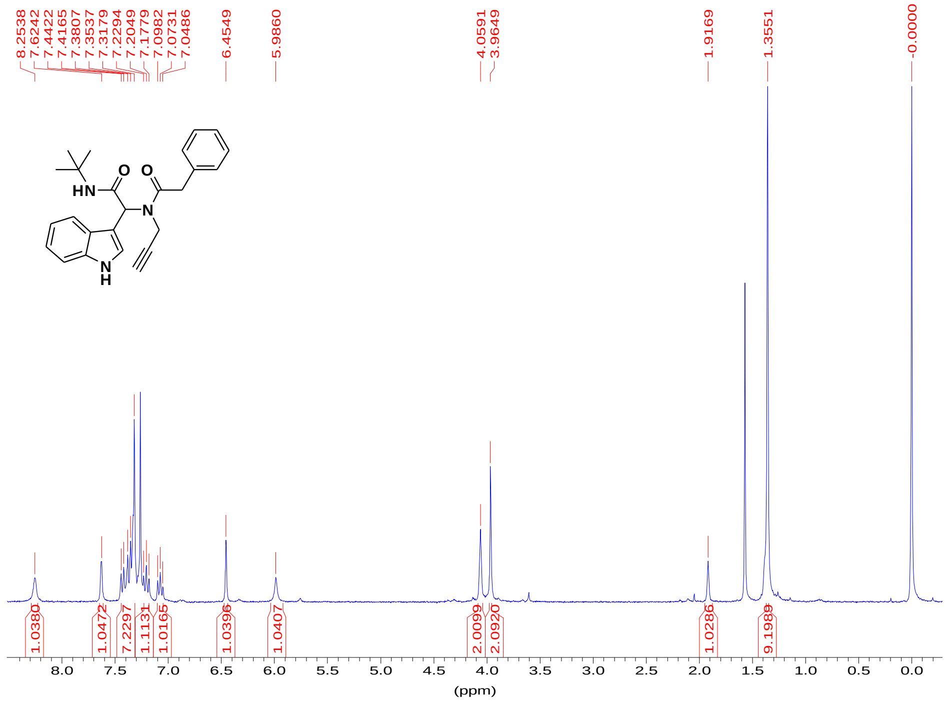

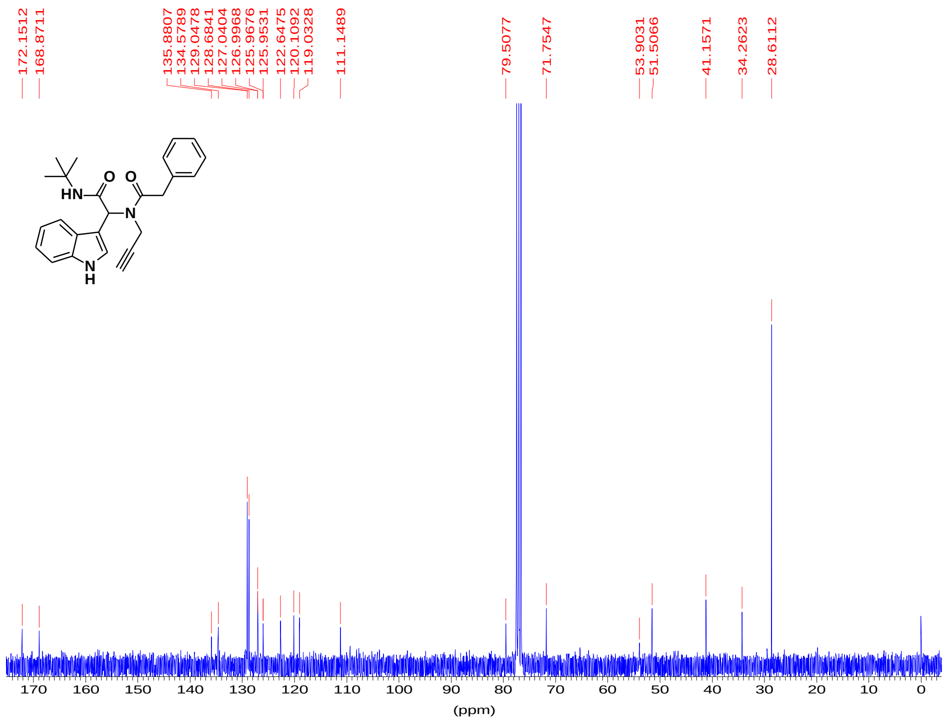

^{1}H and ^{13}C NMR spectra of compound **5b** (300 MHz, $CDCl_3$).

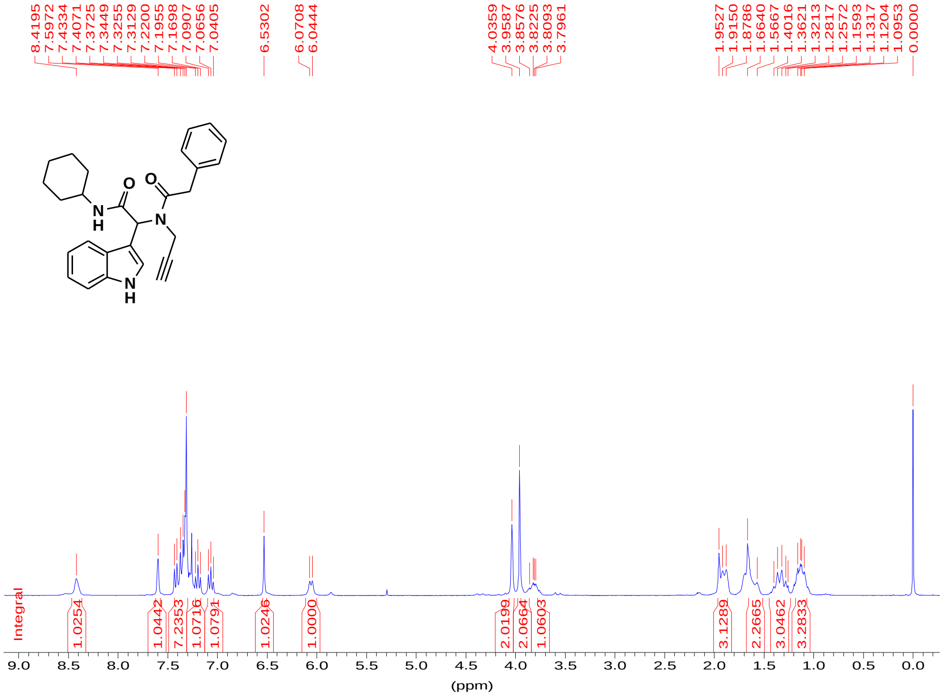

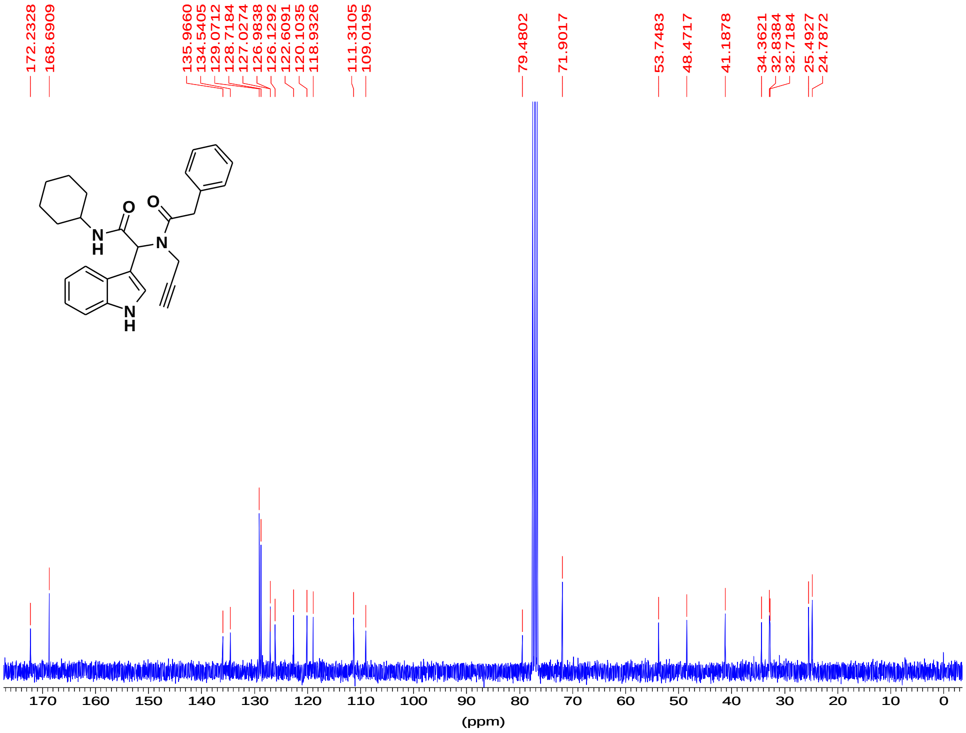

^{1}H and ^{13}C NMR spectra of compound **5c** (300 MHz, $CDCl_3$).

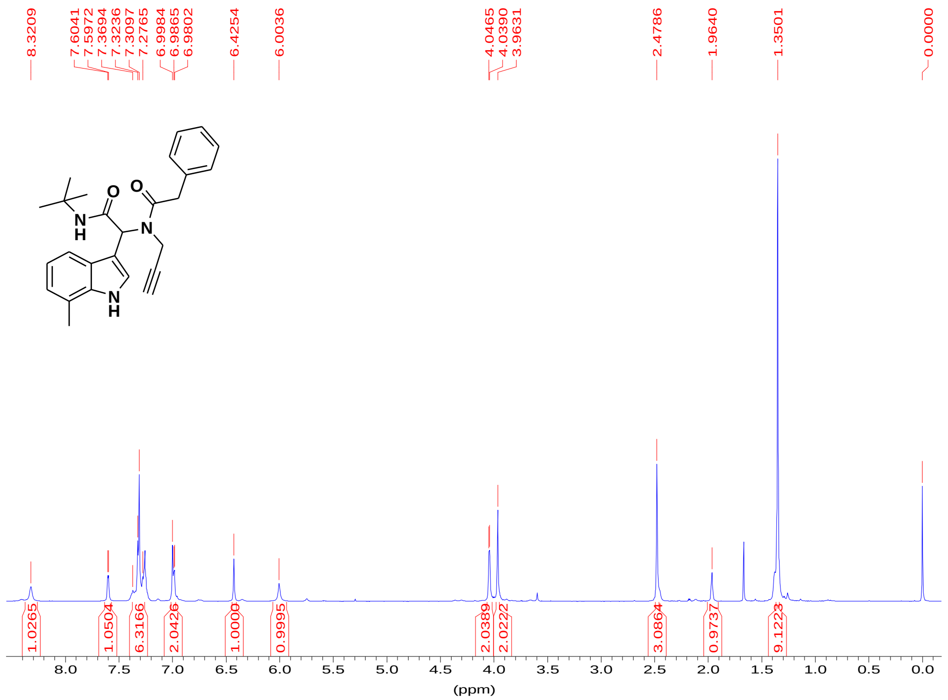

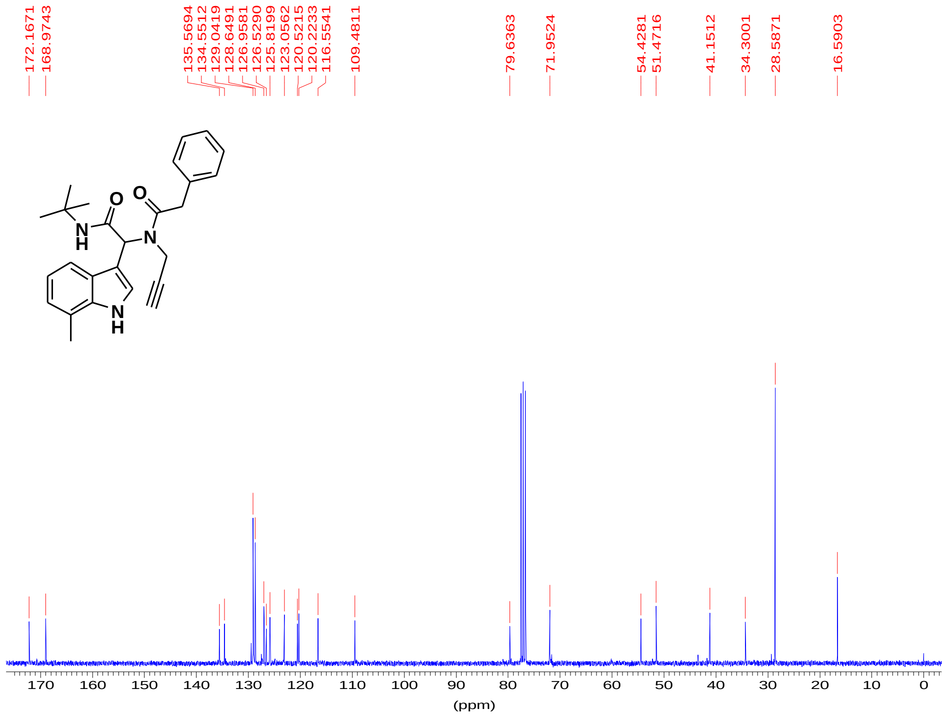

^{1}H and ^{13}C NMR spectra of compound **5d** (300 MHz, $CDCl_3$).

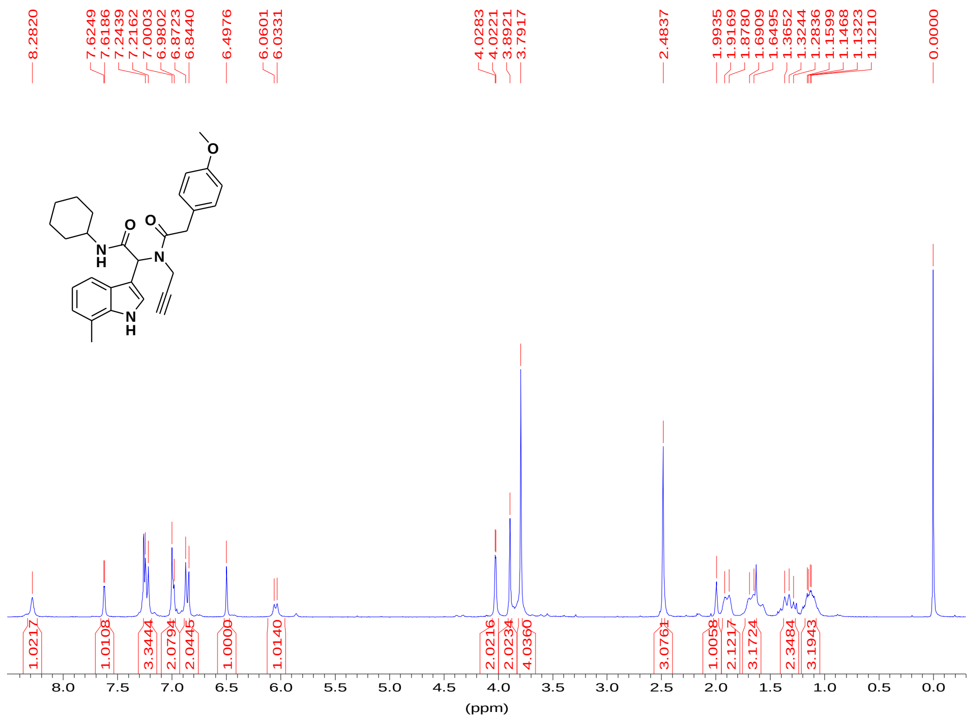

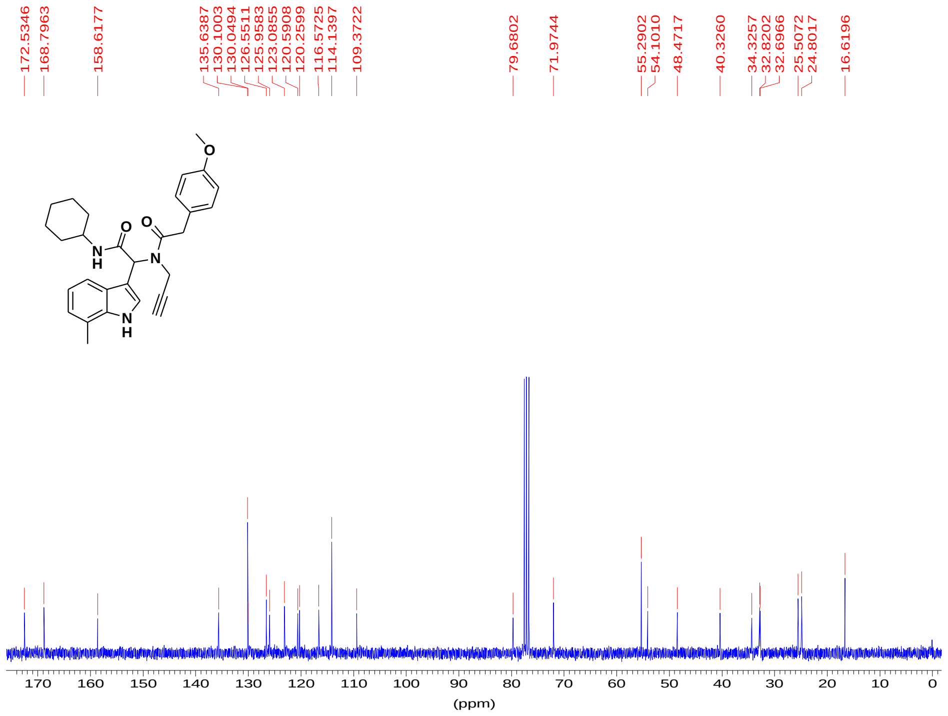

^{1}H and ^{13}C NMR spectra of compound **5e** (300 MHz, $CDCl_3$).

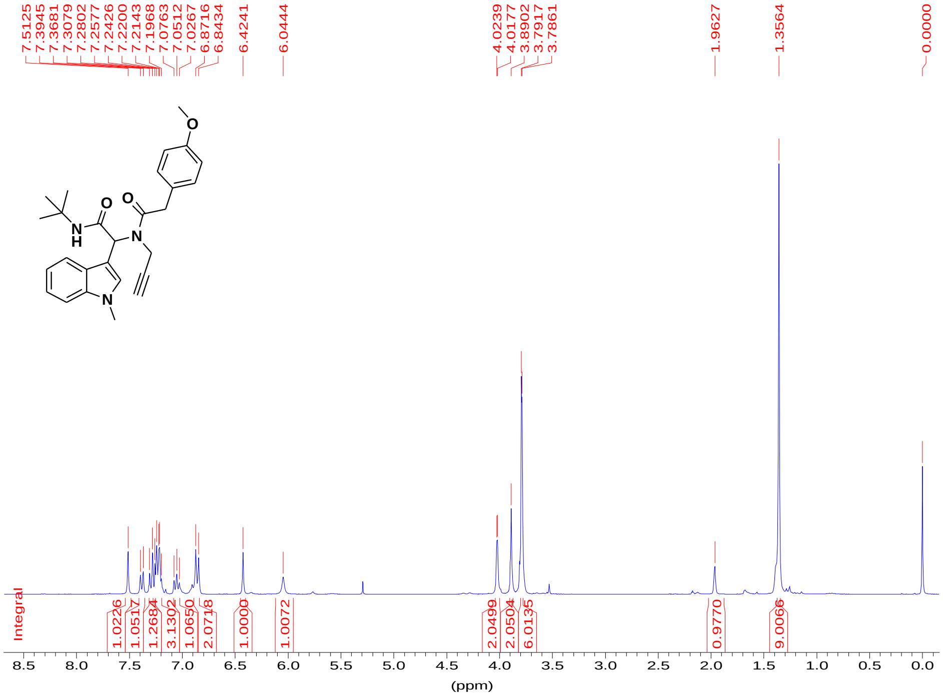

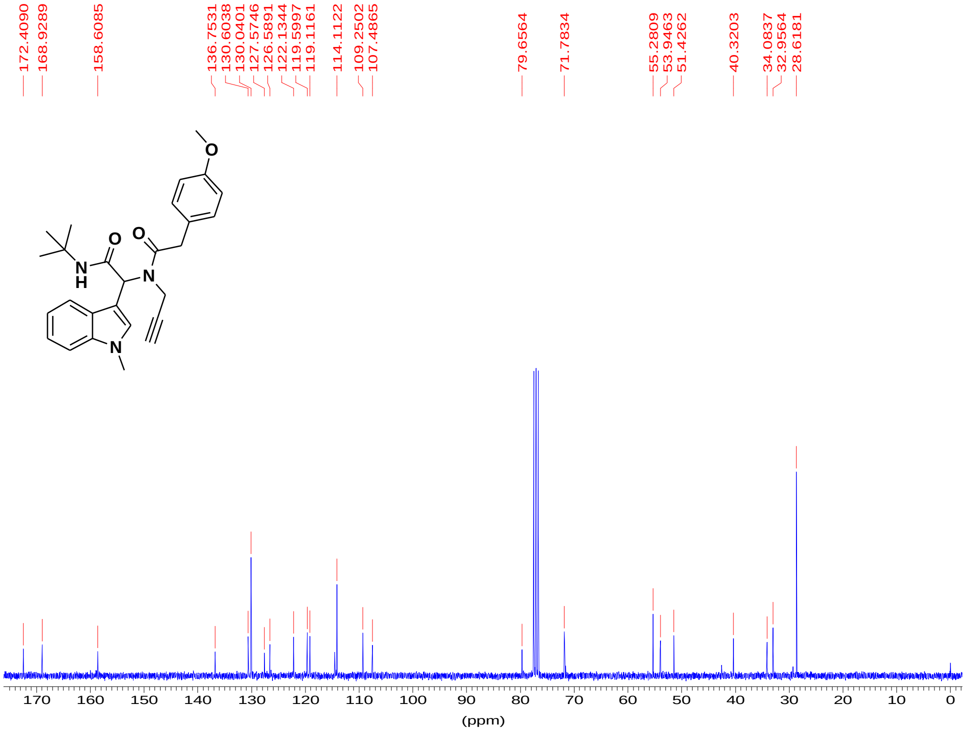

^{1}H and ^{13}C NMR spectra of compound **5f** (300 MHz, $CDCl_3$).

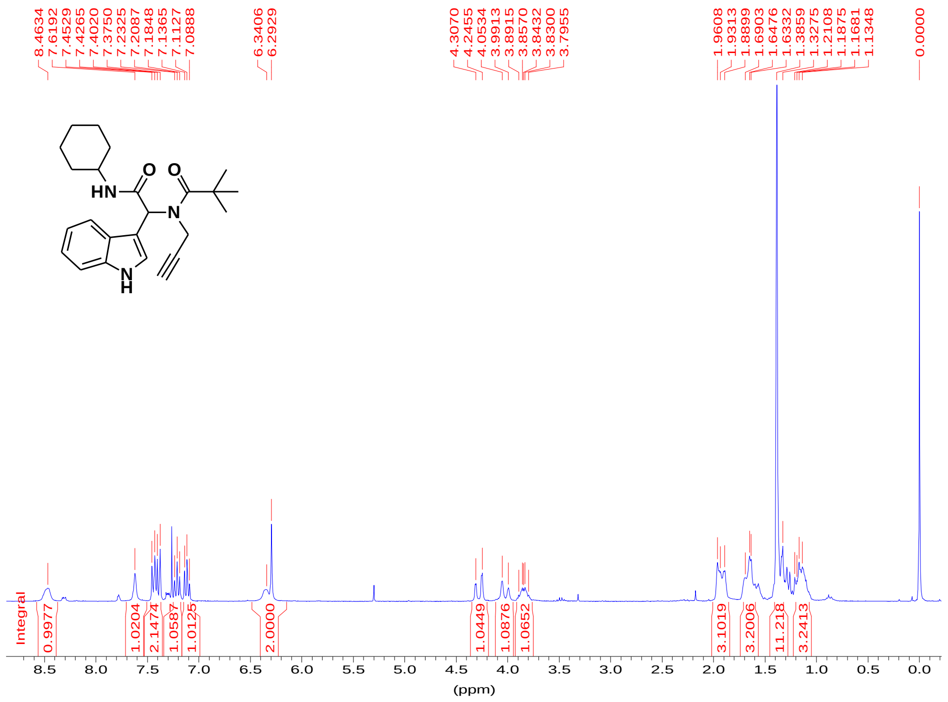

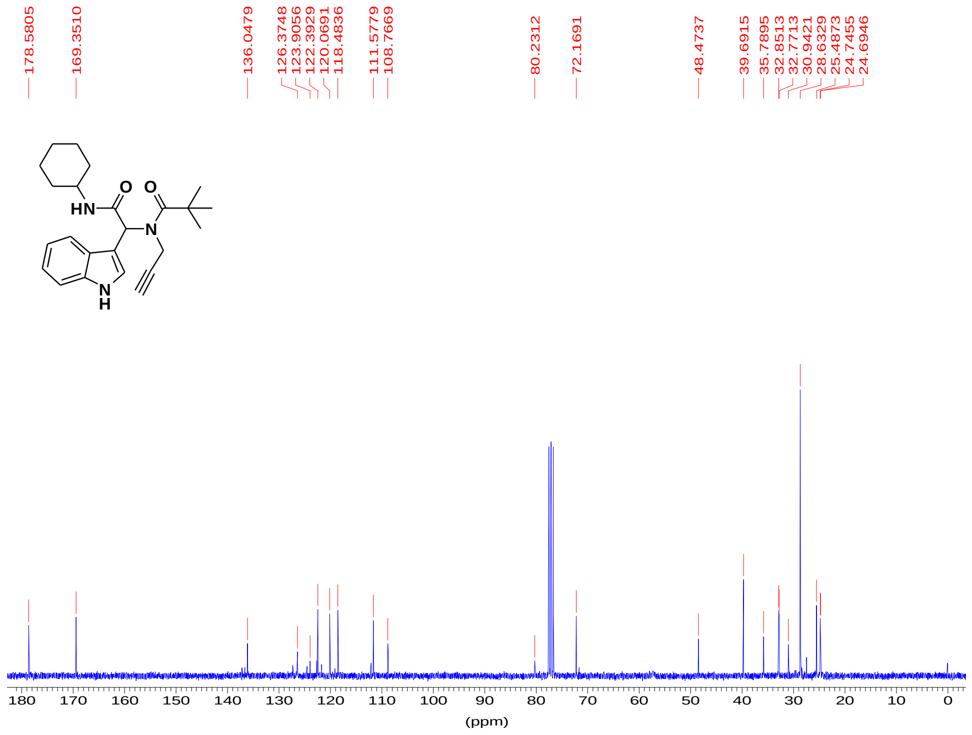

^{1}H and ^{13}C NMR spectra of compound **5g** (300 MHz, $CDCl_3$).

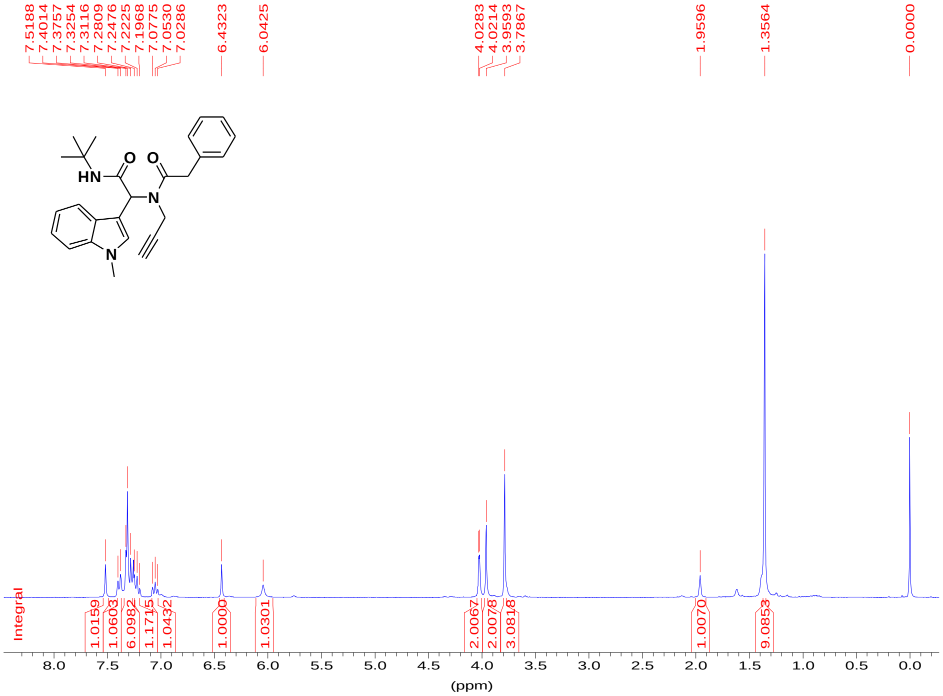

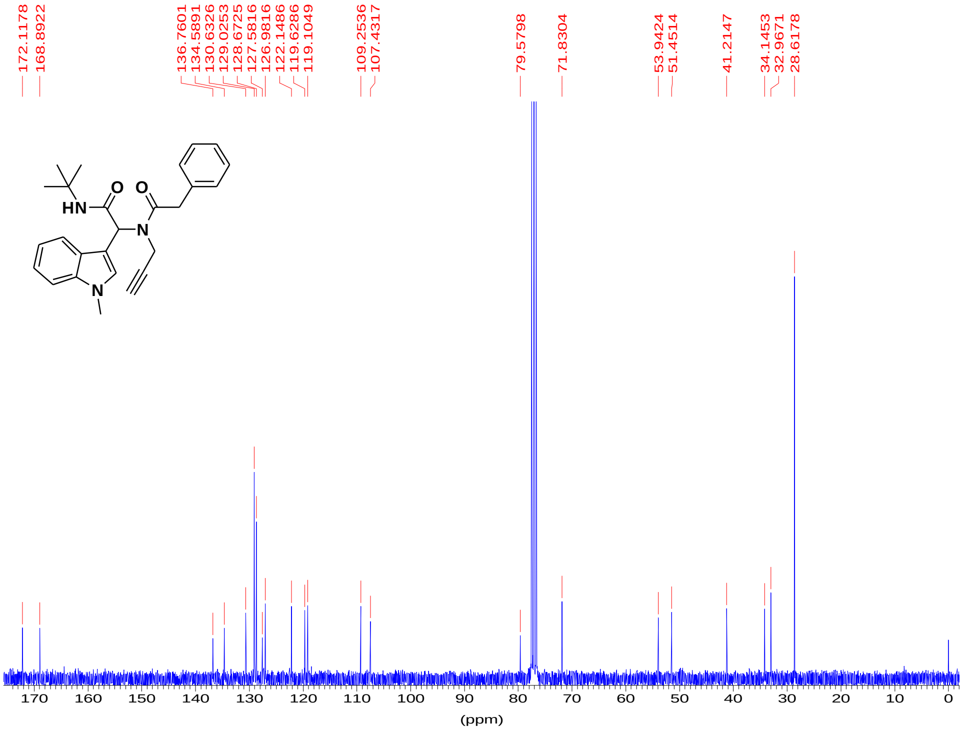

^{1}H and ^{13}C NMR spectra of compound **5h** (300 MHz, $CDCl_3$).

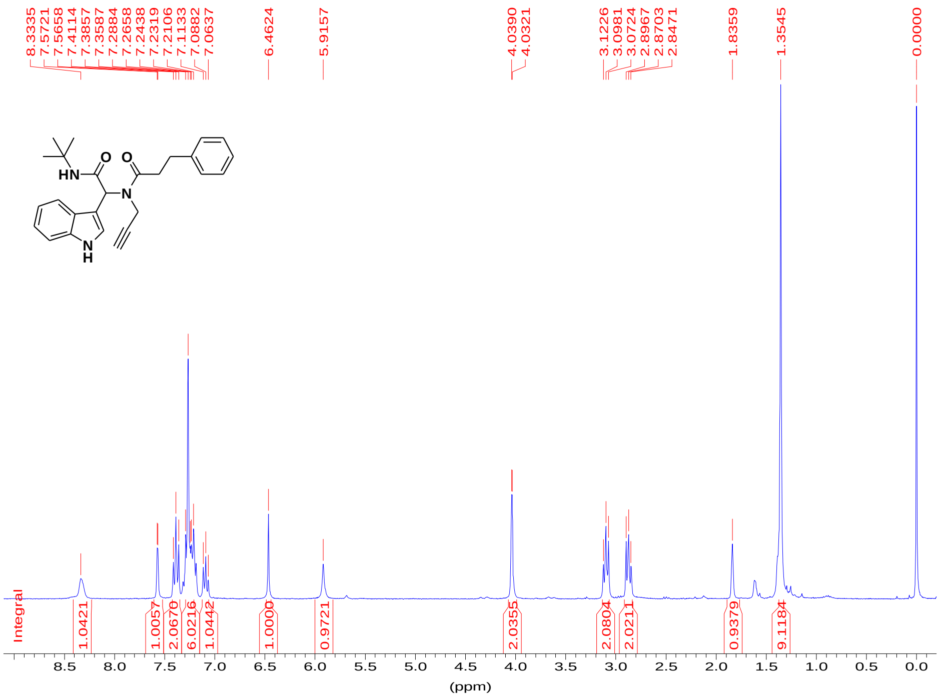

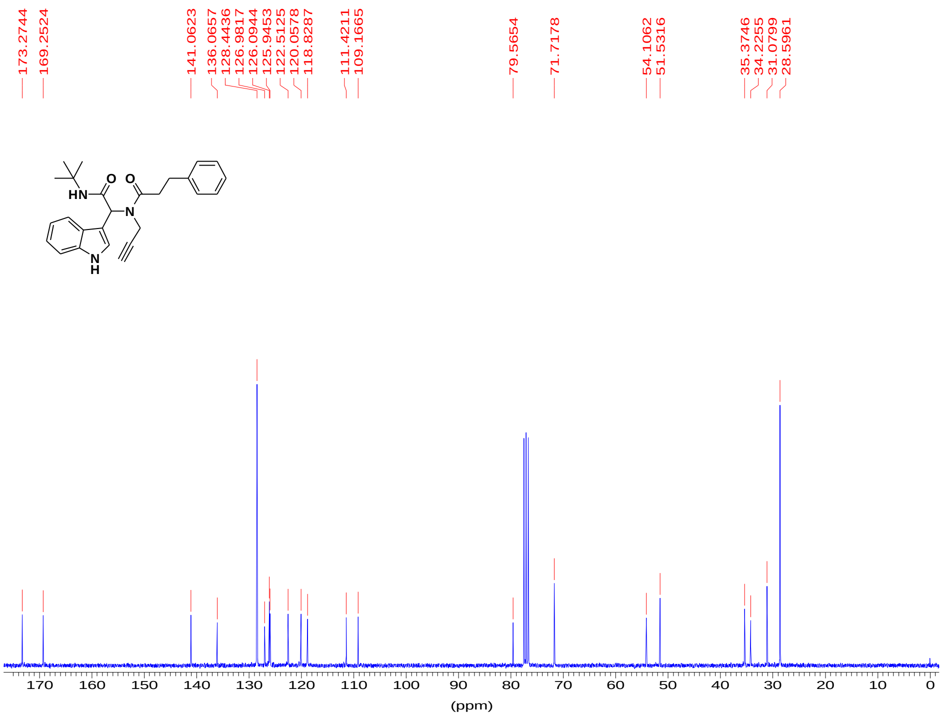

^{1}H and ^{13}C NMR spectra of compound **5i** (300 MHz, $CDCl_3$).

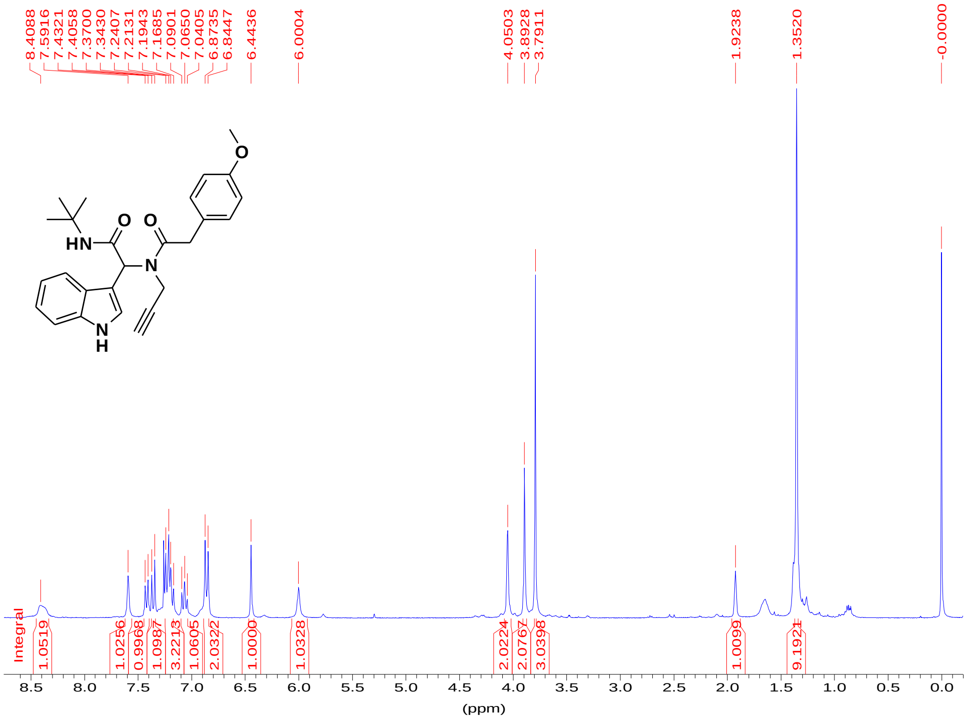

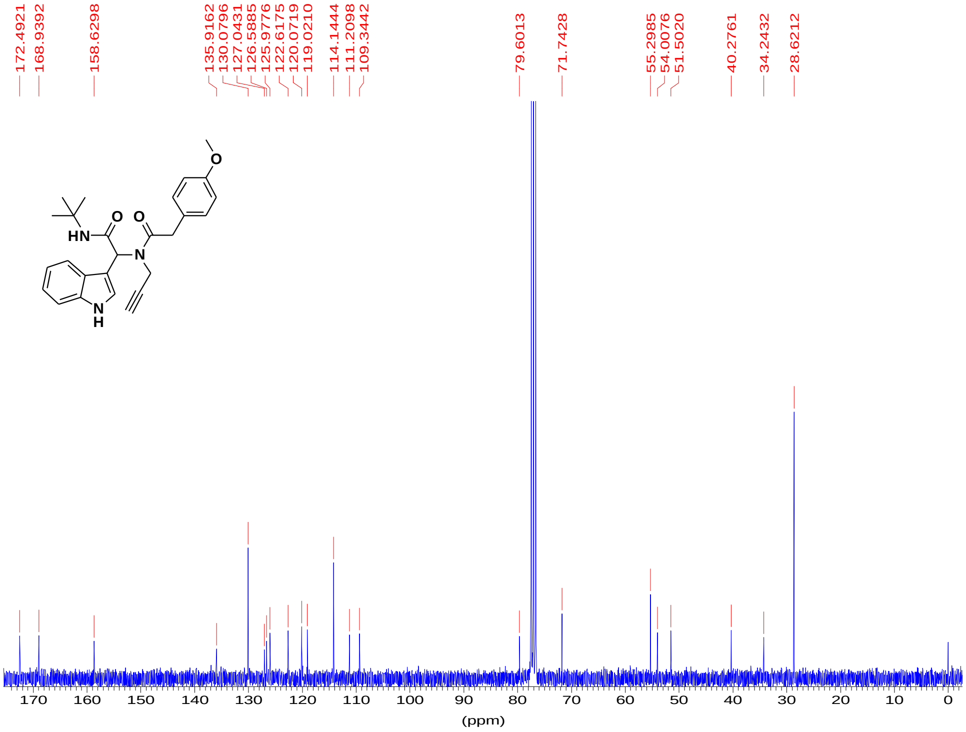

^{1}H and ^{13}C NMR spectra of compound **5j** (300 MHz, $CDCl_3$).

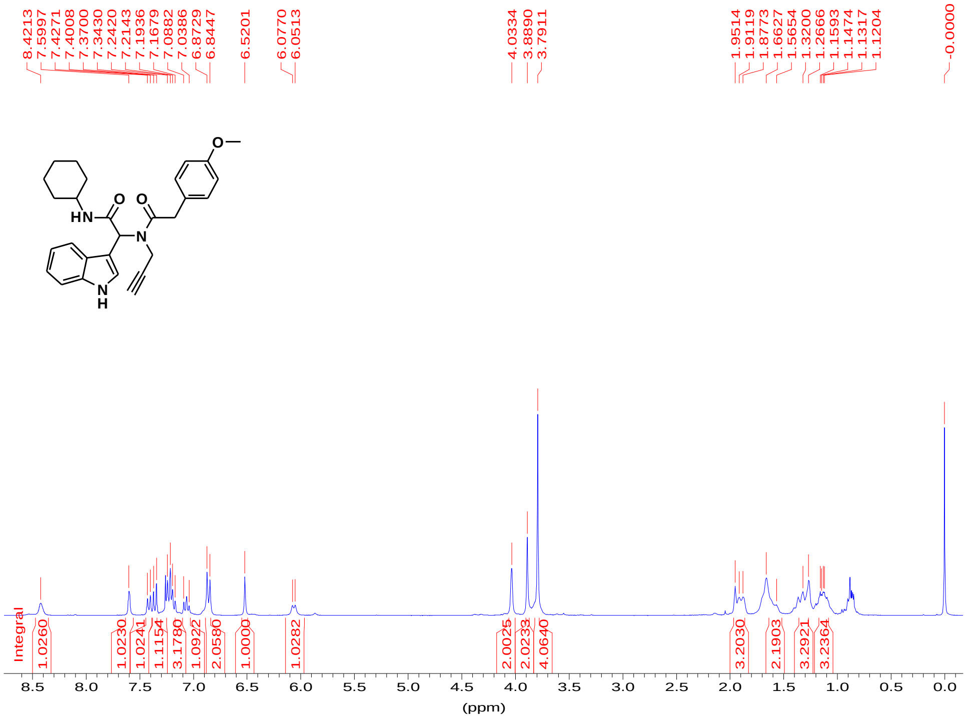

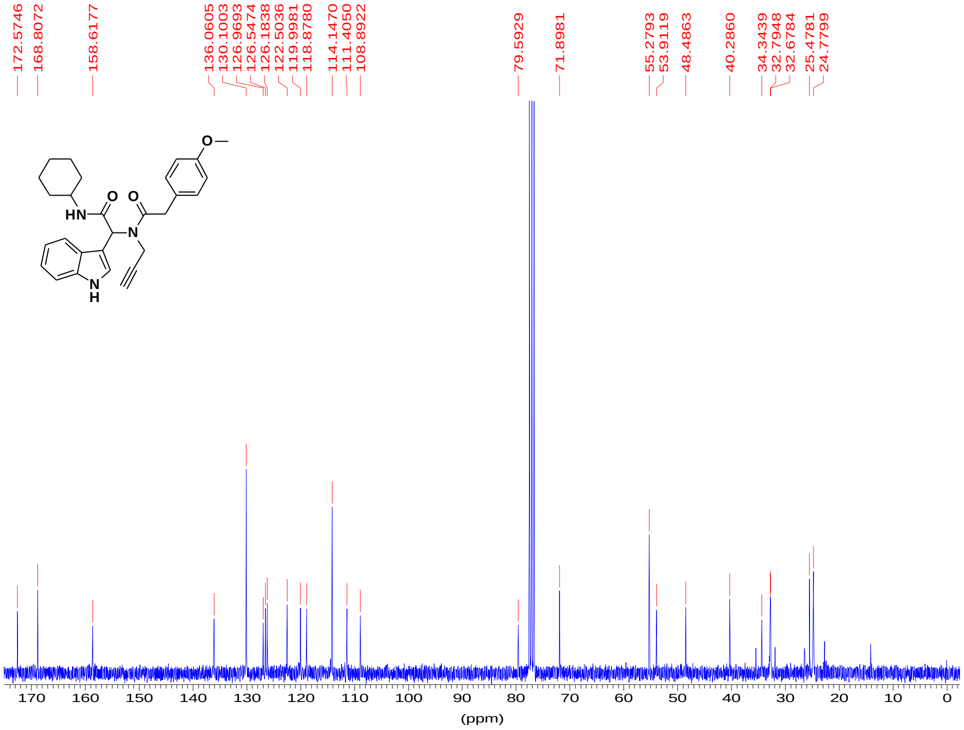

^{1}H and ^{13}C NMR spectra of compound **5k** (300 MHz, $CDCl_3$).

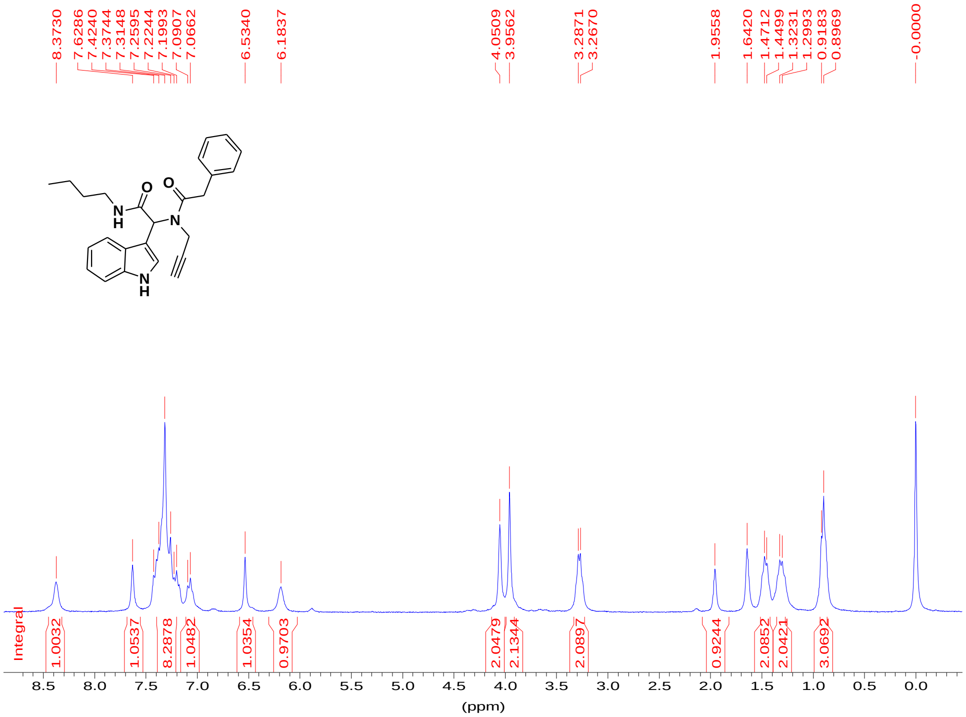

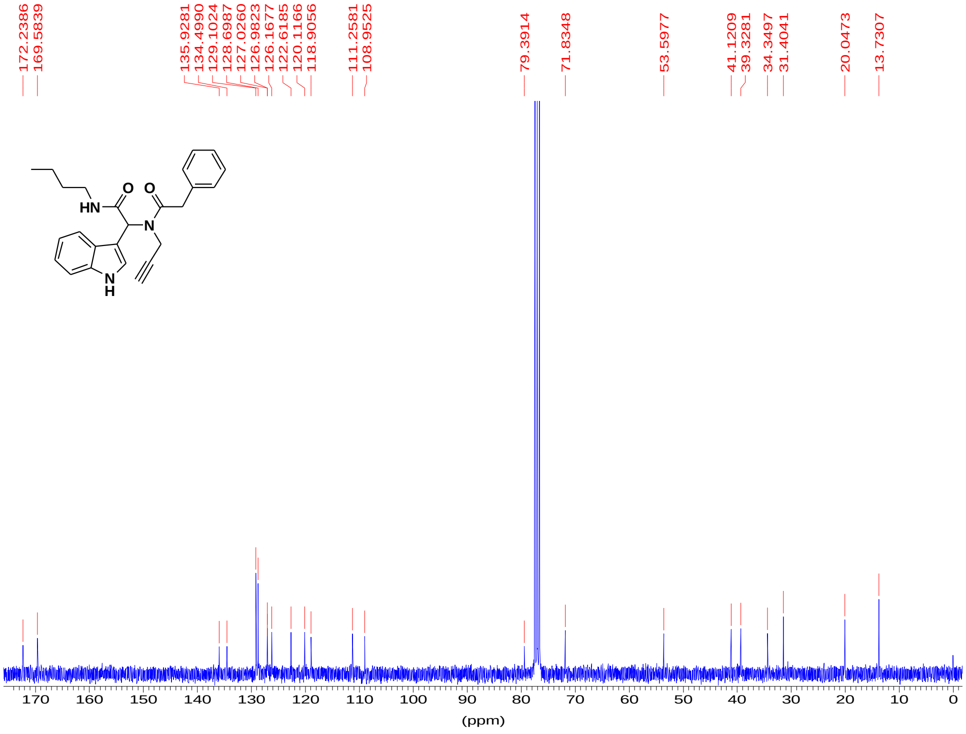

^{1}H and ^{13}C NMR spectra of compound **5l** (300 MHz, $CDCl_3$).

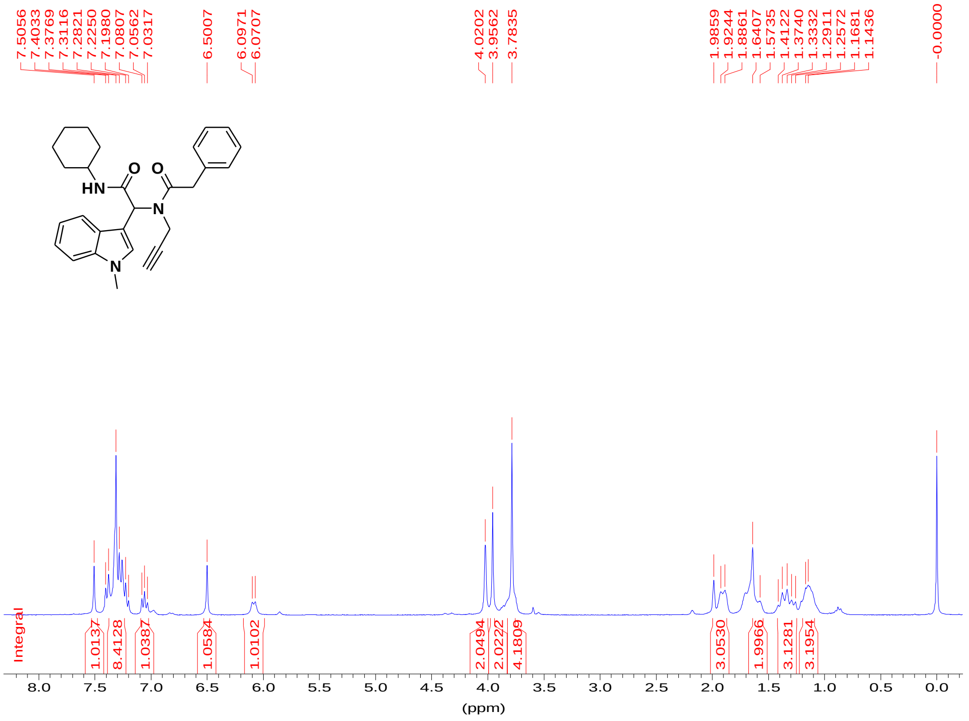

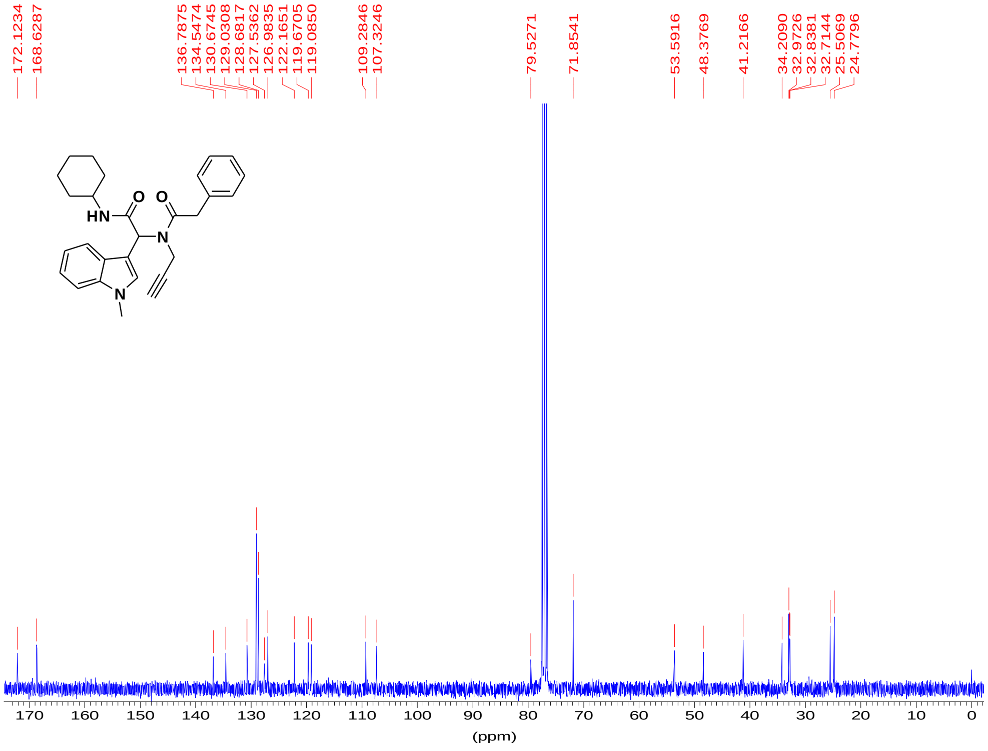

^{1}H and ^{13}C NMR spectra of compound **5m** (300 MHz, $CDCl_3$).

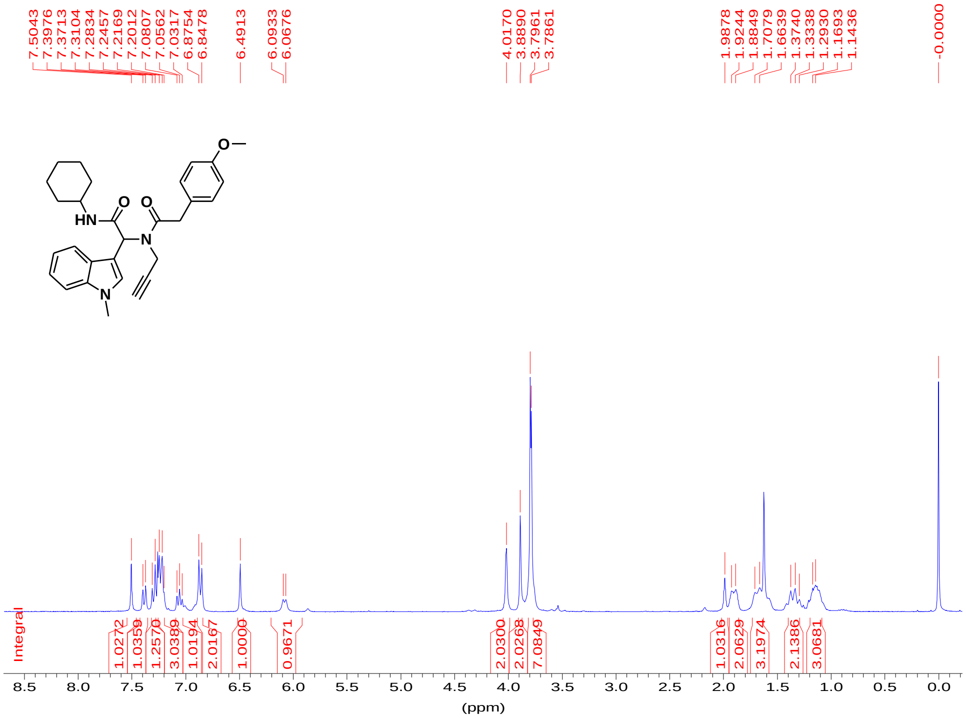

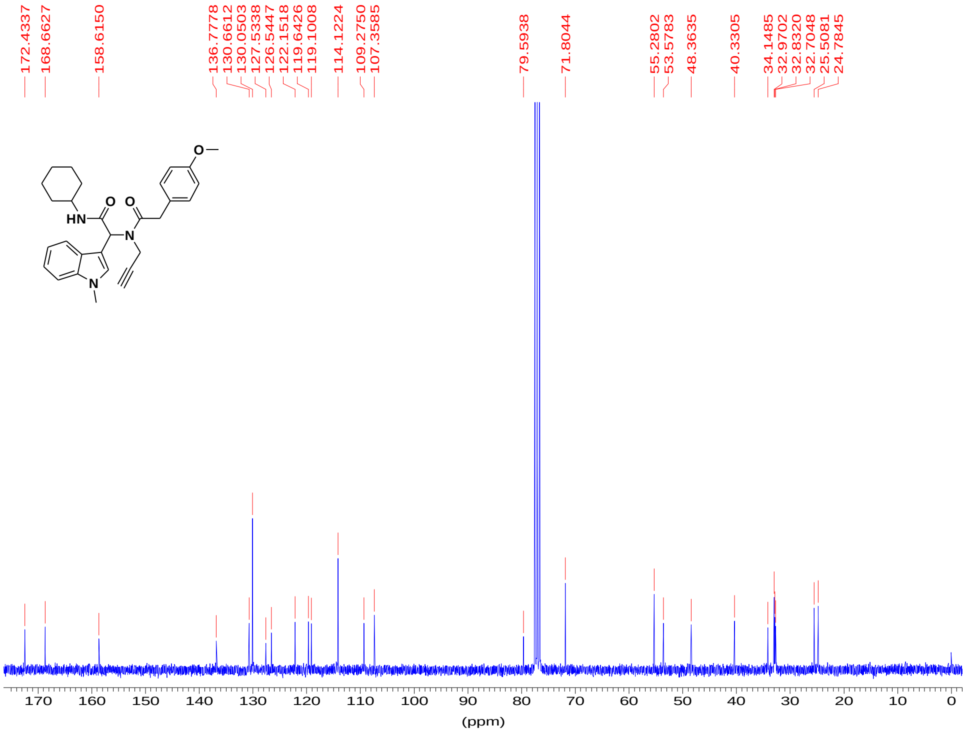

^{1}H and ^{13}C NMR spectra of compound **5n** (300 MHz, $CDCl_3$).

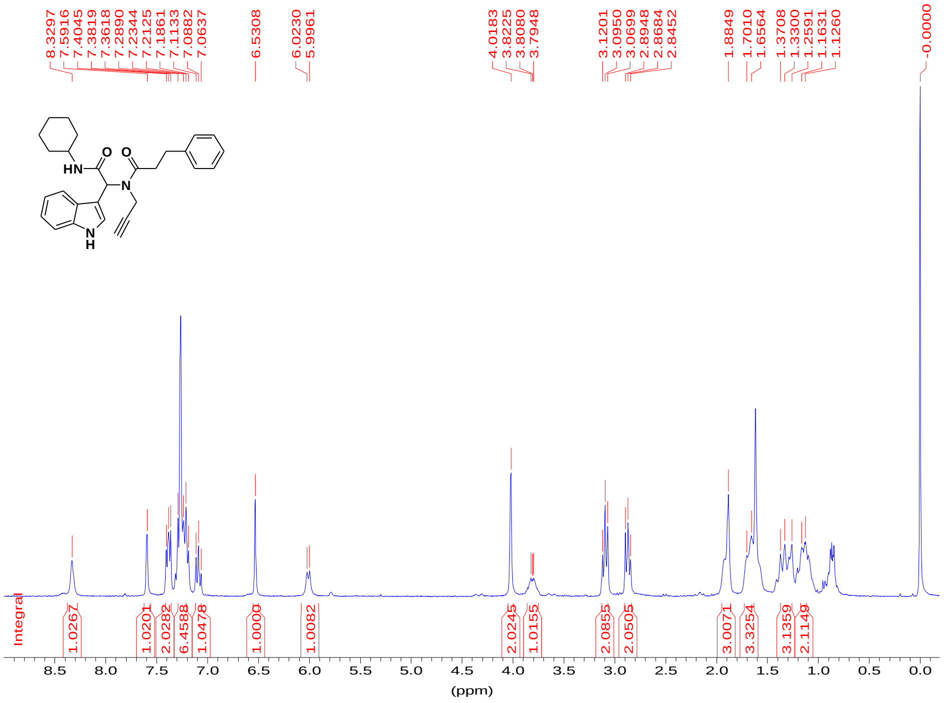

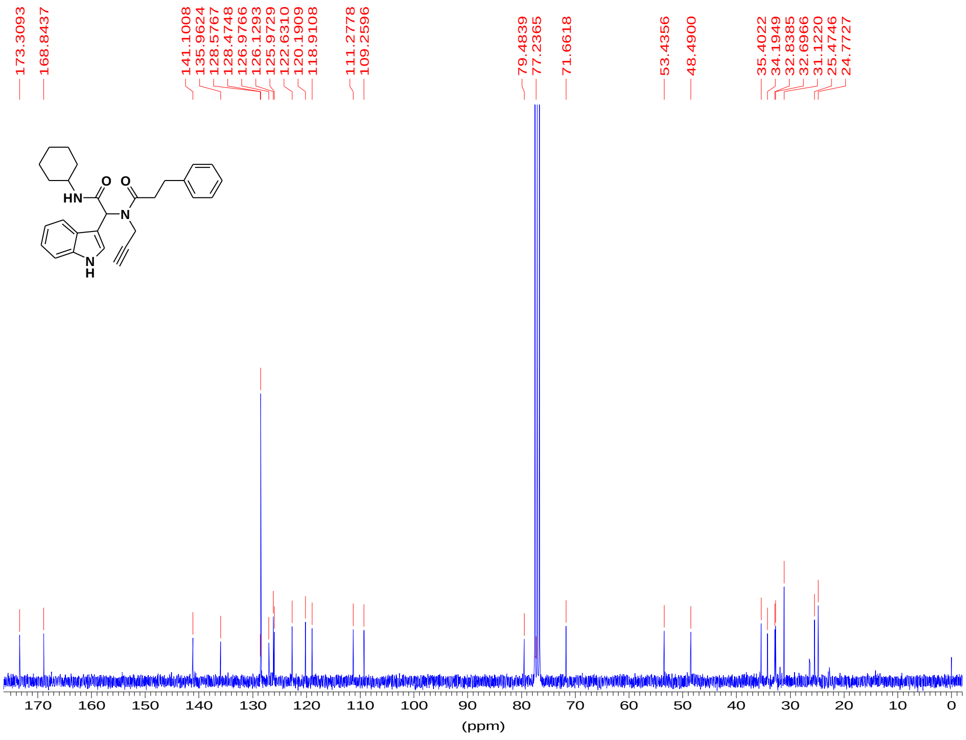

^{1}H and ^{13}C NMR spectra of compound **5o** (300 MHz, $CDCl_3$).

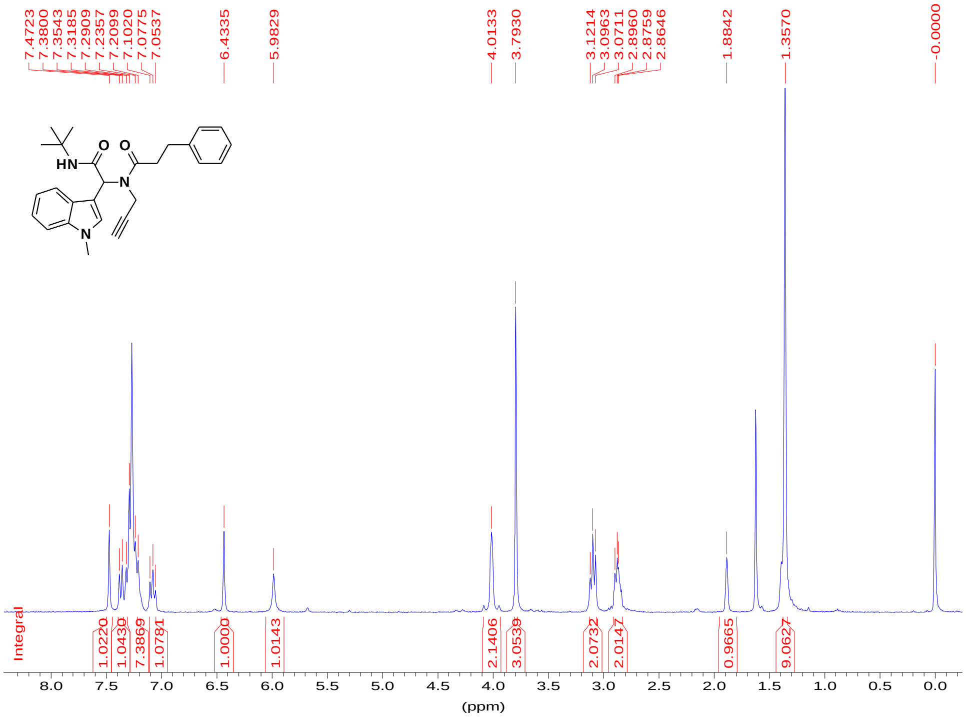

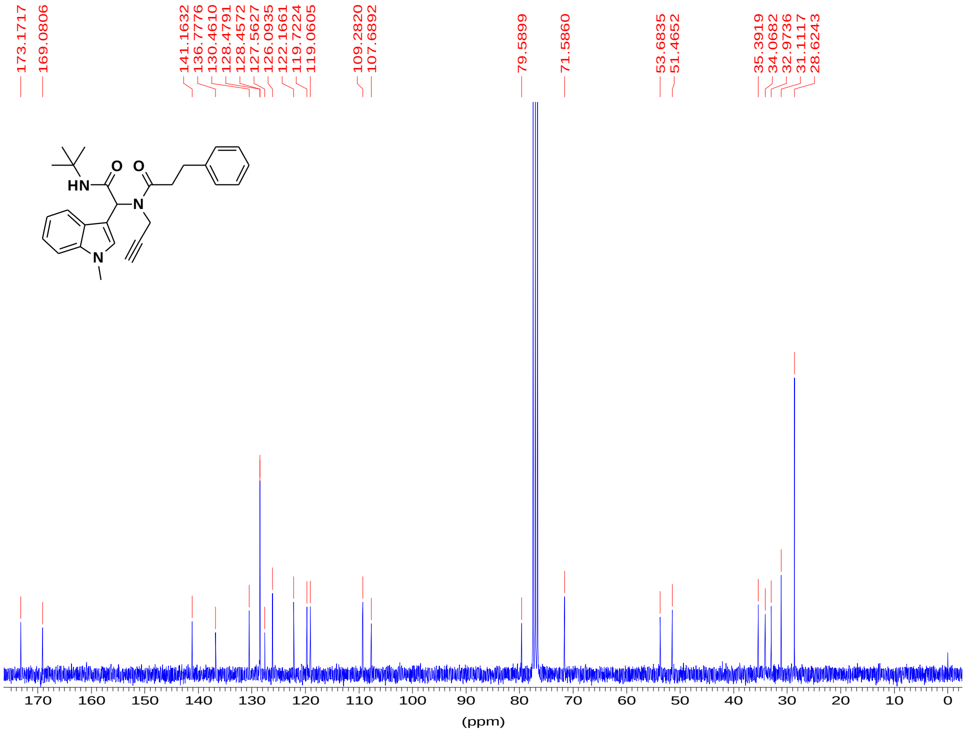

^{1}H and ^{13}C NMR spectra of compound **5p** (300 MHz, $CDCl_3$).

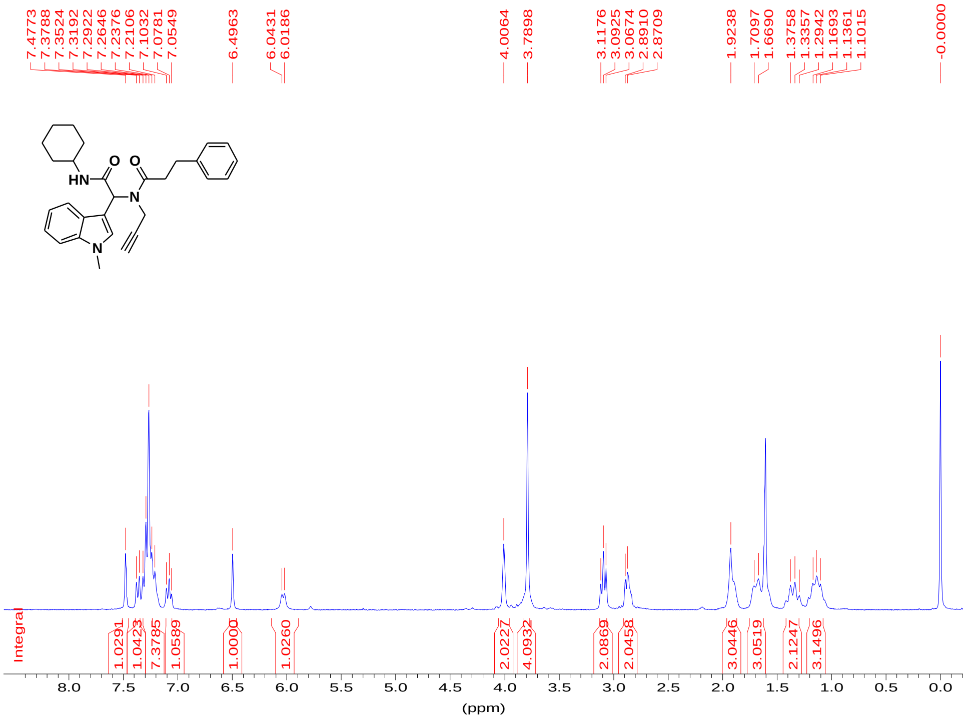

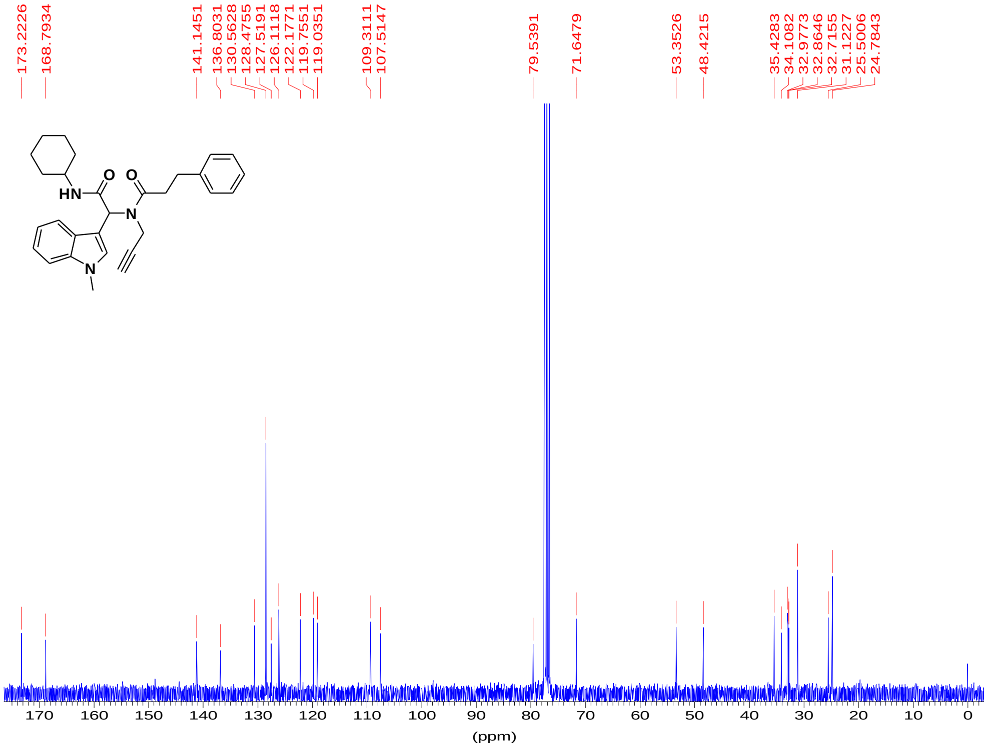

^{1}H and ^{13}C NMR spectra of compound **5q** (300 MHz, $CDCl_3$).

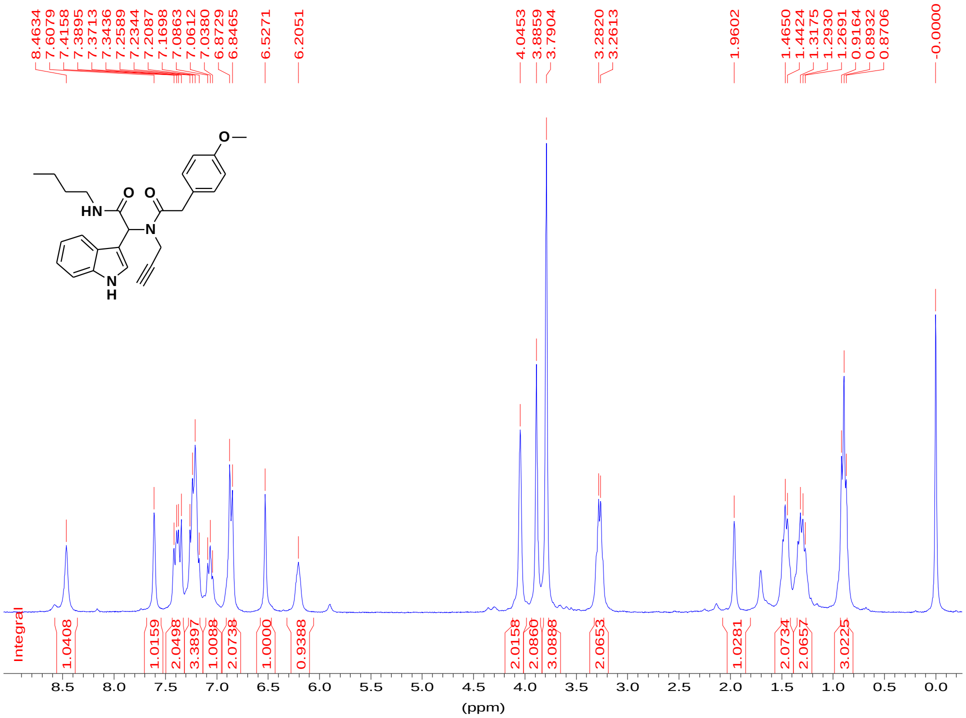

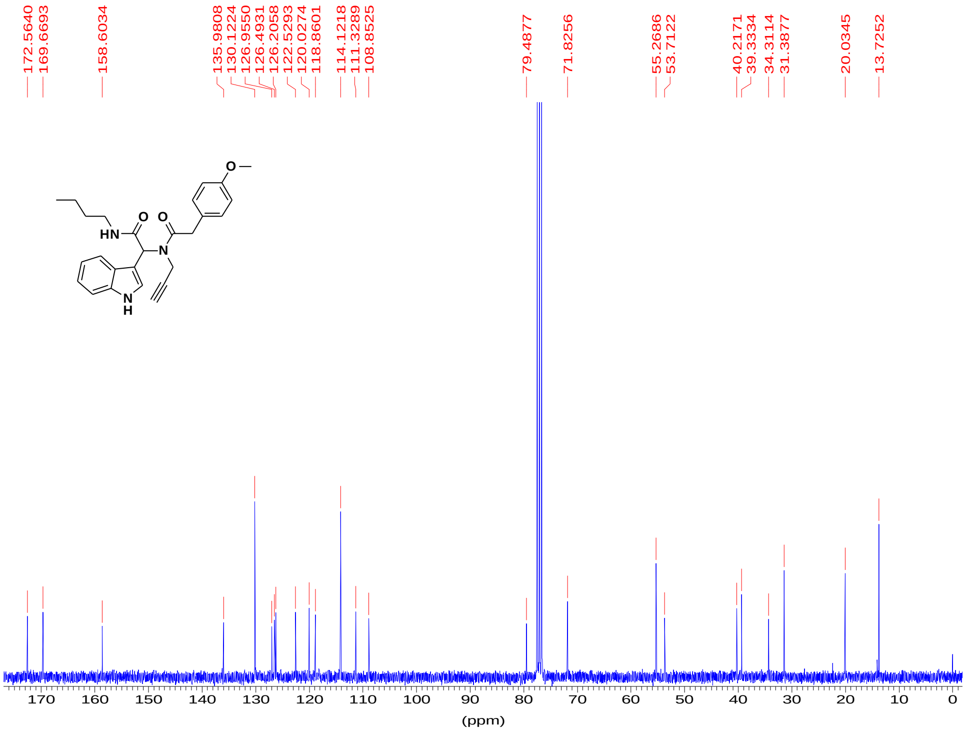

^{1}H and ^{13}C NMR spectra of compound **6a** (300 MHz, $CDCl_3$).

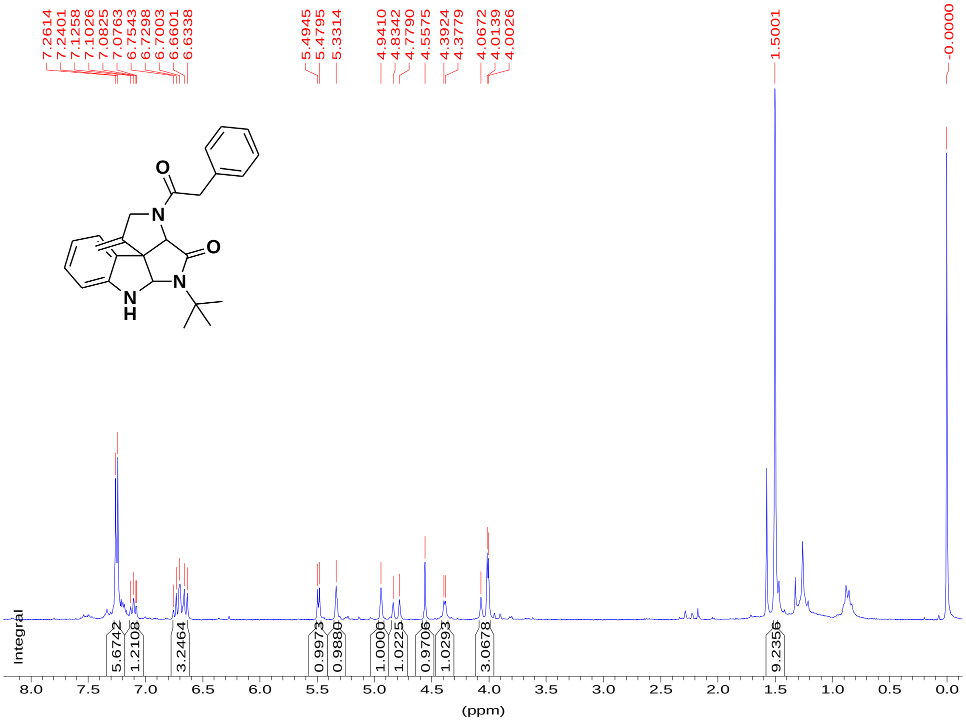

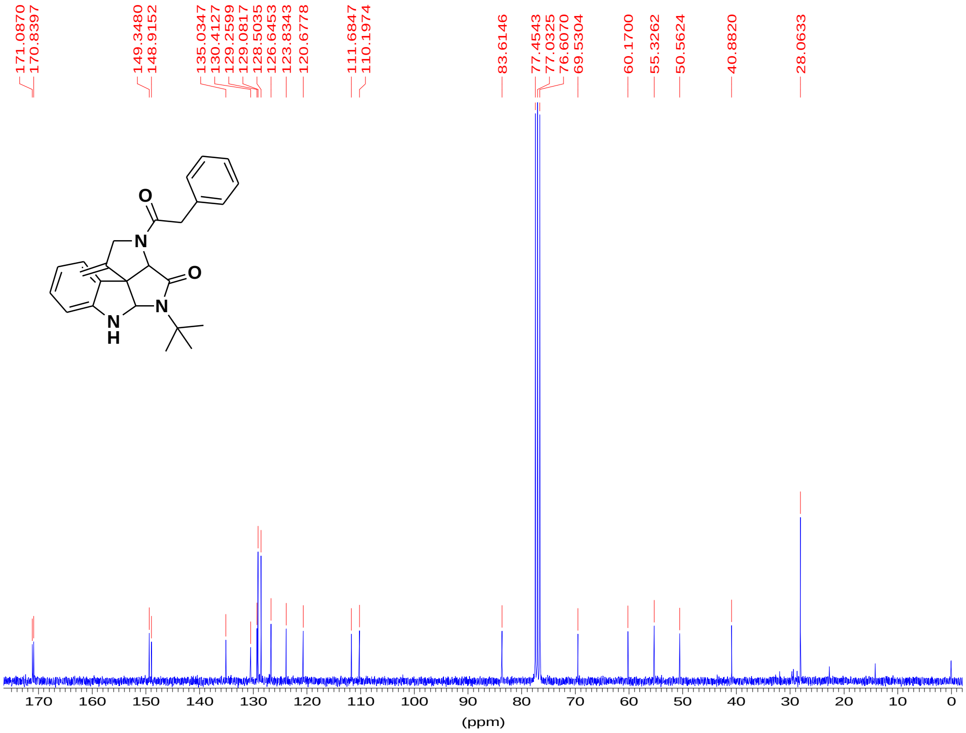

^{1}H and ^{13}C NMR spectra of compound **6b** (300 MHz, $CDCl_3$).

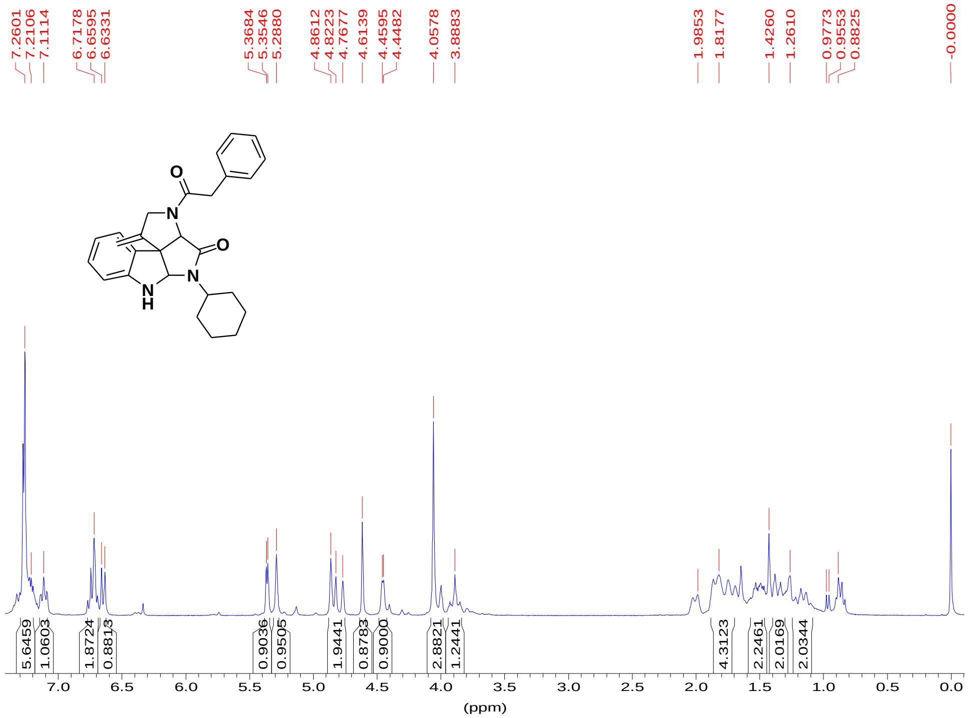

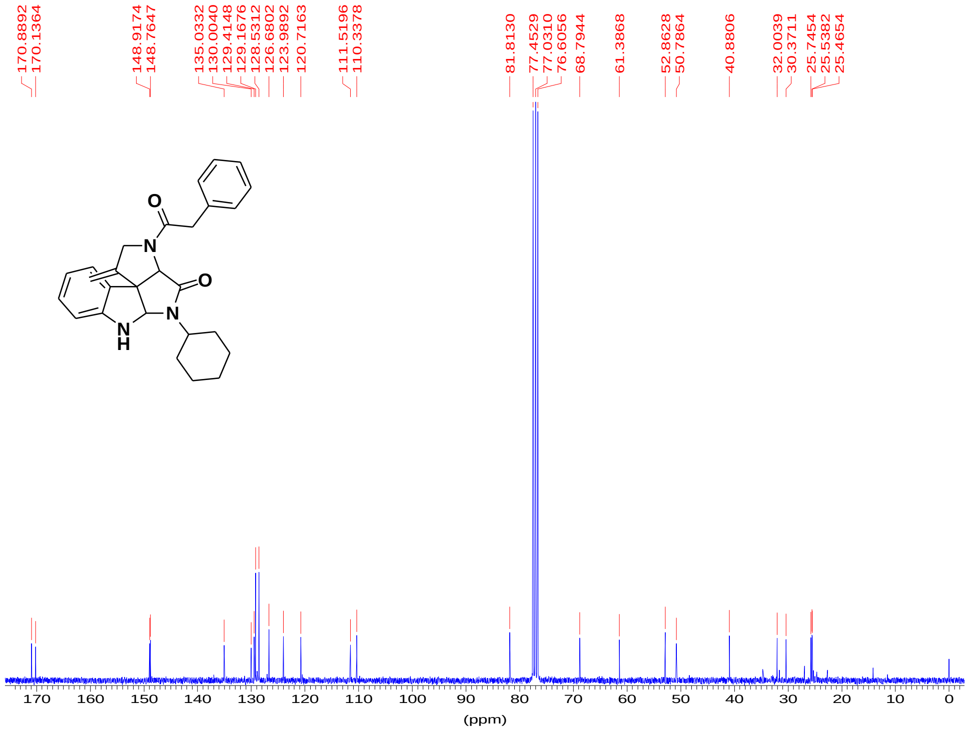

^{1}H and ^{13}C NMR spectra of compound **6c** (300 MHz, $CDCl_3$).

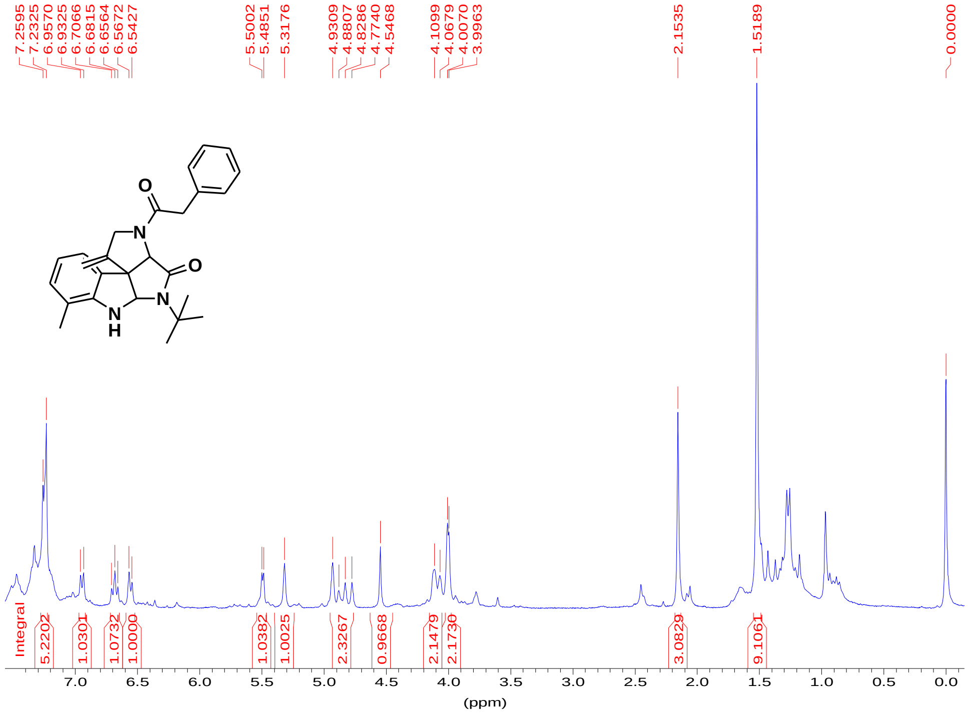

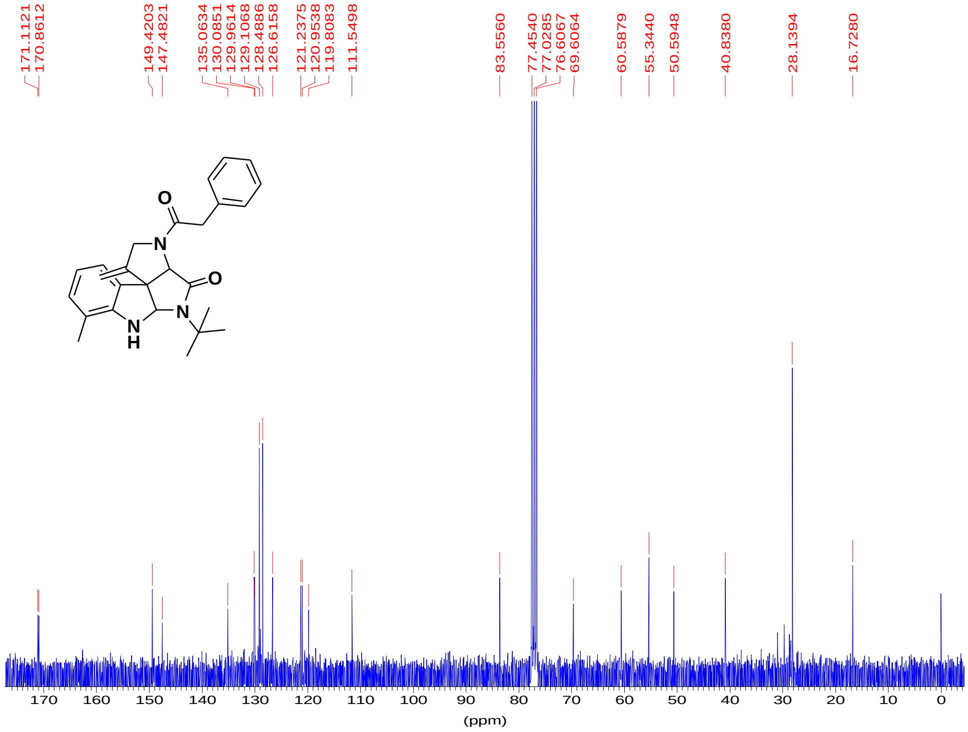

^{1}H and ^{13}C NMR spectra of compound **6d** (300 MHz, $CDCl_3$).

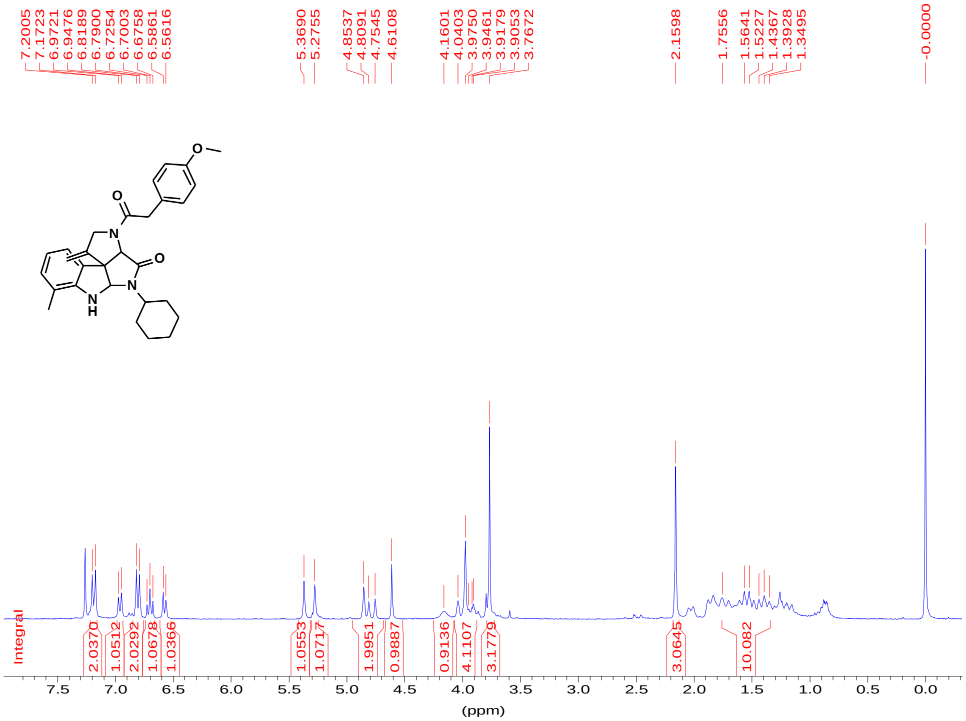

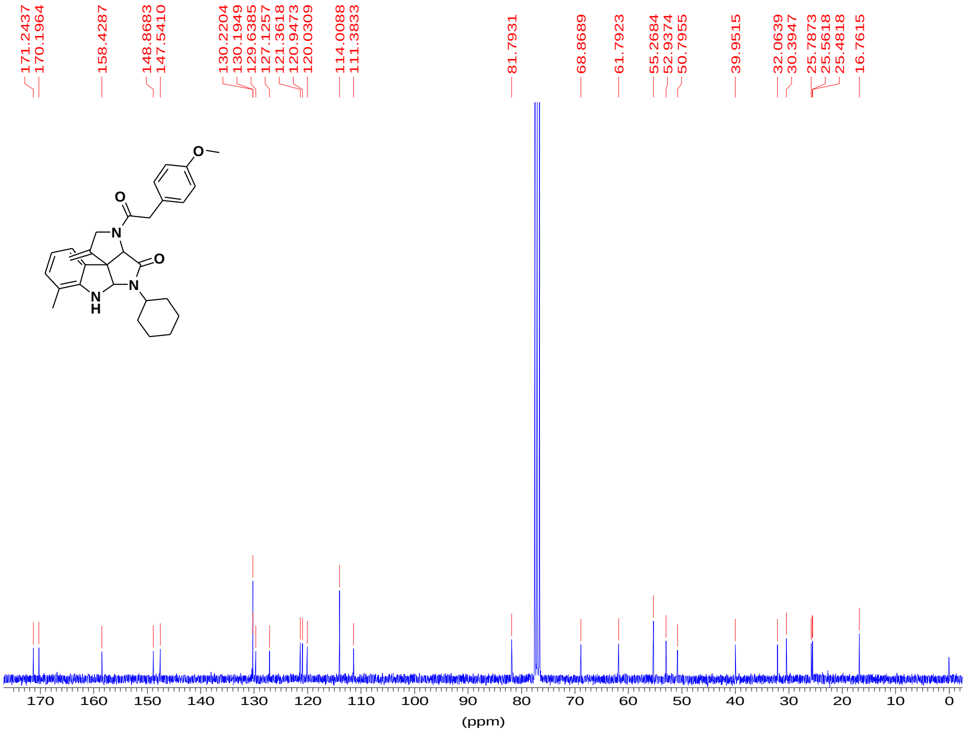

^{1}H and ^{13}C NMR spectra of compound **6e** (400 MHz, $CDCl_3$).

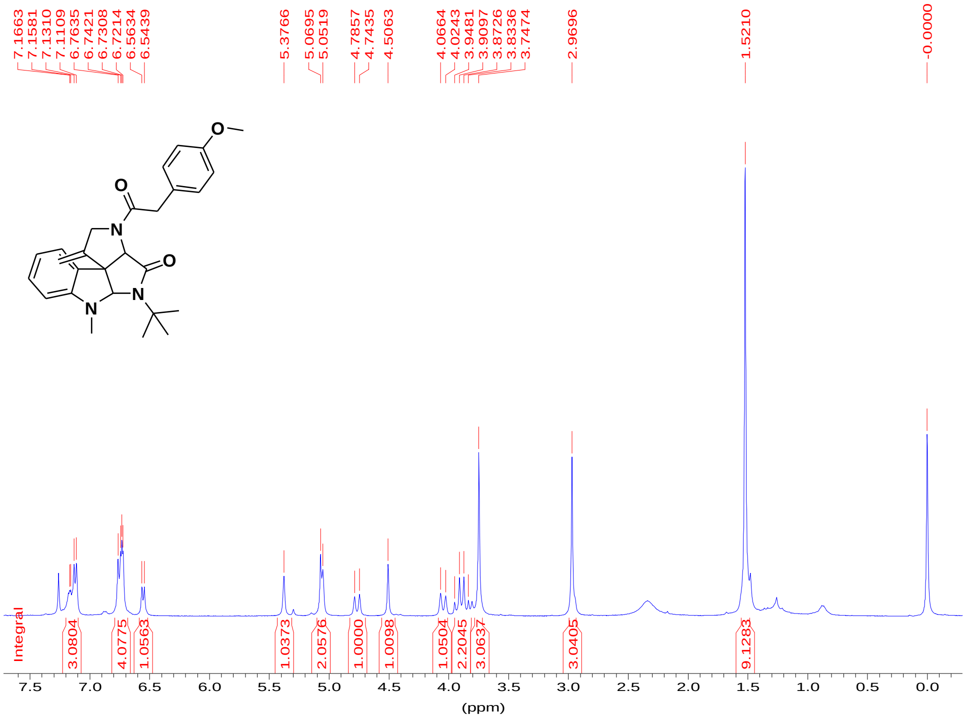

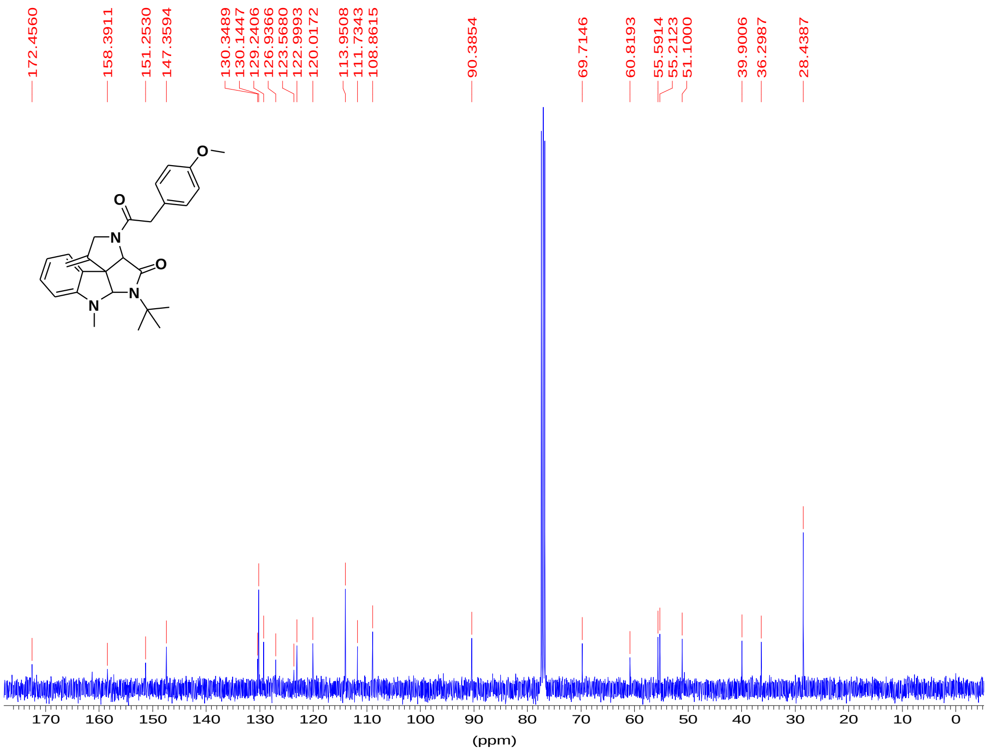

^{1}H and ^{13}C NMR spectra of compound **6f** (300 MHz, $CDCl_3$).

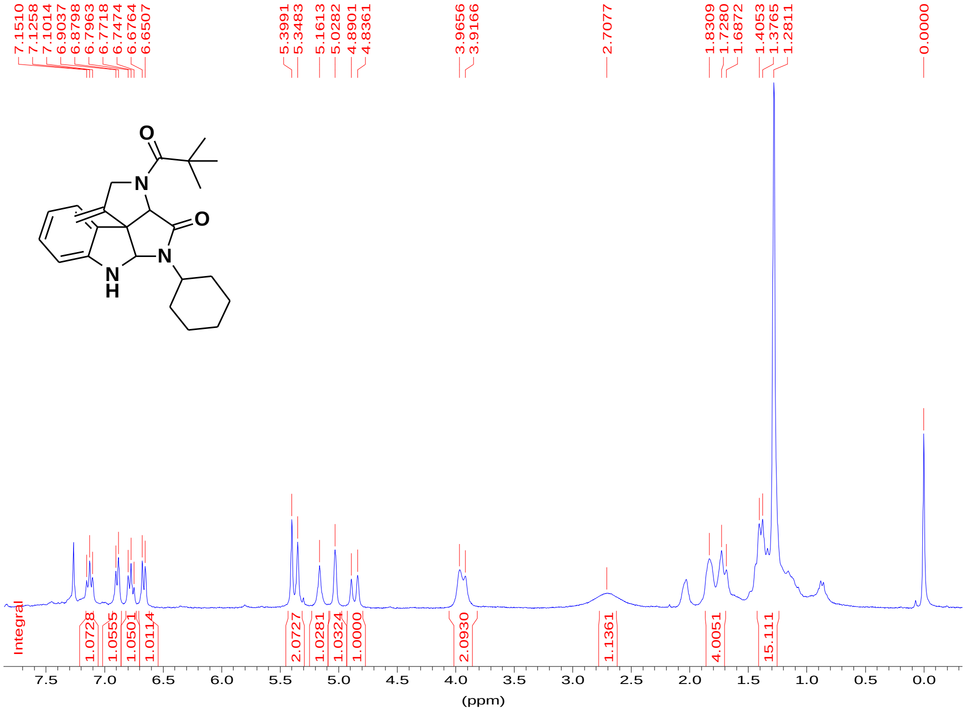

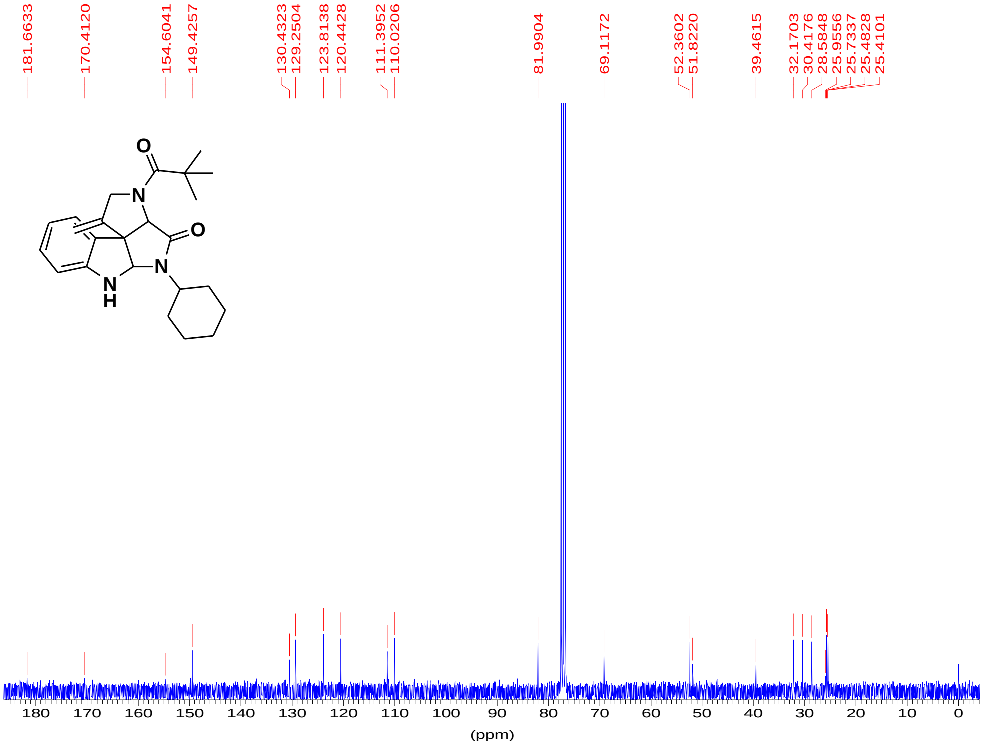

^{1}H and ^{13}C NMR spectra of compound **6g** (300 MHz, $CDCl_3$).

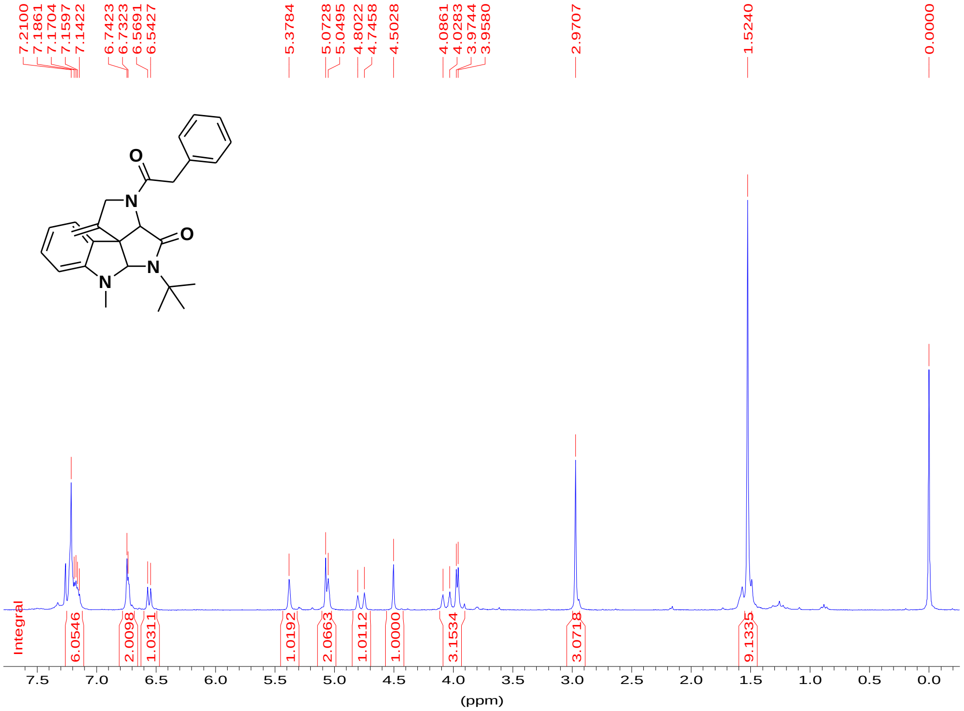

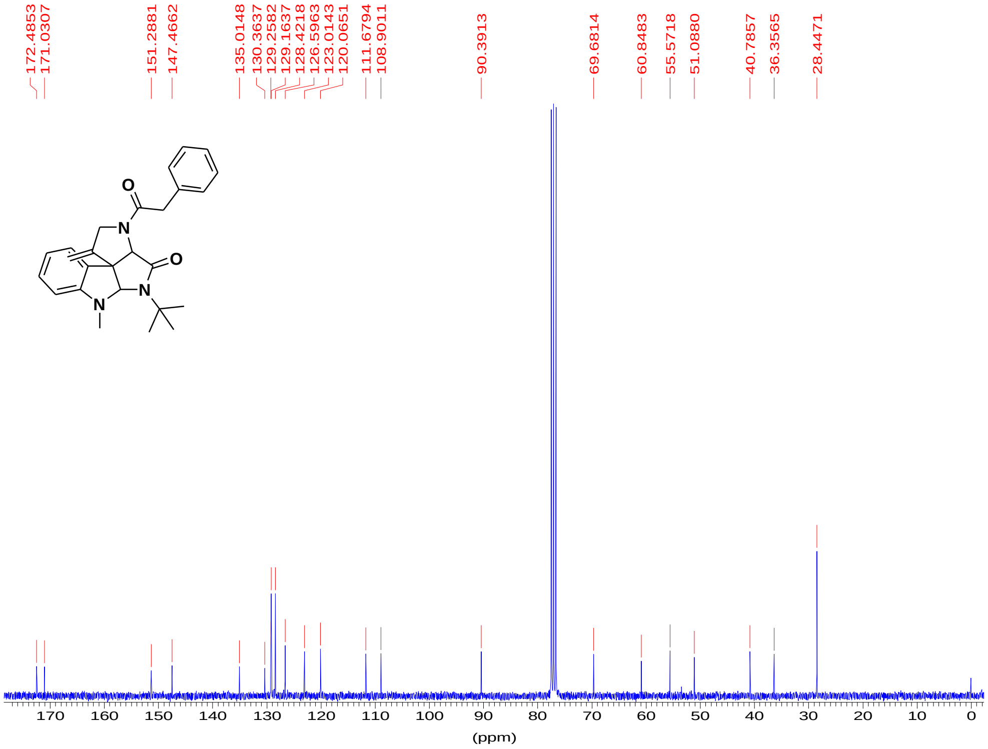

^{1}H and ^{13}C NMR spectra of compound **6h** (300 MHz, $CDCl_3$).

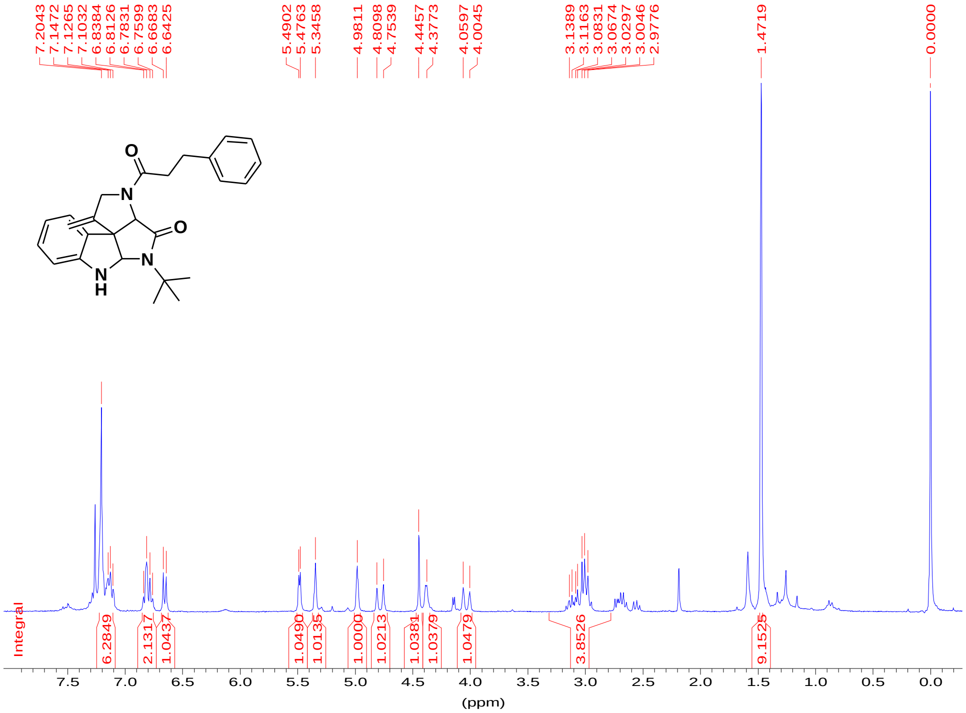

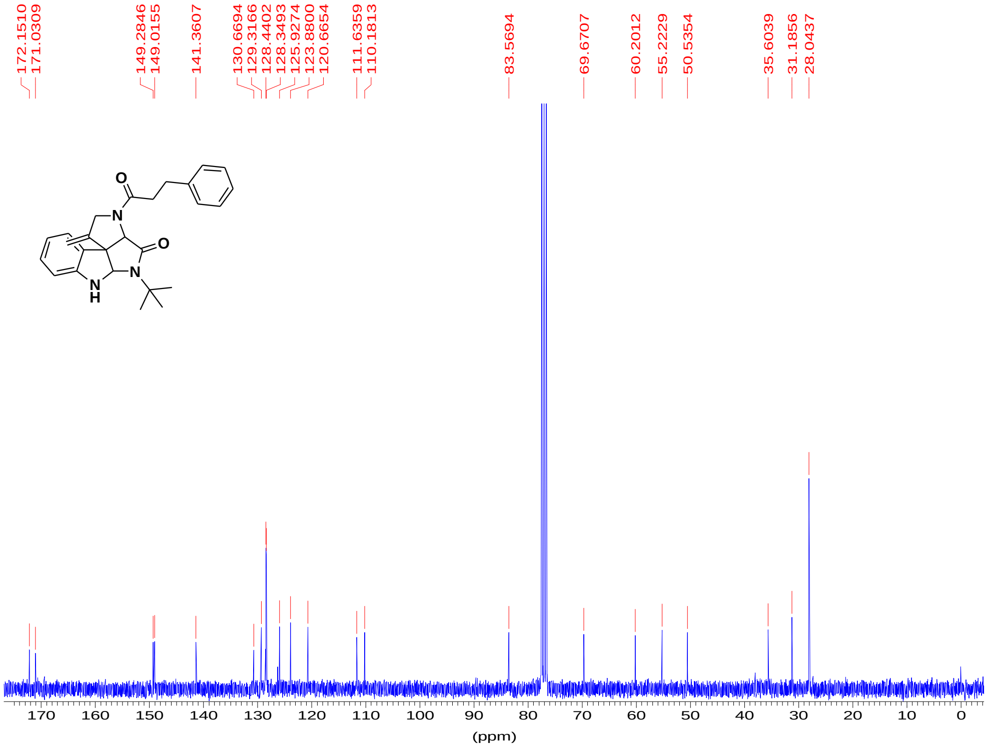

^{1}H and ^{13}C NMR spectra of compound **6i** (300 MHz, $CDCl_3$).

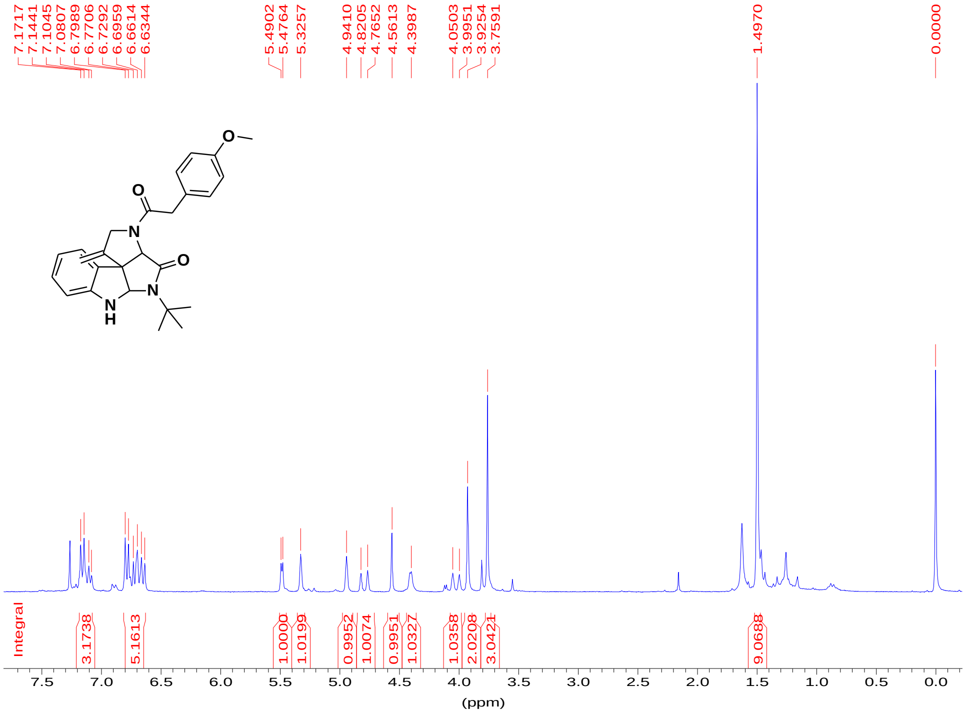

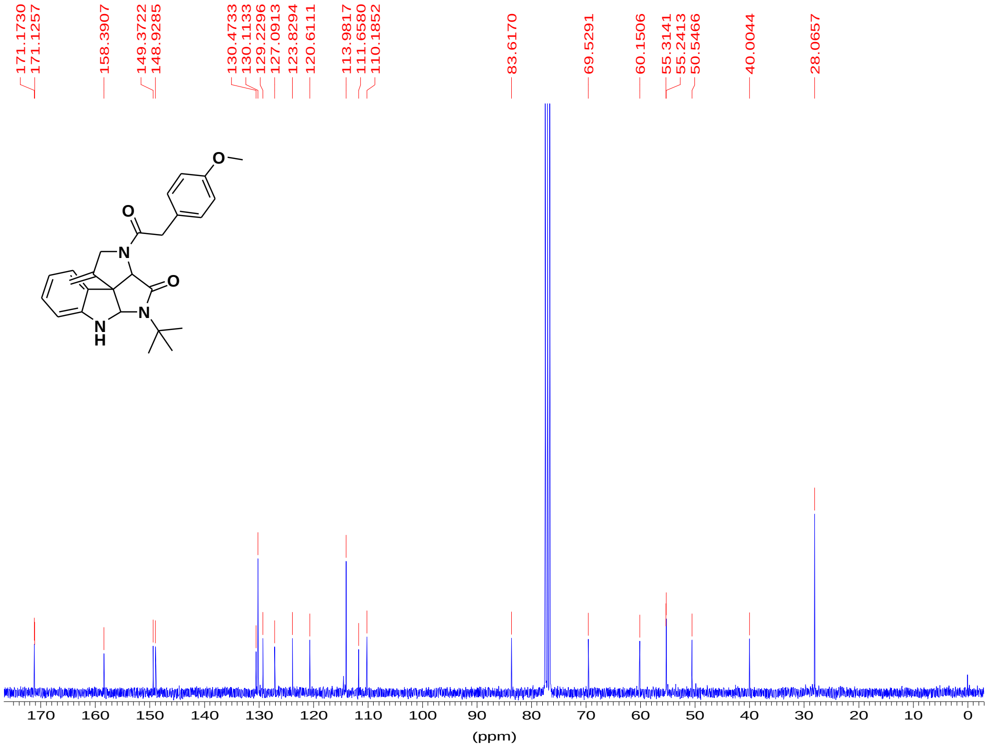

^{1}H and ^{13}C NMR spectra of compound **6j** (300 MHz, $CDCl_3$).

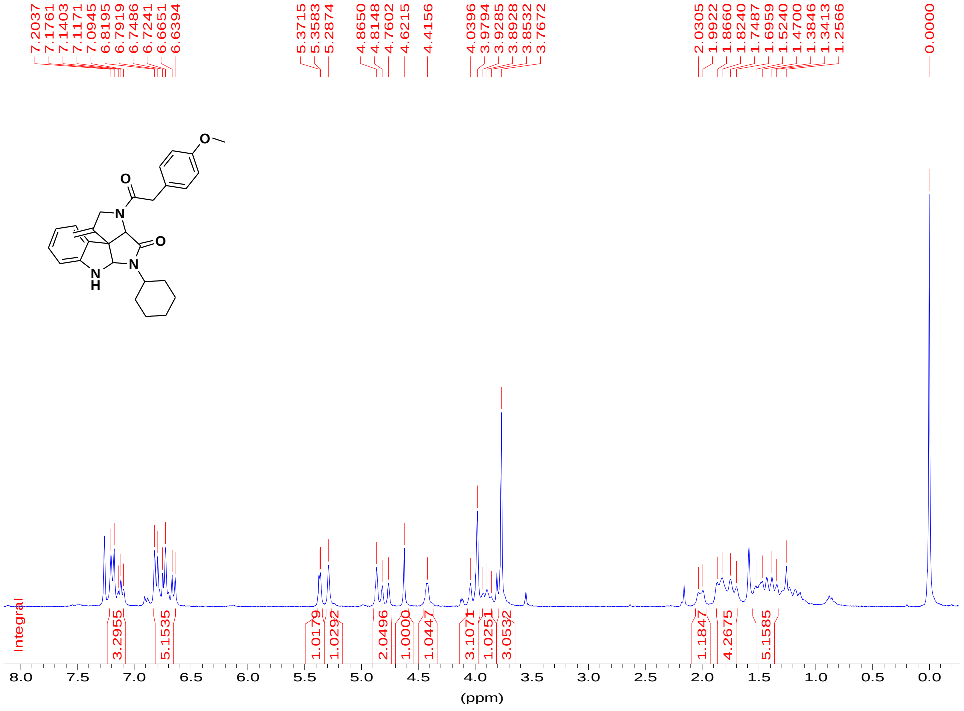

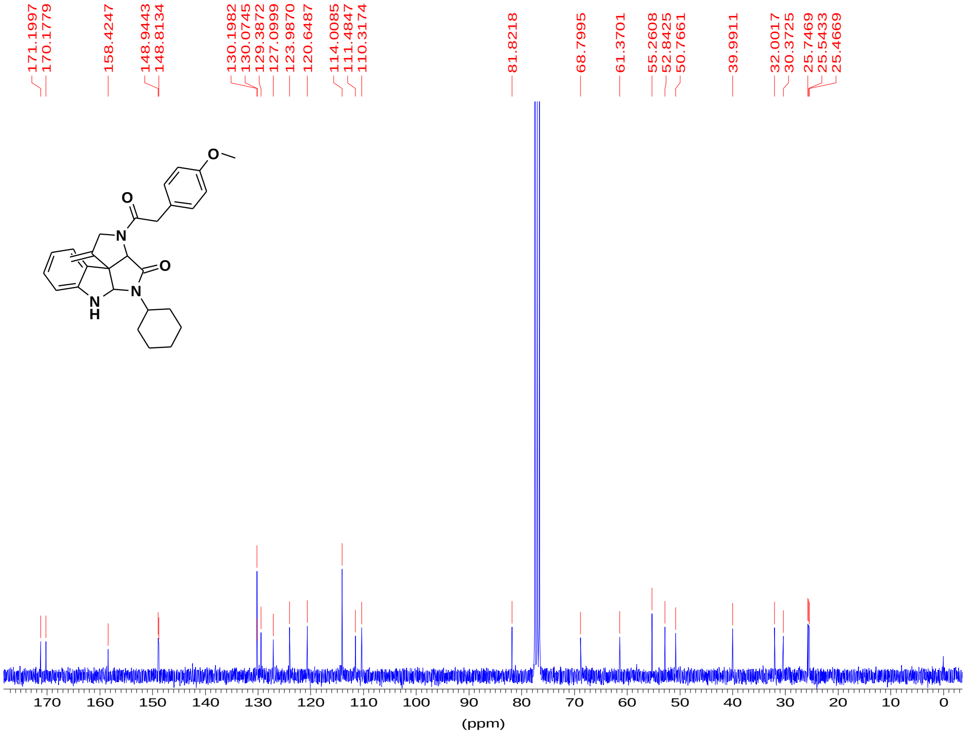

^{1}H and ^{13}C NMR spectra of compound **6k** (300 MHz, $CDCl_3$).

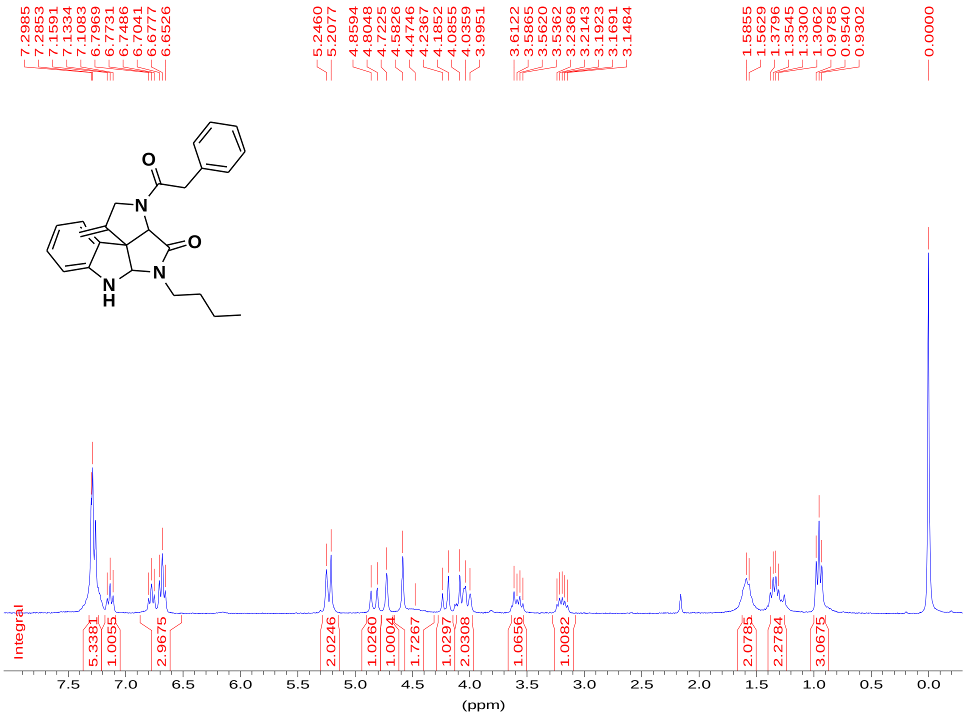

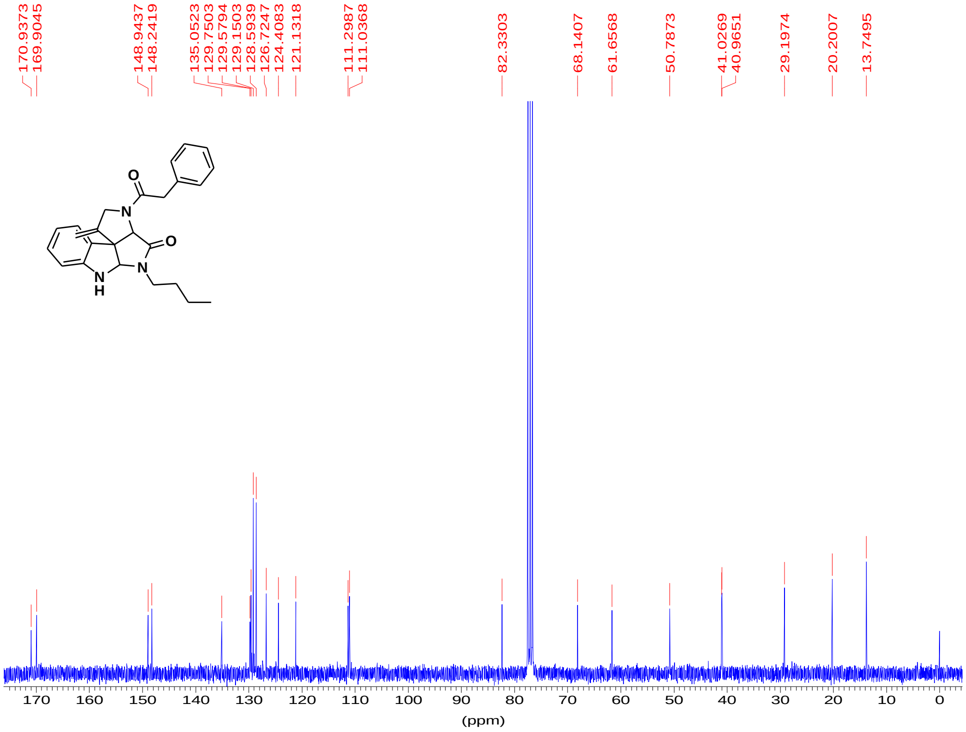

^{1}H and ^{13}C NMR spectra of compound **6l** (300 MHz, $CDCl_3$).

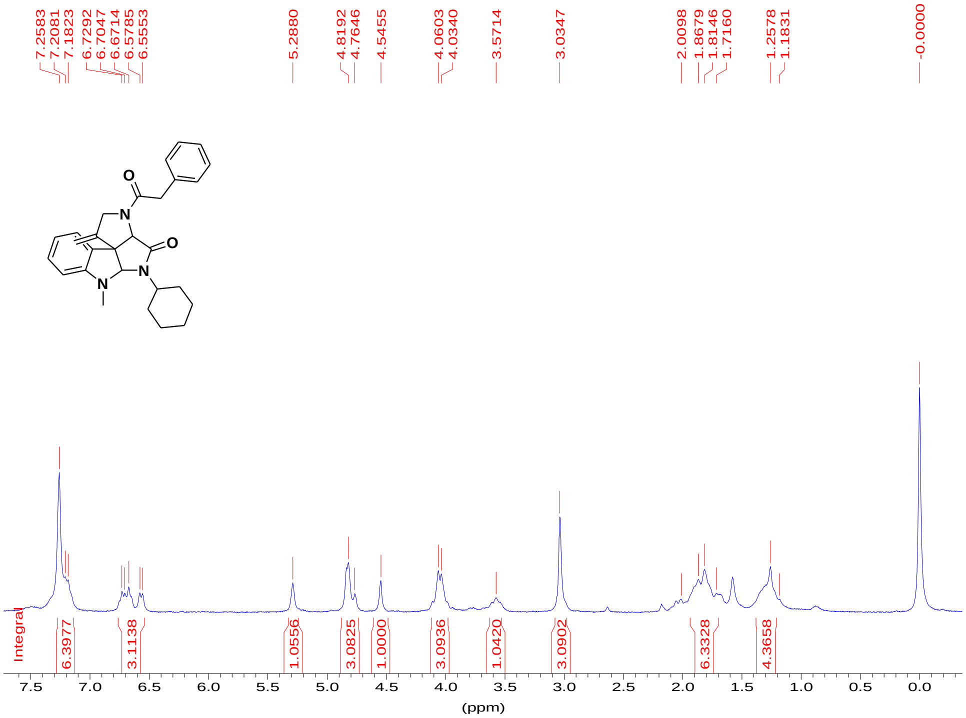

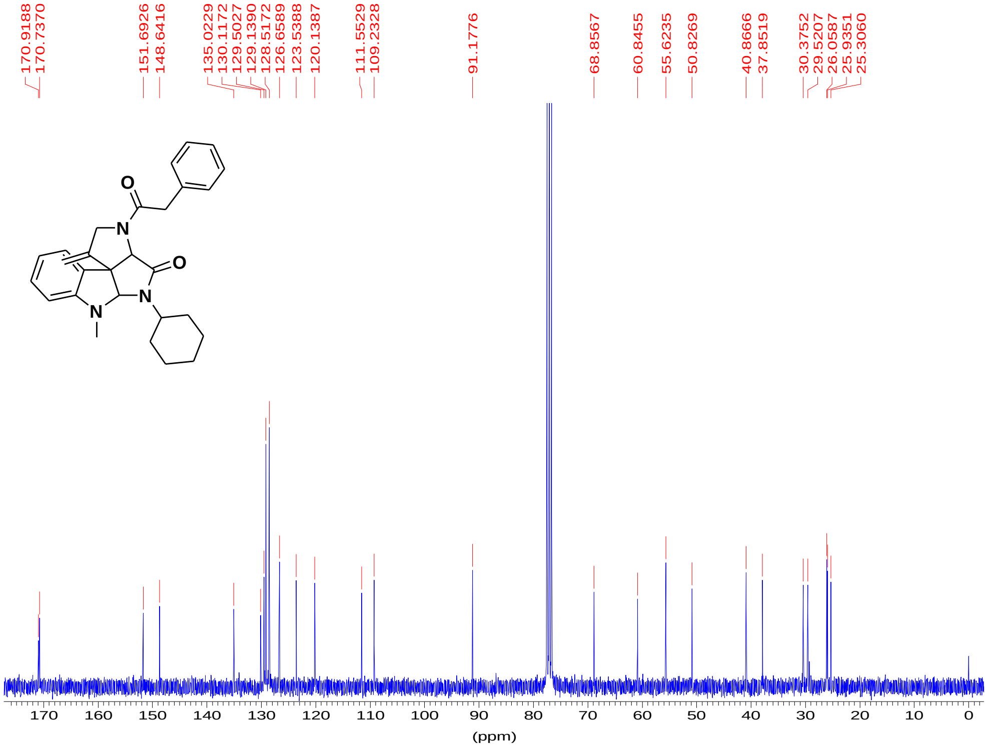

^{1}H and ^{13}C NMR spectra of compound **6m** (300 MHz, $CDCl_3$).

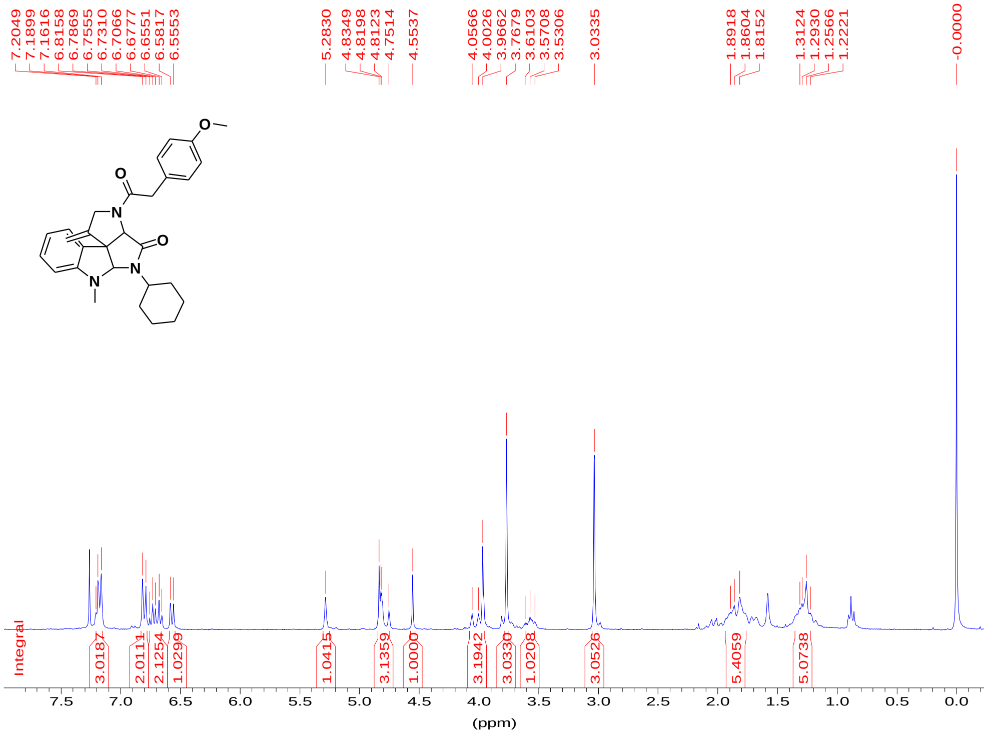

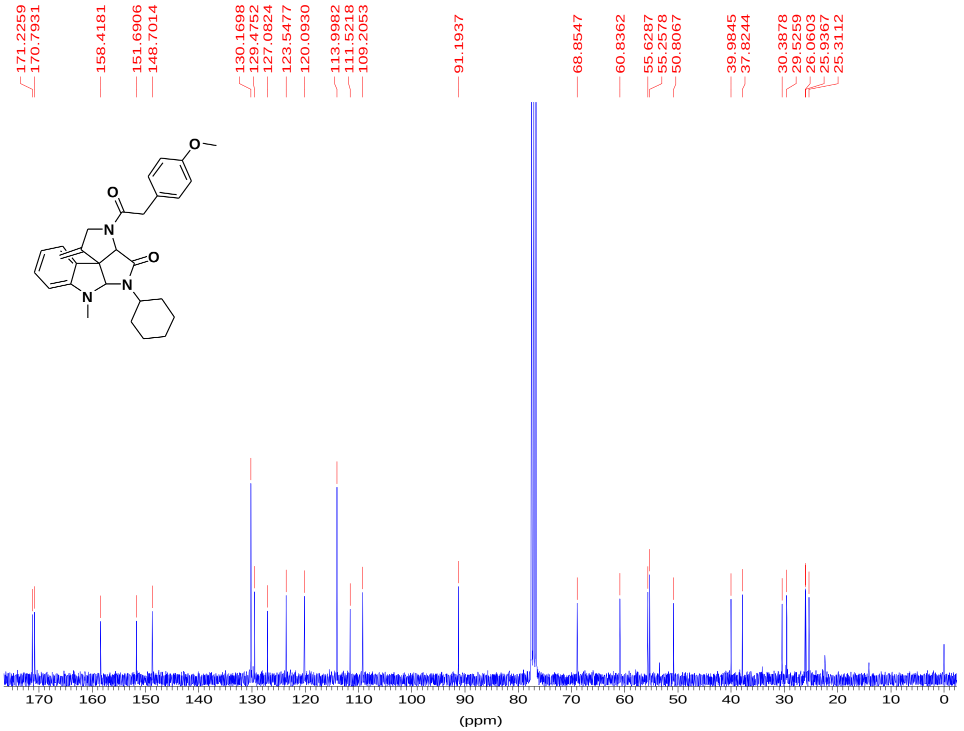

^{1}H and ^{13}C NMR spectra of compound **6n** (300 MHz, $CDCl_3$).

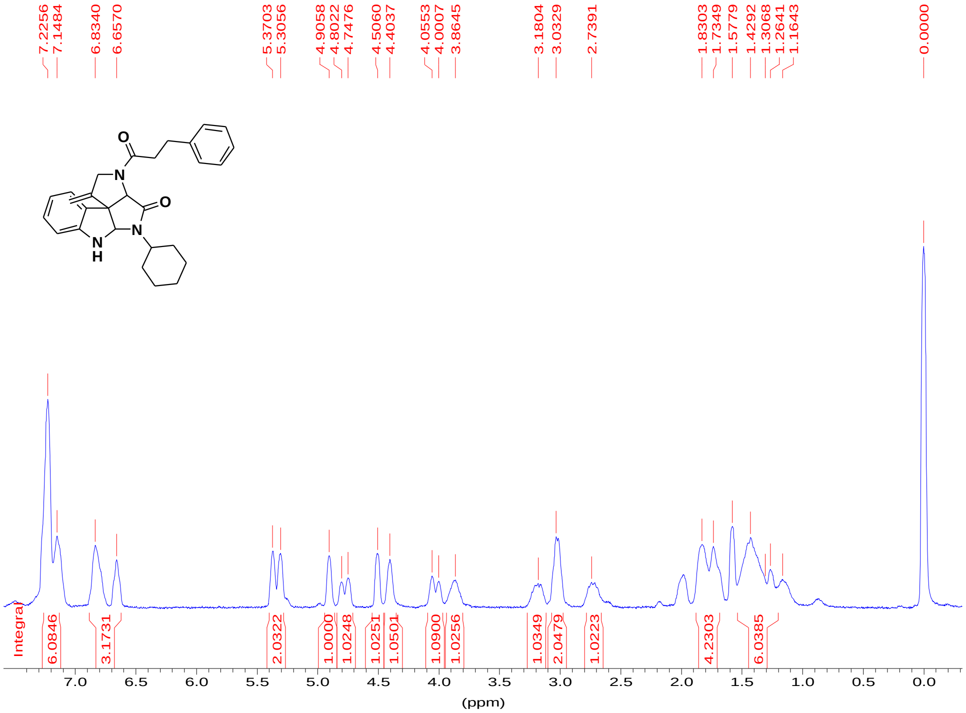

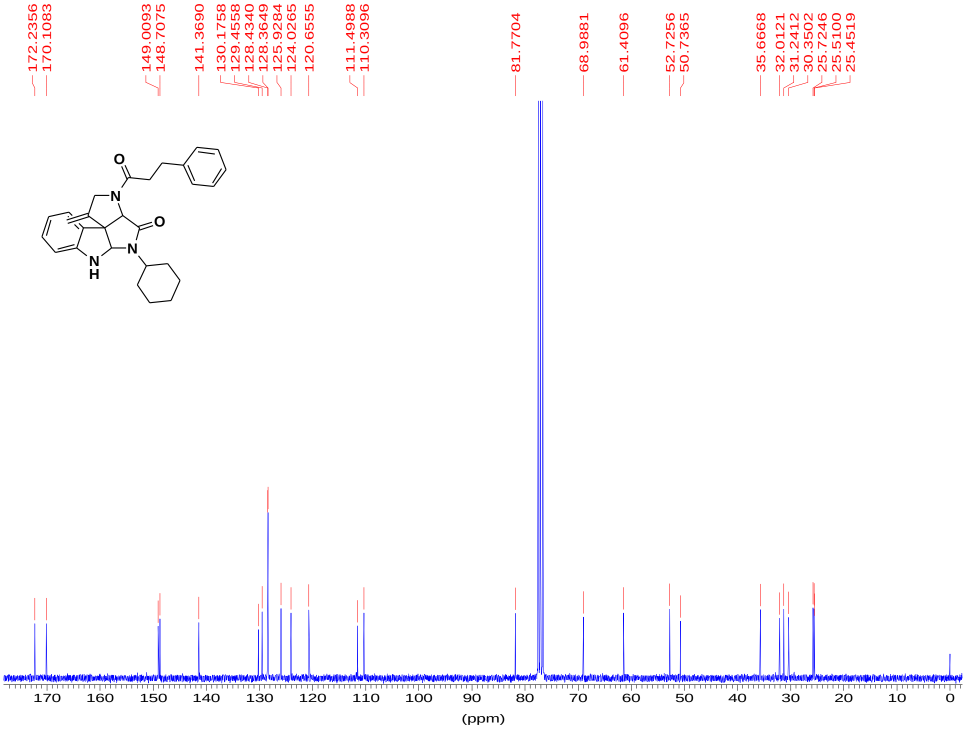

^{1}H and ^{13}C NMR spectra of compound **6o** (300 MHz, $CDCl_3$).

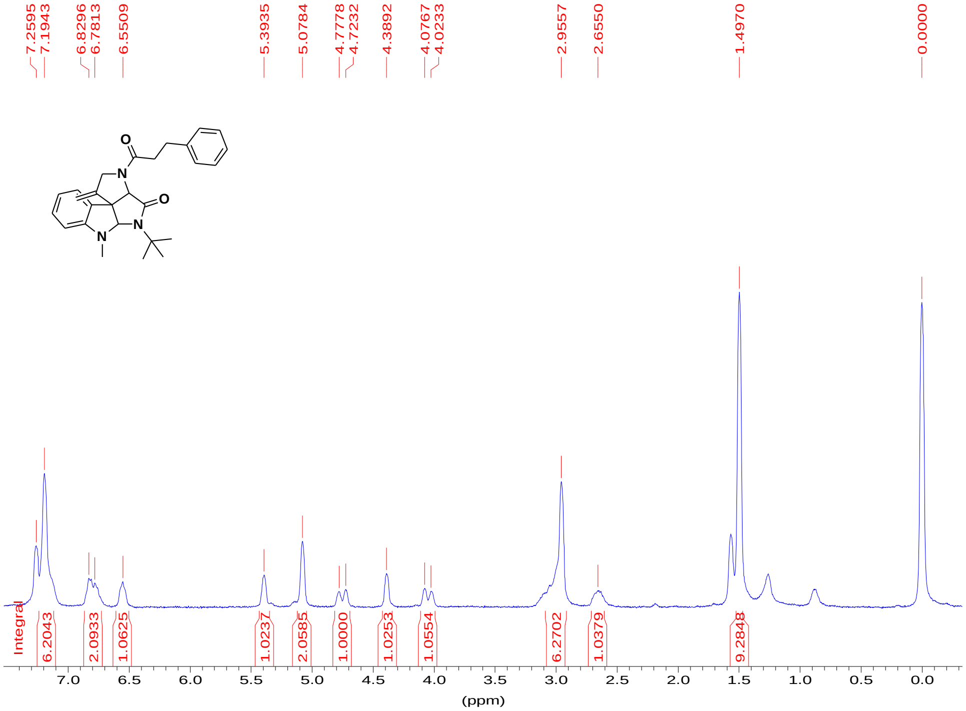

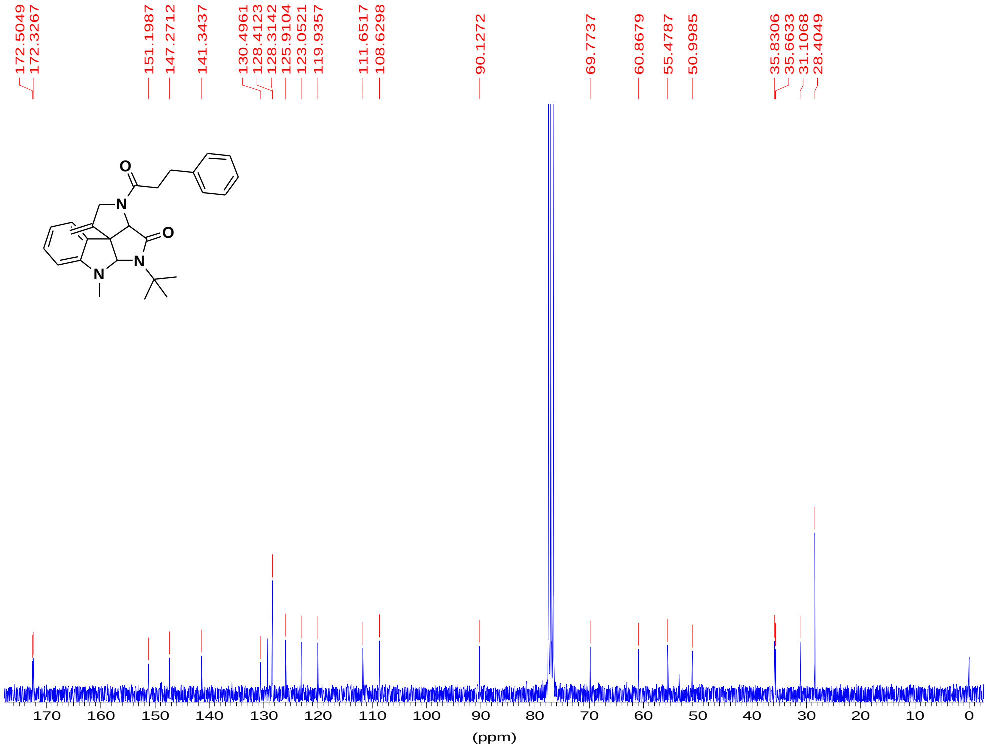

^{1}H and ^{13}C NMR spectra of compound **6p** (300 MHz, $CDCl_3$).

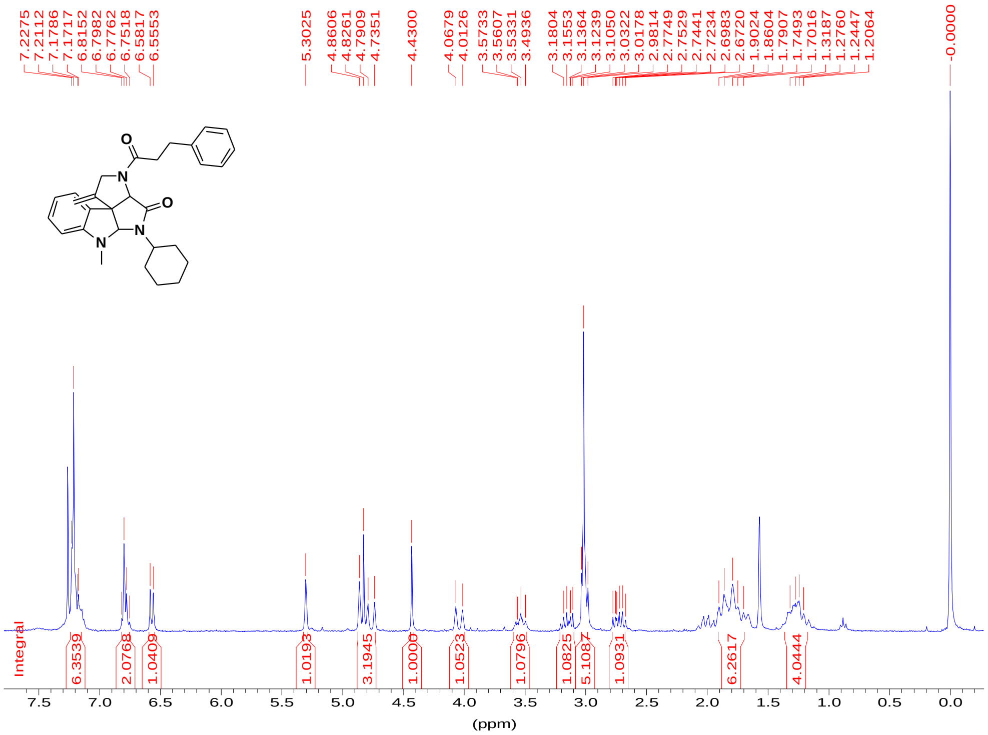

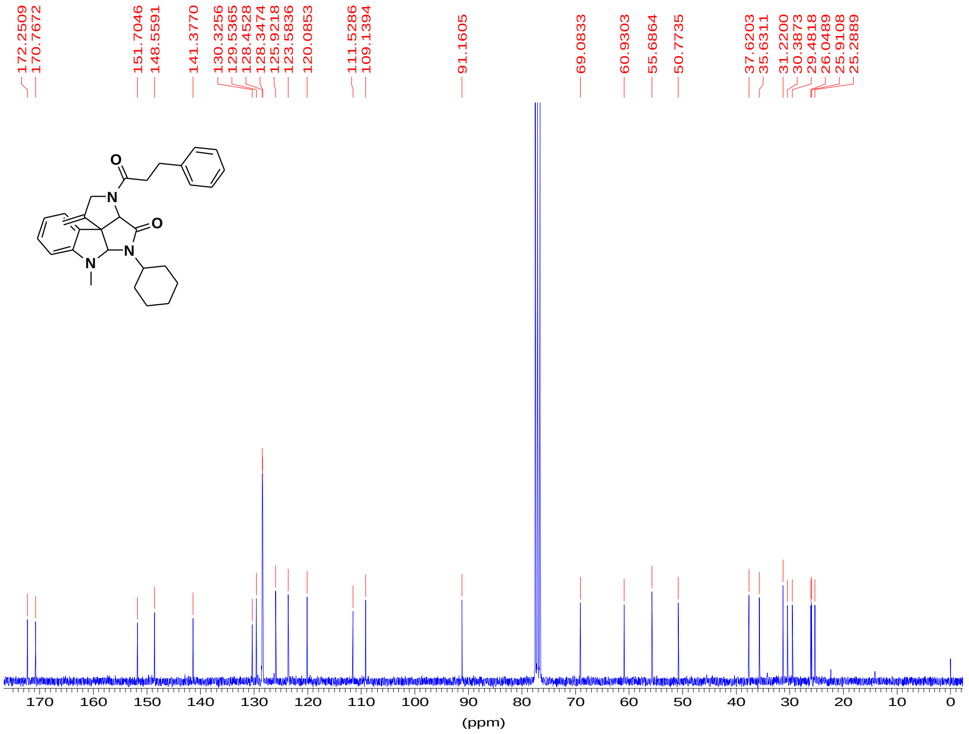

^{1}H and ^{13}C NMR spectra of compound **6q** (300 MHz, $CDCl_3$).

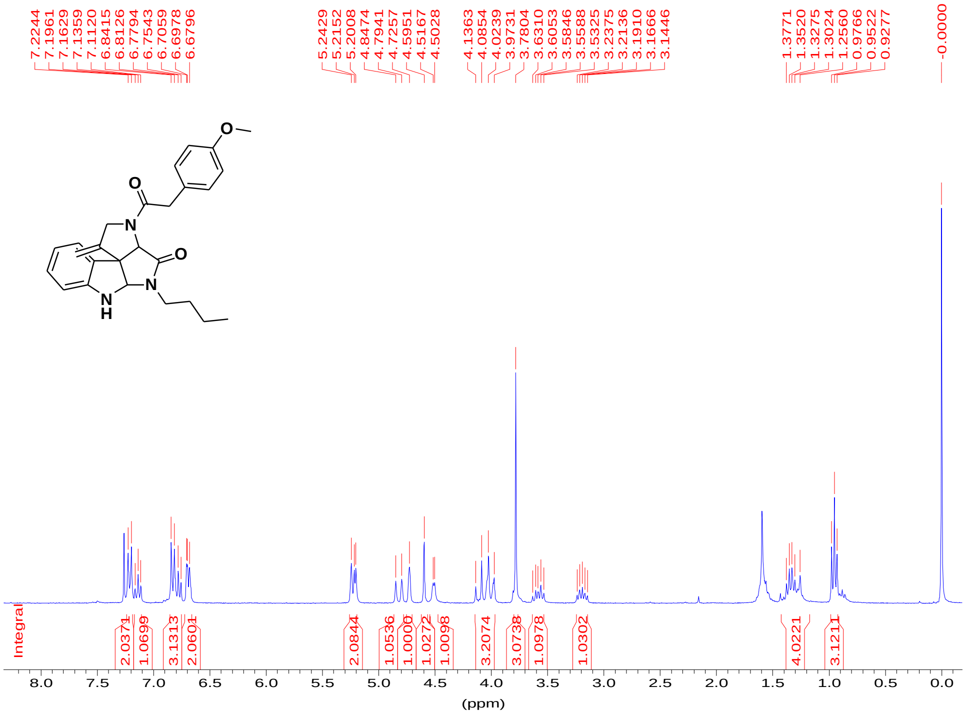

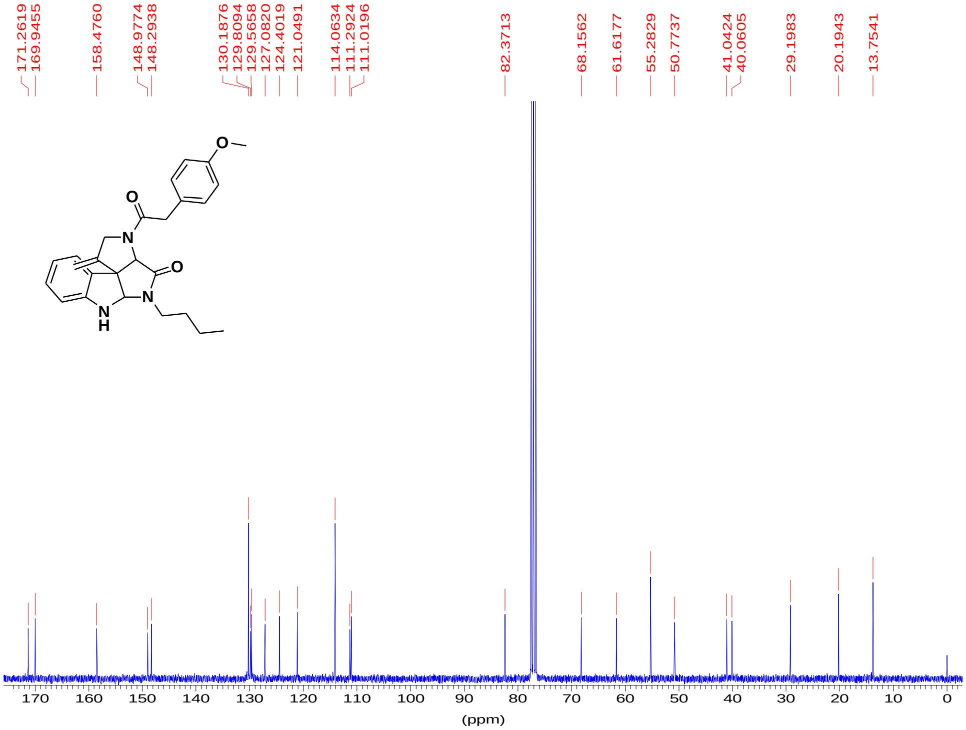

Supplement: File 1 — Experimental section. [file Beilstein_J_Org_Chem-09-2097-s001.pdf]
